# Synthesis of 2,7-Dihydrooxepine-spirodithiophene Derivatives via an Intramolecular Ring Closure Reaction

**DOI:** 10.1021/acsomega.4c07409

**Published:** 2024-12-17

**Authors:** Xiaochen Liu, Tian Gao, Ruiyao Wang, Jianan Liu, Hua-Jun Shawn Fan, Jin Fang, Chang-Qi Ma, Yi Lin

**Affiliations:** † Department of Chemistry and Materials Science, Xi’an Jiaotong-Liverpool University, Suzhou, Jiangsu 215000, P. R. China; ‡ Department of Chemical Engineering, Sichuan University of Science & Technology, Zigong, Sichuan 643000, P. R. China; § i-Lab & Printable Electronics Research Center, Suzhou Institute of Nano-Tech and Nano-Bionics, Chinese Academy of Sciences, Suzhou 215123, P. R. China; ∥ Wisdom Lake Academy of Pharmacy, Xi’an Jiaotong-Liverpool University, Suzhou, Jiangsu 215000, P. R. China

## Abstract

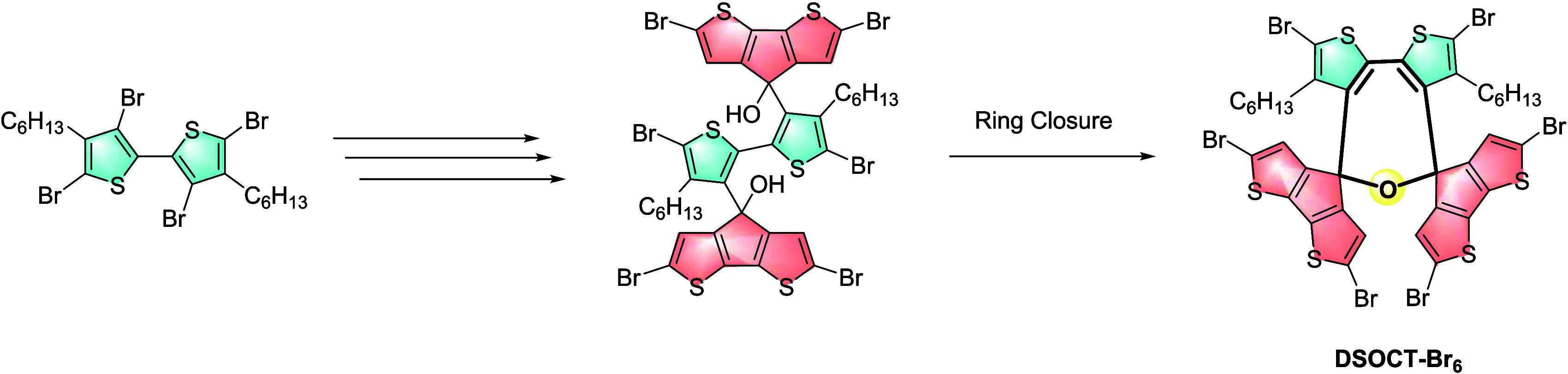

Spiro architectures with π-conjugation have improved thermal stability and stronger photosensitivity, making them potentially useful for organic optoelectronic devices. Our recent work has demonstrated the synthetic chemistry of a novel thiophene oligomer combining 2,7-dihydrooxepine and dispiro structure and derived it into A–D–A-type compounds. The optical spectroscopy and electrochemical characteristics were investigated. The results show that the presence of the alkyl side chains enhances the nucleophilicity of aromatic anions but induces strong steric hindrance so that the selectivity toward a dispiro[cyclopenta[2,1-b:3,4-b’]dithiophene-4,4′-dithieno[3,2-c:2′,3′-*e*]oxepine-6′,4″-cyclopenta[2,1-b:3,4-b’]dithiophene] (DSOCT) core is preferred. The A–D–A-type DSOCT derivatives show an increased light absorption wavelength and a reduced optical band gap. The TD-DFT study exhibited consistent results with the experimental analysis. Regarding application to organic solar cells of both materials, **PM6:DSOCT-(TFIC)**_**6**_-based solar cells exhibited better power conversion efficiency (PCEs) compared to **PM6:DSOCT-(TIC)**_**6**_-based devices. This improvement can be attributed to the higher current density and fill factor, which are facilitated by the more efficient charge excitation, separation, and transport resulting from the molecular “fluorination effect.″.

## Introduction

Spiro-conjugated molecules are promising structures in which two π-systems are orthogonally bonded to one common sp^3^ carbon. Their advantages of structural symmetry and rigidity with small reorganization energy has led to a great interest in optoelectrical applications.^1−3^ As illustrated by Simons and Fukunaga,^4^ the unique orthogonal π-systems reduce the intermolecular aggregation and enhance the carrier mobility compared to planar analogues, which are beneficial for organic light-emitting diodes (OLEDs),^5^ organic field-effect transitions (OFETs),^6^ and organic photovoltaics (OPVs).^7^ Conventionally, building up a 9,9′-spirobifluorene (SBF) core normally involves nucleophilic addition of a lithiated biaryl intermediate to 9-fluorenone, followed by Lewis acid-catalyzed intramolecular Friedel-Craft alkylation.^8^ Fused-thiophene is a common building block in the development of organic semiconductors that possess better geometrical planarity and stronger carrier transportation capability in comparison with fused-benzene analogues.^9−14^ Construction of 4,4′-spirobi[cyclopenta[2,1-b;3,4-b’]dithiophene] (SCT) core is also done using a similar approach to that for **SBF**.^15^ However, in our previous work, a diol side product was collected in synthesizing SCT-cored derivatives.^16^ Regrettably, we did not investigate the chemistry of the diol. In addition, we found that the intramolecular Friedel-Craft alkylation is concentration dependent and competes with intermolecular alkylation. To address this issue, we employed (3,3′-dibromo-4,4′-dihexyl-[2,2′-bithiophene]-5,5′-diyl)bis(trimethylsilane) (**Br**_**2**_**-2TC6-TMS**_**2**_) as a precursor to build up a branched diol intermediate, which was further treated with Lewis acid to form a novel structure. The presence of alkyl side chains would efficiently improve the solubility in common organic solvents and avoid lithiation on the 4-position of the thiophene ring.^17,18^ To our surprise, the diol incurred intramolecular dehydration only toward a dihydrooxepine-based core, along with a dispiro-conformation arranged on its’ 2,7-regioposition.

**Figure 1:**
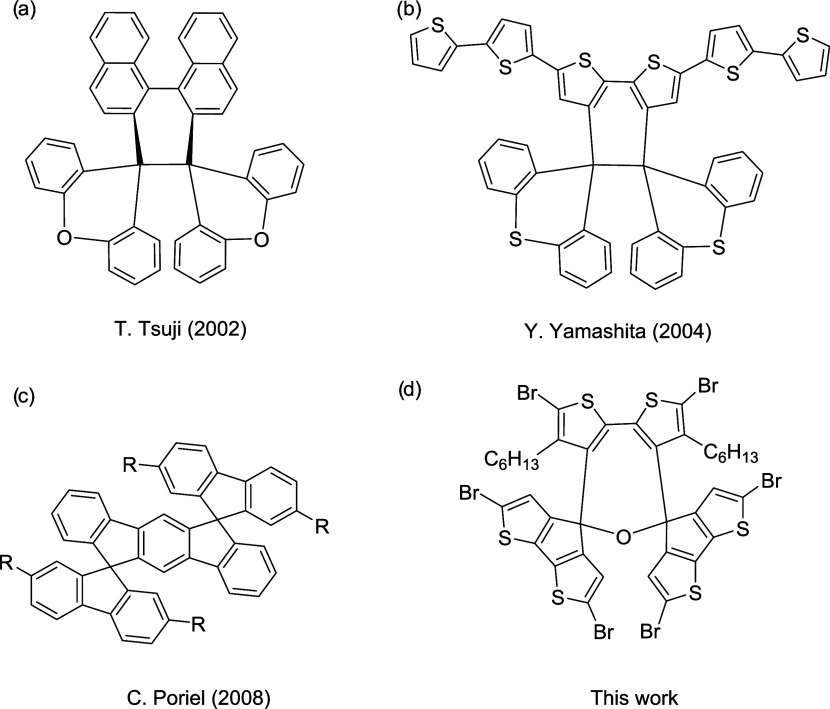
Chemical structures of hexaarylethane-based derivatives developed by Tsuji’s group (a); Yamashita’s group (b); **(1,2-b)-DSF-IFs** developed by Poriel (c); and 2,7-dihydrooxepine-cored architecture regarding this work (d).

Dispiro building blocks are shape-persistent architectures that include orthogonal squares, tubes, and ladder structures.^19,20^ For example, Wei and co-workers synthesized two H-shaped molecules **TBPDSFDITF** and **TDOF-DSFDITF**, both rigid conformations presenting a high quantum efficiency of 80%.^19^ Besides, Poriel et al. developed a dispiro-molecule **(1**,**2-b)-DSF-IFs** (Figure 1) with high thermal stability (*T*_g_ = 350 °C), which is applied to blue OLED.^21^ Takagi’s group prepared 9-fluorene-type trispirocyclic compounds for hole-transporting material (HTM) in electron luminescence (EL) device application.^22^ In comparison with a single spiro-π system, dispiro-based π-systems can serve as a better chromophore for stronger light absorption, and a more rigid skeleton is featured with higher thermal stability as well as easier carrier transportation.^23,24^ Such characteristics would allow them to have a potentially better performance than normal spiro architectures if used as optoelectrical materials. In 2002, Tsuji and co-workers successfully synthesized a racemic hexaarylethane derivative (Figure 1) with a helical π-skeleton in four steps. Electrochemical testing results show that this molecule exhibits strong electron-donating characteristics to form redox pairs with its oxidated form and can be potentially used as an electrochiroptical material.^25^ Yamashita in 2004 synthesized bithiophene-hexaarylethane, which shows strong electrochemical stability. The presence of thiophene oligomer makes this structure easy to modify and possibly be used as a molecular wire.^26^ Based on literature review, we found that no examples regarding dispiro-ladder-type conformations bearing oxepine-based heterocycles are reported. Therefore, it is still worthwhile to enrich the family of spiro-moieties. In this work, the formation of diol and subsequent intramolecular dehydration toward dihydrooxepine was found to be the major selectivity for cascade steps. Single-crystal X-ray diffraction provided a clear view for special arrangement of the two cyclopentadithiophene (CPDT) units in the dispiro-based skeleton, which is quite different from the orthogonal arrangement. Thus, this work discusses the chemistry along with optoelectrical properties in detail.

## Results and Discussion

**Scheme 1:**
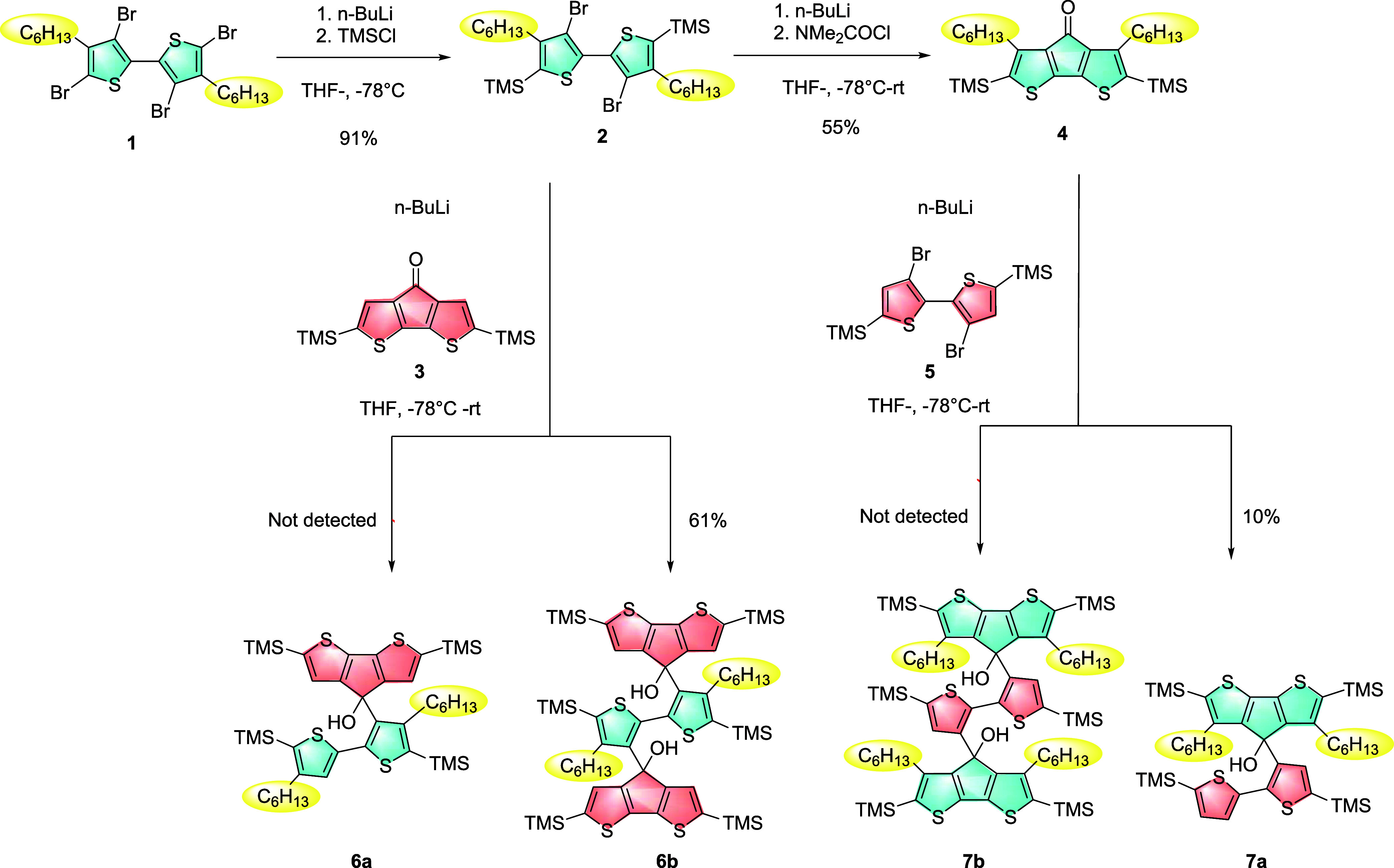
Synthetic Pathway Towards All Intermediates of **6a**, **6b**, **7a** and **7b**

As shown in Scheme 1, 3,3′,5,5′-tetrabromo-4,4′-dihexyl-2,2′-bithiophene (**1)** was synthesized in good yield via a base-catalyzed halogen dance (BCHD) reaction as described in the literature.^27^ Lithiation of **1** with two equivalent *n*-BuLi and the following addition of stoichiometric trimethylsilyl chloride (TMS-Cl) led to the formation of the precursor **Br**_**2**_**-2TC6-TMS**_**2**_**(2)**. In our expectation, treatment of **1** with stoichiometrically controlled *n*-BuLi would selectively remove α-site bromide and the following substitution with trimethylsilyl affords **2**. However, based on TLC and ^1^H NMR studies, we found that the Li–Br exchange on the β-site occurred simultaneously in minor selectivity even though addition of *n*-BuLi was carefully controlled, and the resultant β-selective side product showed closed polarity to **2** with an *R*_f_ value over 0.9 in hexane, enhancing the difficulty for column chromatographic purification. Therefore, we decided to use this crude product **2** directly in the subsequent reactions and attempted to isolate the afterward intermediates.

**Figure 2:**
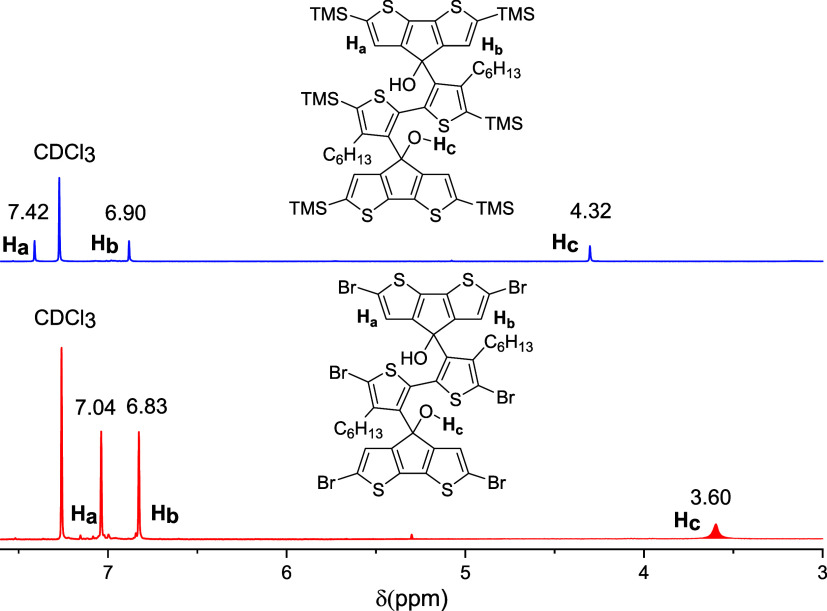
^1^H NMR spectra of **6b** and **8**.

**Scheme 2:**
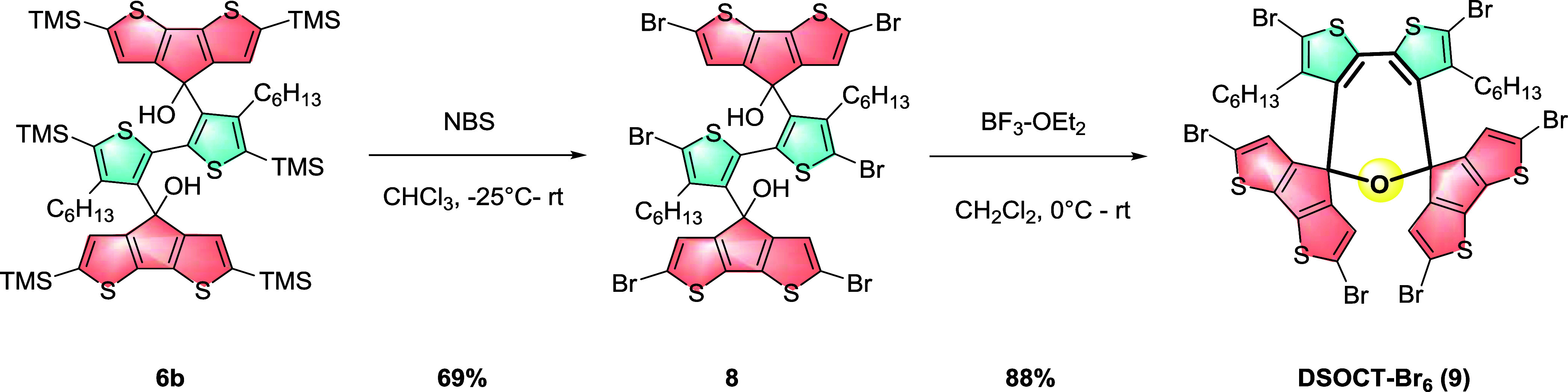
Synthetic Approach to **DSOCT-Br**_**6**_**(9)**

In the effort to prepare precursors for the synthesis of spiro compounds, two pathways were investigated. In the first one, treatment of **2** with *n*-BuLi and ketone **3** may afford **6a** and **6b**. Similarly, in another path, **2** is converted into ketone **4**, which then reacts with lithiated **5** to afford molecules **7a** and **7b**. According to pioneering works and our previous synthetic experience,^16,27^ we proposed that fast addition of two equivalent *n*-BuLi (2 equiv) in one step would favor rapid Li–Br exchange on both thiophene β-sites of a bithiophene structure and generate two nucleophilic centers. Therefore, diol molecules **6b** and **7b** would be the major selected products after nucleophilic addition with **3**. If slow dropwise addition of two equivalent *n*-BuLi is introduced step by step (1 equiv +1 equiv), the subsequent nucleophilic attack would first occur on one nucleophilic center, followed by a second-step Li–Br exchange of the residue bromine. In this situation, formation of **6a** and **7a** would be more favored. However, in practice, we found that only the diol intermediate **6b** with three bulky groups was the major selective product whose structure was confirmed by ^1^H NMR and MADLI-TOF-MS study, regardless of whether *n*-BuLi was added by fast addition or by step-by-step dropwise addition. Interestingly, the obtained ^1^H NMR spectrum of **6b** shows two environmental β-site thiophene-based protons at 7.42 ppm (H_a_) and 6.90 ppm (H_b_), respectively, regarding the 4H-cyclopenta[2,1-b:3,4-b’]dithiophene (CPDT) moiety, suggesting that a steric effect within **6b** causes a large torsion angle between 2,2-bithiophene and CPDT conformations (Figure 2). We ultimately confirmed the formation of **6b** by identifying the integration ratio of 1:1:1 for H_a_, H_b_, and H_c_ (−OH group, 4.32 ppm), and a signal peak at 1150.55 Da accounting for the presence of **6b** on MALDI-TOF-MS spectrum (Figure S4). **7a** was collected in only 10% best yield but demonstrated poor experimental reproducibility (Scheme 2). The presence of **7a** was confirmed by MALDI-TOF-MS for a target molecular weight of 813.84 Da (Figure S20), but no molecular signals for either **6a** or **7b** were detected by any spectroscopic methods. We proposed this selectivity is subject to the orientation of the hexyl group. When the hexyl group was on the β-position of the ketone site, the plausible steric hindrance of **4** prohibited the nucleophilic attack by lithiated **5** to the carbonyl group and hence caused low conversion of the ketone.^27^ It is remarkable that when the hexyl group was on the β-site of the bithiophene ring and was adjacent to the aromatic C–Br bond, the steric hindrance of **2** seemed negligible and the electron-donating character of the hexyl group even facilitated the rapid Li–Br exchange on both symmetric sites so that it was kinetically hard to obtain a single activated nucleophilic center by stoichiometric control. Additionally, other aromatic ketones such as 9-fluorenone and 4,5-diazafluoren-9-one were employed as substrates to enrich dispiro building blocks similar to compound **9** (Schemes S2 and S3). Regrettably, both six-membered aromatic ketones failed to give the corresponding tertiary diols, which can be reasoned by the enhanced steric hindrance of the electrophilic center when the thiophene moiety is substituted with benzene and pyridine rings. Besides, we found that 4,5-diazafluoren-9-one exhibited poor solubility in THF at −78 °C, leading to almost 100% recovery of this substrate. 9-Fluorenone was recovered with approximately 90% recovery rate. Based on several attempts (Table S1), we ultimately chose **6b**, the tertiary diol, with the best yield of 90% and convincible experimental reproducibility as a key precursor for construction of the 2,7-dihydroxepine core bearing spiro-conformation.

**Figure 3:**
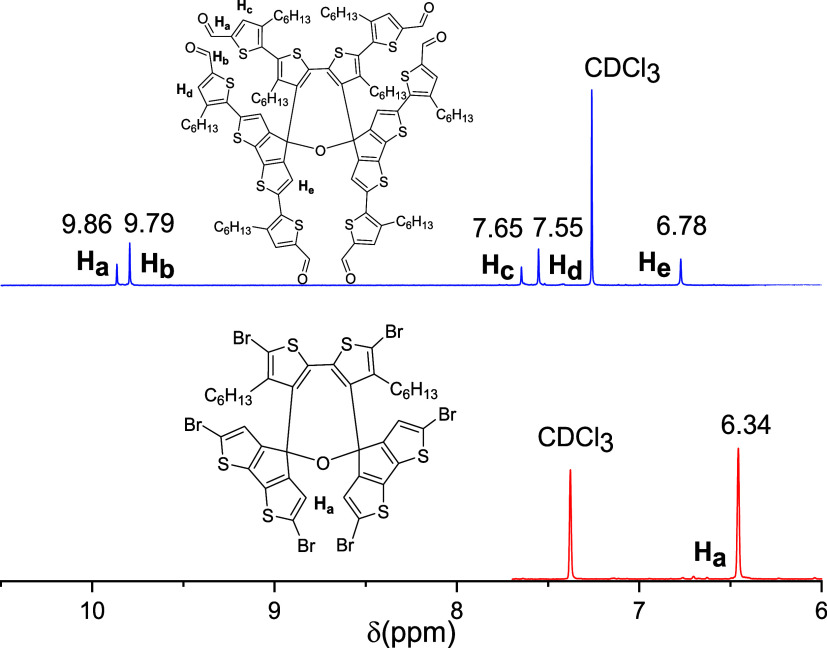
^1^H NMR spectra of **9** and **10**.

With **6b** in hand, the necessity of bromination prior to dehydration is to avoid deprotection of the trimethylsilyl group and subsequent α-site polymerization under the catalysis of Lewis acid. In addition, bromination also provides an alternative possibility for further deviation via coupling reactions. This step was complete upon dropwise addition of NBS in DMF solution and achieved 69% isolated yield. Notably, the two β-site protons on the CPDT conformation of **8** are downshifted and become closer to each other, as observed in the ^1^H NMR spectra (Figure 2). This observation could be explained by the reduced conformational torsion angle after the substitution of trimethylsilyl groups with the less-hindered bromine atoms. To minimize the side selectivity toward intermolecular dehydration, dropwise addition of **8** in dilute dichloromethane solution into dilute BF_3_–OEt_2_ solution gave rise to 2,7-dihydrooxepine-cored spirodithiophene molecule **9** (Scheme 2) with a best yield of 88%. The ^1^H NMR spectra (Figure 3) indicate that **9** is conformationally symmetric, as only one group of aromatic protons are present at 6.34 ppm, which is different from the spectra of **6b** and **8** with two groups of aromatic protons. Except aromatic and aliphatic protons, no proton signals for the −OH group are observed.

The MALDI-TOF-MS spectrum (Figure S10) with a peak at 1167.36 Da accounts for the presence of the target product. The peaks around 1090.46 Da are identified as penta-brominated species. To explain the role of BF_3_–OEt_2_ in the cyclization, our proposed mechanism (Scheme S1) suggests that the intramolecular dehydration starts from *O*-borylation of one −OH group (blue highlighted), which results in the rapid generation of the oxonium ion. Meanwhile, the strong electron-withdrawing character of one fluorine atom on BF_3_ tends to bond with another hydrogen on the unreacted −OH group (pink highlighted), leading to B–F bond cleavage and elimination of one HF molecule. The *O*-borylation enables the formed −OBF_2_ to be severed as a good leaving group, which can be easily removed intramolecularly via the S_N_2 mechanism, and a dihyrooxepine core bearing spiro-conformation is readily formed. Subsequent deprotonation of oxonium ion afforded 2,7-dihydrooxepine scaffold **9** and difluoro boric acid (**BF**_**2**_**OH**) as the side product.

**Figure 4:**
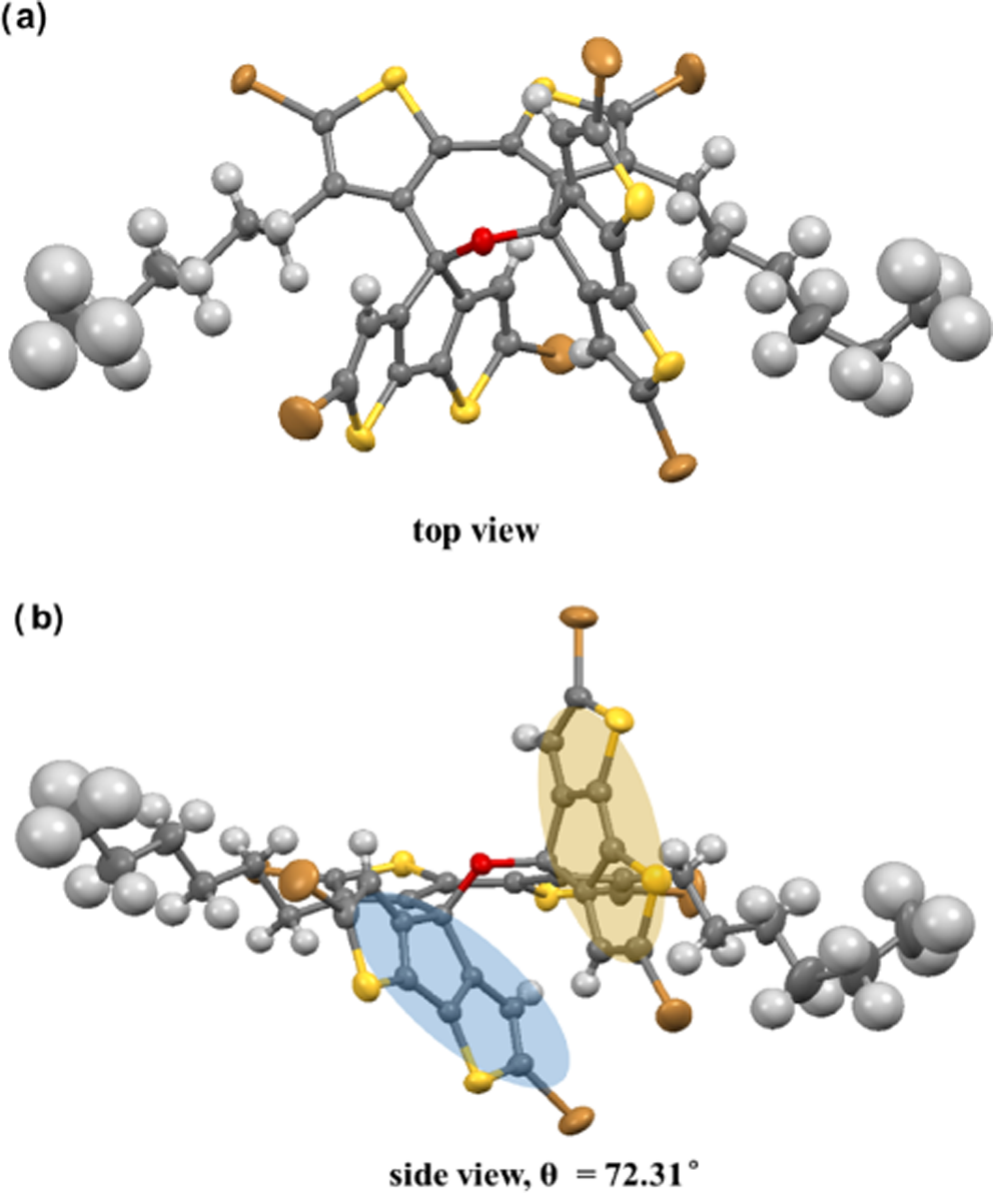
Single-crystal X-ray structure: (a) top view and (b) side view of **DSOCT-Br**_**6**_**(9)**; CCDC deposition number 2350287.

The single crystal of **9** was prepared by liquid–liquid slow diffusion in a THF-methanol dual-solvent system, in which THF served as a good solvent and methanol served as a poor solvent. As shown in Figure 4, single-crystal X-ray diffraction (SC-XRD) analysis confirms the formation of a dihydrooxepine core along with dispiro-conformation on its 2,7-site. As expected, the seven-membered ring is not coplanar but is arranged as a boat conformation, and the mean dihedral angle between C_2_–O-C_7_ and C_3_–C_4_-C_5_–C_6_ fragments is measured to be 71.45°. Besides, it is obviously seen that both spiro-conformations are nonorthogonal. This is subject to the conformational rotation of two CPDT groups through the sp^3^ carbon because of the steric hindrance with their neighboring hexyl groups. Thus, the two CPDT groups are twisted around each other with a dihedral angle of 72.31°. Upon proof of the desired structure, the other residual peaks in the MALDI-TOF-MS spectrum of **9** can be explained as fragment signals rather than impurities.

**Scheme 3:**
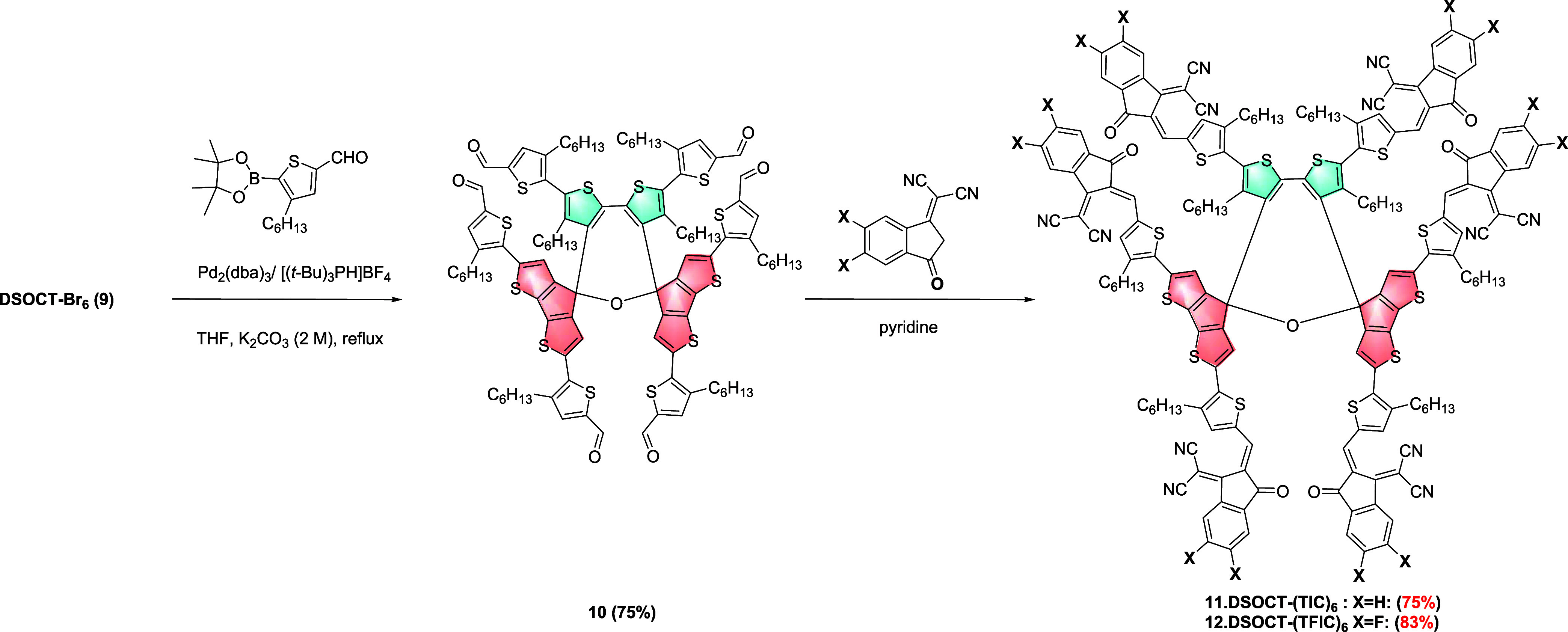
Peripheral Functionalization of **9** into **11** and **12**

**DSOCT-Br**_**6**_**(9)** was further derivatized in two steps. First, the Suzuki coupling reaction with 4-hexyl-5-(4,4,5,5-tetramethyl-1,3,2-dioxaborolan-2-yl)thiophene-2-carbaldehyde (**B-TCHO**) afforded **10** in a yield of 75%. Second, the sequent Knoevenagel condensation with 2-(3-oxo-2,3-dihydro-1H-inden-1-ylidene)malononitrile (IC) and 2-(5,6-difluoro-3-oxo2,3-dihydro-1H-inden-1-ylidene)malononitrile (FIC) using pyridine as a base catalyst afforded A–D–A-type cyclic conjugated compounds **11** and **12** in a yield of 75 and 83%, respectively (Scheme 3). In the first derivation step, we were able to ulteriorly confirm the successful generation of the 2,7-dihydrooxepine-spirodithiophene framework by spectroscopic analysis. For instance, the ^1^H NMR spectrum of **10** (Figure 3) obviously shows two different environments of protons with an integrated ratio of 1:2 for aldehyde groups (H_a_ and H_b_), π-bridge thiophen units (H_c_ and H_d_), and hexyl groups, which matches the spatial orientation of all groups within the molecular framework.

In addition, the exact mass of 1864.12 Da found in the MALDI-TOF-MS spectrum is consistent with the structure of **10**. Based on a comprehensive study, it is certain that we have obtained the key conformation bearing both dihydrooxepine and spirodithiophene building blocks.

**Figure 5:**
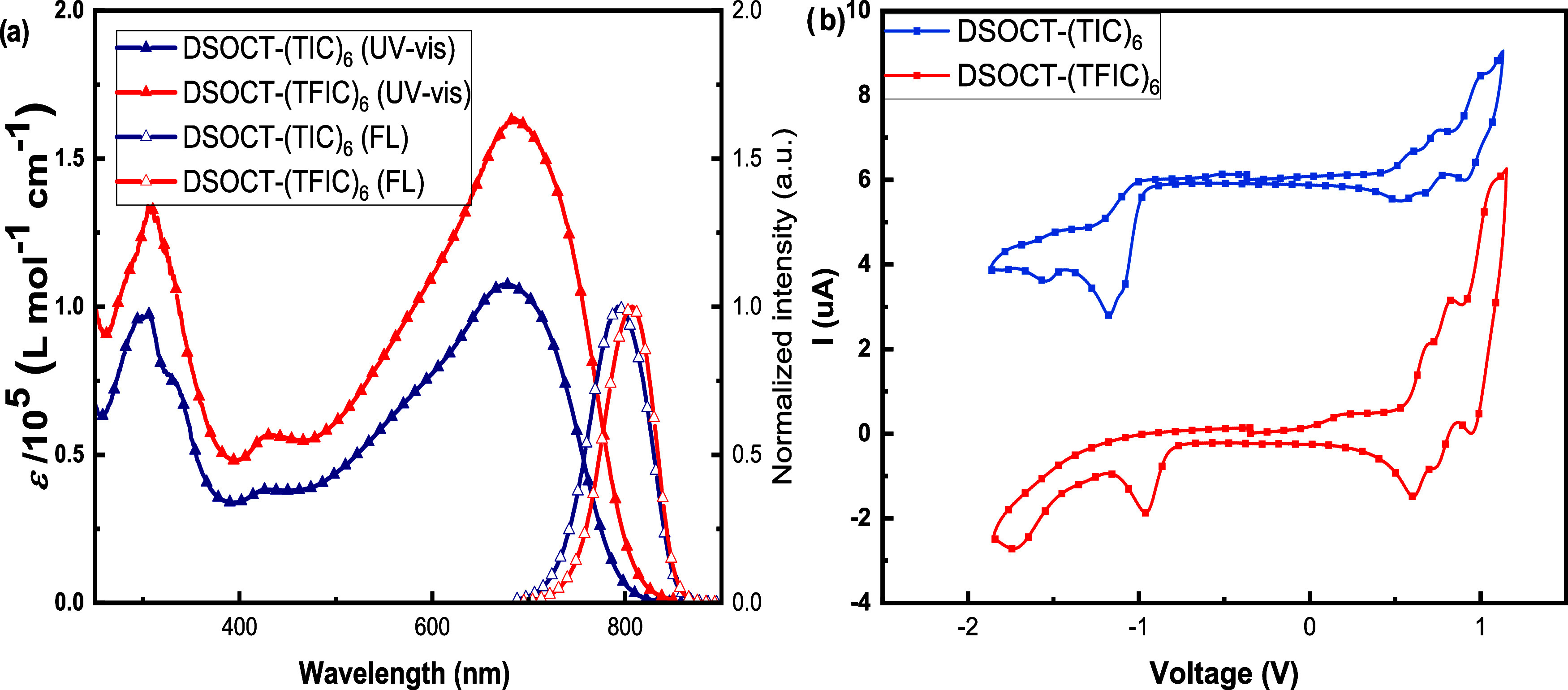
(a) UV–vis absorption and fluorescence spectra of **DSOCT-(TIC)**_**6**_ and **DSOCT-(TFIC)**_**6**_ in CHCl_3_ solution. (b) Cyclic voltammetry of **DSOCT-(TIC)**_**6**_ and **DSOCT-(TFIC)**_**6**_ in dichloromethane solution using TBAPF_6_ (0.1 M) as the supporting electrolyte and at a scan rate of 100 mV.

As shown in Figure 5, compounds **DSOCT-(TIC)**_**6**_ (**11**) and **DSOCT-(TFIC)**_**6**_ (**12**) showed a main absorption band over 500–800 nm. Compared to our previous results for linear analogues **CPDT-(TIC)**_**2**_ and **CPDT-(TFIC)**_**2**_(10) the spiro compounds present larger molar absorptivity (151,100 vs 102,000 L mol^–^ cm^–1^ and 215,400 vs 117,600 L mol^–1^ cm^–1^, respectively) (Table S2).

The band gaps for both molecules are determined by cyclic voltammetry (CV) to be 1.46 and 1.40 eV, respectively, consistent with the optical data (Figure 5). Fluorination at the terminal site lowers the HOMO/LUMO level, as well as the band gap of **DSOCT-(TFIC)**_**6**_, facilitating easier charge excitation and separation in organic solar cells, and a lower open circuit voltage (*V*_oc_) is observed.^28−30^ Additionally, fluorination at the terminal site presumably enhances the intermolecular π–π stacking, facilitating efficient carrier transportation,^28−30^ which is beneficial for a higher current density and fill factor of solar cells.^28,29^ Therefore, an overall improved power conversion efficiency (PCE) of fluorinated compound-based solar cells could be observed. In our devices investigation, the PCEs for **PM6**: **DSOCT-(TFIC)**_**6**_-based organic solar cells demonstrated better performance than those for **PM6: DSOCT-(TIC)**_**6**_-based devices as a result of the higher current density and fill factor, which is consistent with our expectation (Table S3).

**Figure 6:**
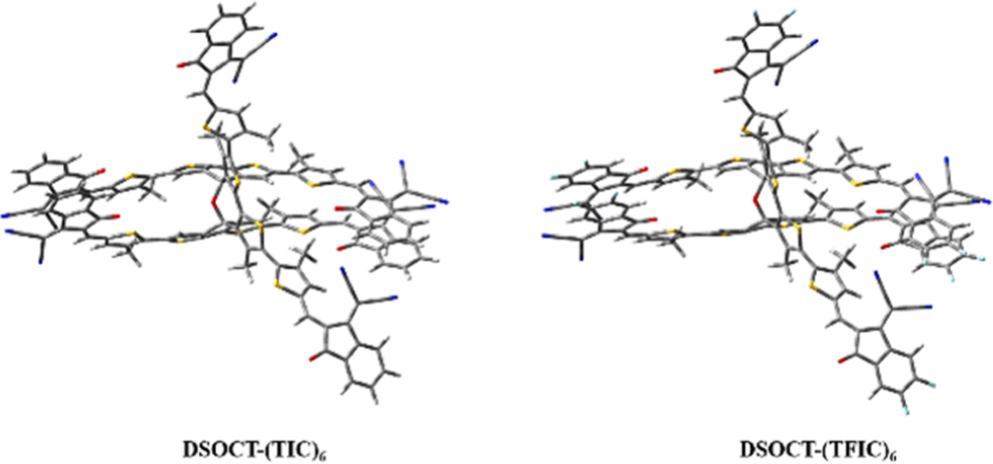
Optimized conformations of DSOCT-based molecules based on TD-DFT calculation.

To further understand the structure–property relationship of the two synthesized molecules, theoretical calculation was performed. To simplify the calculation, all hexyl groups on the DSOCT core were replaced by the methyl group (Figure 6). Based on molecular orbital simulation, both **DSOCT-(TIC)**_**6**_ and **DSOCT-(TFIC)**_**6**_ demonstrate dispiro structures with degenerate HOMO and LUMO orbital sets (Figure S23). One can see the π orbital electron density nature of these HOMO and LUMO orbitals. The calculated energy gap between the HOMO and LUMO orbitals of the gas-phase **DSOCT-(TIC)**_**6**_ is 1.96 eV. With the fluorination, both the HOMO and LUMO orbitals are stabilized while the LUMO is a bit more stable. Comparing the two optimized structures, the bond length, bond angle, and dihedral angle have little difference. The distance between the oxygen atom in the acceptor and the sulfur atom on the π bridge to form S···O intramolecular interactions is 2.70 Å. The TD-DFT modeling results show that **DSOCT-(TIC)**_**6**_ has strong absorption peaks at 686.83 nm, while the fluorinated molecule **DSOCT-(TFIC)**_**6**_ has a strong absorption peak at 703.05 nm (Figure S21). This is consistent with a previous study that shows a red shift upon the fluorination. Comprehensively, the two dispiro-based acceptor molecules **DSOCT-(TIC)**_**2**_ and **DSOCT-(TFIC)**_**2**_ demonstrated stronger absorptivity and a moderate band gap in comparison with the recently reported “star molecules” such as ITIC derivatives^28,31−33^ and Y-series.^29,34−36^ The three-armed π-systems serve as a better chromophore for light harvesting for a solar cell compared to the recently reported two-armed π-systems. A possible explanation for the low PCEs of the dispiro-molecule-based solar cells is the large torsion angles among the three π-systems. This would lead to conformational distortion of molecules in a blend film, resulting in significant “trap-assist” nonradiative decay of charge carriers.^37−39^ To improve the device efficiency through molecular design, it is essential to enhance the coplanarity of the entire structure.^40,41^ For instance, introduction of intramolecular noncovalent “conformational locks” such as S···O, S···F, S···N interactions would alleviate rotation of single bonds;^42,43^ alternatively, using the “fusing strategy” to construct conjugated fused-ladder type oligomers would also improve the coplanarity of the molecular skeleton.^31−36^

## Conclusions

In summary, our recent work has demonstrated a novel synthetic approach for a 2,7-dihydrooxepine core bearing a dispiro-conjugated framework. Their structural formation is based on a key step for which a major selectivity toward a diol intermediate is more preferred. Such selectivity is attributed to the enhanced nucleophilicity of the aromatic anion by the electron-donating character of adjacent alkyl groups. The mechanistic study shows that a fast proton transfer from one −OH group to another is a key step that facilitates dissociation of HF and fluoroboric acid. Peripheral functionalization of the core at terminal sites gives rise to the corresponding organic semiconductor materials with strong light absorption and fluorescence. Their optical band gap and electrochemical band gap are consistent with computational studies. Fluorination on terminal sites not only reduces the energy level and band gap of **DSOCT-(TFIC)**_**6**_ but also enhanced the current density and PCEs of the **PM6: DSOCT-(TFIC)**_**6**_-based organic solar cells. To further optimize the solar cell performance, structural modifications such as introduction of a noncovalent “conformational lock” or using the “fusing strategy” to improve molecular coplanarity, which will reduce nonradiative decay of the charge carriers in the blend film, may be considered.

## Experimental Section

### General Methods

All chemicals and solvents were reagent grade. The dry solvent was collected from PURESULV Solvent Purification system PS-MD-5ON7 (Innovative Technology). Chromatography was performed on silica gel 60 (particle size = 300–400 mesh) and TLC was performed on an aluminum substrate coated with silica gel 60F524 (Merck, layer thickness = 0.2 mm). Nuclear magnetic resonance (NMR) spectra were obtained using TMS as an internal reference in Bruker Ascend 400 Hz in deuterated chloroform (CDCl_3_) and dichloromethane (DCM), and spectra were referenced to the deuterated solvent peak at 7.26 and 77.16 ppm or 5.3 and 53.52 for proton and carbon NMR, respectively; all peaks were labeled and integrated accordingly. MALDI-TOF-MS spectra were obtained using Bruker Auto Bending Speed LRF with *trans*-2-[3-(4-*tert*-butylphenyl)-2-methyl-2-propenyl]malononitrile (DCTB) as a matrix substrate. Single-crystal data collection was performed on a Bruker D8 VENTURE Photon II diffractometer with graphite monochromated Mo Kα radiation (λ = 0.71073 Å) at room temperature, operating at 50 kV and 30 mA. Compounds **3**, **4**, and **5** were synthesized previously according to the literature.^16,27^

### Synthesis

#### 3,3′,5,5′-Tetrabromo-4,4′-dihexyl-2,2′-bithiophene **(1)**

To a solution of diisopropylamine (13 mL, 92.55 mmol, 1.5 equiv) in freshly dried THF (100 mL) was carefully added *n*-BuLi (2.5 M in hexane, 74.04 mmol, 30 mL, 1.2 equiv) at −78 °C. After stirring for 20 min, 2,5-dibromo-3-hexyl-thiophene (20 g, 61.7 mmol, 1 equiv) in dried THF (50 mL) was added dropwise to the *in situ* generated LDA solution over 30 min and was further stirred for 1 h. Anhydride CuCl_2_ (8.3 g, 61.7 mmol, 1 equiv) was then added in one portion, forming a dark blue solution, which was slowly warmed to rt and stirred for 19 h. The solution was diluted with PE and filtered through silica gel to give a clear yellow solution, which was concentrated to afford the product as yellow oil without further purification (19.6 g, 91% yield). ^1^H NMR (600 MHz, CDCl_3_) δ [ppm] = 2,66 (t, *J* = 8 Hz, 4H), 1.57–1.54 (m, 4H), 1.45–1.32 (m, 12H), 0.92–0.84 (t, *J* = 6.7 Hz, 6H); ^13^C{^1^H} NMR (151 MHz, CDCl_3_) δ [ppm] = 141.5, 128.6, 114.6, 111.1, 31.6, 30.4, 29.1, 28.6, 22.6, 14.1.

#### (3,3′-Dibromo-4,4′-dihexyl-[2,2′-bithiophene]-5,5′-diyl)bis(trimethylsilane) **(2)**

To a solution of **2** (18.55 g, 28.5 mmol, 1 equiv) in dried THF (50 mL) was added *n*-BuLi (2.5 M, 57.1 mmol, 22.8 mL, 2 equiv) over 30 min at −78 °C and was stirred for 20 min. TMS-Cl (7.6 mL, 59.9 mmol, 2.1 equiv) in THF (20 mL) was added quickly and the mixture was slowly warmed to rt and stirred overnight. The reaction mixture was quenched with saturated NH_4_Cl solution and the organic phase was separated and concentrated. The vicious mixture was diluted with PE, washed with water, dried with Na_2_SO_4_, and concentrated. The brown oil was further filtered through silica gel using PE as an eluent to afford the crude product mixture as a yellow viscous oil (16.55 g). This oil was directly used for the next step without any further purifications. ^1^H NMR (600 MHz, CDCl_3_) δ 2.67 (t, *J* = 8 Hz, 4H), 1.61–1.54 (m, 4H), 1.47–1.39 (m, 4H), 1.34–1.33 (m, 8H), 0.90 (t, *J* = 6 Hz, 6H), 0.36 (s, 18H); ^13^C{^1^H} NMR (151 MHz, CDCl_3_) δ [ppm] = 148.3, 135.2, 133.9, 116.5, 31.7, 31.4, 30.6, 29.6, 22.5, 13.9.

#### 4,4′-(4,4′-Dihexyl-5,5′-bis(trimethylsilyl)- [2,2′-bithiophene]-3,3′-diyl) bis (2,6-bis(trimethylsilyl)-4H-cyclopenta[2,1-b:3,4-b’]dithiophen-4-ol) **(6b)**

To a solution of freshly prepared **2** (7g, 10.99 mmol, 1 equiv) in dried THF (100 mL) was added *n*-BuLi (2.5 M, 22.01 mmol, 9 mL, 2 equiv) at −78 °C for 20 min. After stirring of this clear yellow solution for an additional 20 min at this temperature, a solution of **3** (5.5 g, 16.48 mmol, 1.5 equiv) in dried THF (30 mL) was added dropwise to the mixture. The orange suspension was then slowly warmed to ambient temperature and was further stirred overnight. After quenching with a saturated NH_4_Cl solution, the organic phase was separated and concentrated to a dark brown vicious oil. This oil was diluted with DCM (50 mL), washed with water (100 mL), dried with Na_2_SO_4_, and concentrated. Further purification of the crude oil using 15–20% DCM/PE on silica gel afforded the product as a light-yellow powder (7.1 g, 61% yield). ^1^H NMR (600 MHz, CDCl_3_) δ [ppm] = 7.41 (s, 2H), 6.88 (s, 2H), 4.29 (s, 2H), 2.01 (t, *J* = 4.8 Hz, 2H), 1.39 (t, *J* = 4.8 Hz, 2H), 1.19–1.08 (m, 4H), 1.00–0.85 (m, 8H), 0.82 (t, *J* = 7.4 Hz, 6H), 0.81–0.76 (m, 2H), 0.76–0.67 (m, 2H), 0.32 (s, 18H), 0.28 (s, 18H), 0.25 (s, 18H); ^13^C{^1^H} NMR (151 MHz, CDCl_3_) δ [ppm] = 159.5, 157.3, 149.0, 143.4, 143.0, 142.8, 142.5, 139.9, 138.8, 135.0, 129.6, 128.8, 78.7, 32.1, 31.9, 30.5, 30.3, 22.6, 14.1; MS (MALDI–TOF): calcd for C_56_H_86_O_2_S_6_Si_6_*m*/*z* = 1150.36 [M]^+^, found: *m*/*z* = 1150.55 [M]^+^.

#### 4,4′-(5,5′-Dibromo-4,4′-dihexyl-[2,2′-bithiophene]-3,3′-diyl) bis (2,6-dibromo-4H-cyclopenta[2,1-b:3,4-b’]dithiophen-4-ol) **(8)**

To a solution of **6b** (6.5 g, 5.64 mmol, 1 equiv) in a mixed solvent of dried chloroform (50 mL) and DMF (10 mL) was added dropwise a solution of NBS (6.52 g, 36.7 mmol, 6.5 equiv) in dried DMF (10 mL) at −25 °C. To this, one drop of acetic acid was added and the reaction mixture became a brown solution. The solution was slowly warmed to ambient temperature and stirred overnight in dark. After completion, the resultant mixture was diluted with DCM (150 mL) and washed several times with water to remove DMF. The combined organic phase was dried and concentrated to afford a dark oil. Further purification of the dark oil via chromatography by eluting with 25% DCM/PE afforded the product as a light brown solid (4.7 g, 69% yield). ^**1**^H NMR (600 MHz, CDCl_3_) δ [ppm] = 7.04 (s, 2H), 6.83 (s, 2H), 3.61 (s, 2H), 1.99 (td, *J* = 13.1, 4.8 Hz, 2H), 1.67 (td, *J* = 13.0, 4.5 Hz, 2H), 1.24–1.17 (m, 4H), 1.11–1.02 (m, 4H), 0.98–0.90 (m, 4H), 0.86 (t, *J* = 7.4 Hz, 6H), 0.84–0.79 (m, 2H), 0.73–0.63 (m, 2H);^13^C{^1^H} NMR δ [ppm] = (151 MHz, CDCl_3_), 154.3, 152.6, 140.1, 137.9, 137.6, 135.3, 133.7, 125.2, 125.0, 113.8, 113.5, 112.4, 80.4, 31.7, 30.2, 29.2, 28.7, 22.6, 14.2; MS (MALDI–TOF): calcd for C_38_H_33_Br_6_O_2_S_6_*m*/*z* = 1185.58 [M]^+^, found: *m*/*z* = 1185.37 [M]^+^.

#### 2,2′,2″,6,6″,8′-Hexabromo-3′,7′-dihexyldispiro[cyclopenta[2,1-b:3,4-b’]dithiophene-4,4′-dithieno[3,2-c:2′,3′-*e*]oxepine-6′,4″-cyclopenta[2,1-b:3,4-b’]dithiophene] **(9)**

A solution of **8** (4 g, 3.35 mmol, 1 equiv) in DCM (200 mL) was dropwise with BF_3_–OEt_2_ (2.38 g, 16.8 mmol, 5 equiv) over 30 min at ambient temperature with vigorous stirring; a dark green solution was formed. This solution was further stirred at room temperature overnight, followed by quenching with a saturated NaHCO_3_ solution. The organic phase was dried with Na_2_SO_4_ and concentrated. The resulting dark solid was purified via chromatography using PE as an eluent to afford the product as an off-white powder (3.5 g, 88% yield). ^1^H NMR (600 MHz, CDCl_3_) δ [ppm] = 6.34 (s, 4H), 1.87 (t, *J* = 8.4 Hz, 4H), 1.23–1.13 (m, 4H), 1.08–1.00 (m, 4H), 0.90–0.86 (m, 4H), 0.83 (t, *J* = 7.4 Hz, 6H), 0.82–0.76 (m, 4H); ^13^C{^1^H} NMR (151 MHz, CDCl_3_) δ [ppm] = 150.9, 141.1, 138.6, 137.6, 135.1, 112.4, 111.0, 84.9, 31.7, 29.7, 29.5, 28.8, 22.6, 14.1; MS (MALDI–TOF): calcd for C_38_H_30_Br_6_OS_6_*m*/*z* = 1167.57 [M]^+^, found: *m*/*z* = 1167.36 [M]^+^.

### [DSOCT-(TCHO)_6_]

A solution containing **9** (1.73 g, 1.47 mmol, 1 equiv), PH(*t*-Bu_3_)BF_4_ (51.2 mg, 0.176 mmol, 0.12 equiv), and 4-hexyl-5-(4,4,5,5-tetramethyl-1,3,2-dioxaborolan-2-yl)thiophene-2-carbaldehyde (B-TCHO) (6 g, 18.6 mmol, 13 equiv) in THF (30 mL) was mixed with K_2_CO_3_ solution (2M, 20 mL). The mixture was stirred and purged with argon for 20 min. Pd(PPh_3_)_4_ (60 mg, 0.09 mmol, 0.06 equiv) was then quickly added to the mixture and the resulting yellow-brown solution was further purged for an additional 10 min. After refluxing for 48 h, the dark mixture was separated. The organic phase was diluted with DCM and washed with water (200 mL). When concentrated under reduced pressure, the crude product was purified via chromatography using 50%DCM/PE as the eluent to afford an orange powder. Further purification by gel permeant chromatography afforded the pure product as orange crystals (2.06 g, 75% yield). ^1^H NMR (400 MHz, CDCl_3_) δ [ppm] = 9.86 (s, 2H), 9.79 (s, 4H), 7.65 (s, 2H), 7.55 (s, 4H), 6.77 (s, 4H), 2.77 (t, *J* = 7.8 Hz, 8H), 2.60 (t, *J* = 7.6 Hz, 4H), 1.96–1.92 (m, 4H), 1.70–1.58 (m, 12H), 1.40–1.35 (m, 12H), 1.31–1.19 (m, 36H), 0.97–0.92 (m, 4H), 0.86 (t, *J* = 7.2 Hz, 12H), 0.79 (t, *J* = 6.6 Hz, 6H), 0.64 (t, *J* = 7.2 Hz, 6H); ^13^C{^1^H} NMR (100 MHz, CDCl_3_) δ [ppm] = 182.8, 182.2, 153.8, 144.6, 143.1, 142.4, 140.9, 140.3, 140.2, 140.2, 139.0, 138.9, 138.0, 137.1, 136.9, 136.8, 128.7, 124.2, 84.7, 31.6, 31.6, 31.4, 30.4, 30.1, 29.8, 29.7, 29.2, 29.1, 28.9, 27.1, 24.9, 24.6, 22.6, 22.5, 22.4, 14.1; MS (MALDI–TOF): calcd for C_104_H_120_O_7_S_12_*m*/*z* = 1864.57 [M]^+^, found: *m*/*z* = 1864.12 [M]^+^.

### [DSOCT-(TIC)_6_]

A solution of DSOCT-(TCHO)_6_ (500 mg, 0.27 mmol, 1 equiv) and 2-(3-oxo-2,3-dihydro-1H-inden-1-ylidene)malononitrile (410 mg, 2.16 mmol, 8 equiv) in chloroform was stirred at 50 °C for 15 min, and 0.1 mL of pyridine was added. The solution gradually turned into a dark blue color and was refluxed overnight. After being cooled to room temperature, the solution was concentrated and the resultant crude product was purified via chromatography using 50% DCM/PE as the eluent to give a dark blue powder. Further purification using gel permeation chromatography and subsequent recrystallization in methanol afforded the product as dark blue crystals (590 mg, 75% yield). ^1^H NMR (400 MHz, CDCl_3_) δ [ppm] = 8.79–8.65 (m, 6H), 8.65–8.54 (m, 6H), 7.85–7.66 (m, 18H), 7.63 (s, 6H), 7.03 (s, 4H), 2.82 (t, *J* = 8.4 Hz, 8H), 2.70 (t, *J* = 7.7 Hz, 4H), 2.20–2.16 (m, 4H), 1.71–1.65 (m, 12H), 1.39–1.34 (m, 12H), 1.28–1.22 (m, 24H), 1.05–0.92 (m, 4H), 0.90–0.75 (m, 30H), 0.64 (t, *J* = 7.2 Hz, 6H); ^13^C{^1^H} NMR (100 MHz, CDCl_3_) δ [ppm] = 187.9, 160.1, 148.5, 147.8, 145.7, 144.3, 141.9, 141.0, 139.9, 138.0, 137.1, 136.8, 135.2, 134.5, 125.2, 123.7, 122.5, 117.4, 114.5, 114.2, 69.3, 31.6, 30.6, 29.9, 29.7, 29.4, 29.1, 22.6, 22.6, 22.5, 14.1,; MS (MALDI–TOF): calcd for C_176_H_143_N_12_O_7_S_12_*m*/*z* = 2919.79 [M + H]^+^, found: *m*/*z* = 2919.86 [M + H]^+^.

### [DSOCT-(TFIC)_6_]

A solution of DSOCT-(TCHO)_6_ (500 mg, 0.27 mmol, 1 equiv) and 2-(5,6-difluoro-3-oxo-2,3-dihydro-1H-inden-1-ylidene)malononitrile (496 mg, 2.16 mmol, 8 equiv) in chloroform was stirred at 50 °C for 15 min, and 0.1 mL of pyridine was added dropwise. The solution gradually turned into dark blue color and was refluxed overnight. After cooling to room temperature, the solution was concentrated and the resulting crude product was purified via chromatography using 50% DCM/PE as eluent to give dark blue powders. Further purification using gel permeation chromatography and subsequent recrystallization in methanol afforded the product as dark blue crystals (700 mg, 83% yield). ^1^H NMR (400 MHz, CDCl_3_) δ [ppm] = 8.72 (s, 6H), 8.59–8.44 (m, 6H), 7.71–7.64 (m, 6H), 7.61–7.52 (m, 6H), 7.05 (s, 4H), 2.83 (t, *J* = 7.8 Hz, 8H), 2.70 (t, *J* = 7.9 Hz, 4H), 2.21–2.16 (m, 4H), 1.75–1.63 (m, 12H), 1.38–1.33 (m, 12H), 1.30–1.20 (m, 24H), 1.04–0.94 (m, 4H), 0.92–0.74 (m, 30H), 0.66 (t, *J* = 7.3 Hz, 6H); ^13^C NMR (100 MHz, CDCl_3_) δ [ppm] = 185.8, 158.00, 153.3, 149.1, 148.4, 146.3, 144.7, 141.4, 138.0, 137.6, 137.2, 136.6, 134.5, 125.3, 121.7, 115.1, 114.0, 107.7, 70.2, 31.6, 31.5, 30.5, 29.9, 29.7, 29.4, 29.1, 28.8, 22.6, 22.5, 14.1; MS (MALDI–TOF): calcd for C_176_H_132_F_12_N_12_O_7_S_12_*m*/*z* = 3135.67 [M + H]^+^, found: *m*/*z* = 3135.52 [M + H]^+^.

## Supporting Information Available

Additional experimental details, materials, methods, characterization data (UV–vis, fluorescence, 1^H^, 13^C^, and mass spectra, cyclic voltammograms), and solar cell performance data (PDF)

## References

[ref1] Hamada H.; Itabashi Y.; Shang R.; Nakamura E. Axially Chiral Spiro-Conjugated Carbon-Bridged p-Phenylenevinylene Congeners: Synthetic Design and Materials Properties. J. Am. Chem. Soc. 2020, 142 (4), 2059–2067. 10.1021/jacs.9b13019. 31922417

[ref2] Hamada H.; Nakamuro T.; Yamashita K.; Yanagisawa H.; Nureki O.; Kikkawa M.; Harano K.; Shang R.; Nakamura E. Spiro-Conjugated Carbon/Heteroatom-Bridged p-Phenylenevinylenes: Synthesis, Properties, and Microcrystal Electron Crystallographic Analysis of Racemic Solid Solutions. Bull. Chem. Soc. Jpn. 2020, 93 (6), 776–782. 10.1246/bcsj.20200065.

[ref3] Takase K.; Noguchi K.; Nakano K. Circularly Polarized Luminescence from Chiral Spiro Molecules: Synthesis and Optical Properties of 10,10′-Spirobi(indeno[1,2-b][1]benzothiophene) Derivatives. Org. Lett. 2017, 19 (19), 5082–5085. 10.1021/acs.orglett.7b02337. 28926271

[ref4] Simmons H. E.; Fukunaga T. Spiroconjugation. J. Am. Chem. Soc. 1967, 89 (20), 5208–5215. 10.1021/ja00996a022.

[ref5] Qu Y.-K.; Zheng Q.; Fan J.; Liao L.-S.; Jiang Z.-Q. Spiro Compounds for Organic Light-Emitting Diodes. Acc. Mater. Res. 2021, 2 (12), 1261–1271. 10.1021/accountsmr.1c00208.

[ref6] Wössner J. S.; Esser B. Spiroconjugated Donor−σ–Acceptor Charge-Transfer Dyes: Effect of the π-Subsystems on the Optoelectronic Properties. J. Org. Chem. 2020, 85 (7), 5048–5057. 10.1021/acs.joc.0c00567. 32180403

[ref7] Pan J.; Wang L.; Chen W.; Sang S.; Sun H.; Wu B.; Hang X.-C.; Sun Z.; Huang W. Non-fullerene small molecule acceptors with three-dimensional thiophene/selenophene-annulated perylene diimides for efficient organic solar cells. J. Mater. Chem. C 2020, 8 (20), 6749–6755. 10.1039/D0TC00341G.

[ref8] Saragi T. P. I.; Spehr T.; Siebert A.; Fuhrmann-Lieker T.; Salbeck J. Spiro Compounds for Organic Optoelectronics. Chem. Rev. 2007, 107 (4), 1011–1065. 10.1021/cr0501341. 17381160

[ref9] Nguyen T. B.; Retailleau P. Direct access to thieno[3,4-b]thiophenes via elemental sulfur-promoted sulfurative tetramerization of acetophenones. Chem. Commun. 2022, 58 (96), 13333–13336. 10.1039/D2CC05539B. 36373658

[ref10] Nguyen T. B.; Retailleau P. DIPEA-Promoted Reaction of 2-Nitrochalcones with Elemental Sulfur: An Unusual Approach to 2-Benzoylbenzothiophenes. Org. Lett. 2017, 19 (18), 4858–4860. 10.1021/acs.orglett.7b02321. 28840729

[ref11] Nguyen T. B.; Retailleau P. Cooperative Activating Effect of Tertiary Amine/DMSO on Elemental Sulfur: Direct Access to Thioaurones from 2′-Nitrochalcones under Mild Conditions. Org. Lett. 2018, 20 (1), 186–189. 10.1021/acs.orglett.7b03547. 29219319

[ref12] dos Santos J. M.; Jagadamma L. K.; Cameron J.; Wiles A. A.; Wilson C.; Skabara P. J.; Samuel I. D. W.; Cooke G. New thiophene-based conjugated macrocycles for optoelectronic applications. J. Mater. Chem. C 2021, 9 (45), 16257–16271. 10.1039/D1TC02002A.

[ref13] Zhang C.; Zhu X. Thieno[3,4-b]thiophene-Based Novel Small-Molecule Optoelectronic Materials. Acc. Chem. Res. 2017, 50 (6), 1342–1350. 10.1021/acs.accounts.7b00050. 28375613

[ref14] Lin Y.; Li Y.; Zhan X. Small molecule semiconductors for high-efficiency organic photovoltaics. Chem. Soc. Rev. 2012, 41 (11), 4245–4272. 10.1039/c2cs15313k. 22453295

[ref15] Pozzi G.; Orlandi S.; Cavazzini M.; Minudri D.; Macor L.; Otero L.; Fungo F. Synthesis and Photovoltaic Applications of a 4,4′-Spirobi[cyclopenta[2,1-b;3,4-b′]dithiophene]-Bridged Donor/Acceptor Dye. Org. Lett. 2013, 15 (18), 4642–4645. 10.1021/ol402420w. 23984707

[ref16] Liu X.; Zhang Y.; Wu J.; Ma Y.; Lau K. K. T.; Fang J.; Ma C.-Q.; Lin Y. Simplified Synthetic Approach to Tetrabrominated Spiro-Cyclopentadithiophene and the Following Derivation to A-D-A Type Acceptor Molecules for Use in Polymer Solar Cells. J. Org. Chem. 2022, 87 (8), 5057–5064. 10.1021/acs.joc.1c02848. 35333523

[ref17] Zhang G.; Lin F. R.; Qi F.; Heumüller T.; Distler A.; Egelhaaf H.-J.; Li N.; Chow P. C. Y.; Brabec C. J.; Jen A. K. Y.; Yip H. L. Renewed Prospects for Organic Photovoltaics. Chem. Rev. 2022, 122 (18), 14180–14274. 10.1021/acs.chemrev.1c00955. 35929847

[ref18] Suzuki M.; Suzuki K.; Won T.; Yamada H. Impact of substituents on the performance of small-molecule semiconductors in organic photovoltaic devices via regulating morphology. J. Mater. Chem. C 2022, 10 (4), 1162–1195. 10.1039/D1TC04237H.

[ref19] Xie L.-H.; Hou X.-Y.; Tang C.; Hua Y.-R.; Wang R.-J.; Chen R.-F.; Fan Q.-L.; Wang L.-H.; Wei W.; Peng B.; Huang W. Novel H-Shaped Persistent Architecture Based on a Dispiro Building Block System. Org. Lett. 2006, 8, 1363–1366. 10.1021/ol060109x. 16562892

[ref20] Wu Y.; Zhang J.; Bo Z. Synthesis of Monodisperse Spiro-Bridged Ladder-Type Oligo-p-phenylenes. Org. Lett. 2007, 9, 4435–4438. 10.1021/ol7017533. 17894503

[ref21] Poriel C.; Rault-Berthelot J.; Barrière F.; Slawin A. M. Z. New Dispiro Compounds: Synthesis and Properties. Org. Lett. 2008, 10, 373–376. 10.1021/ol7026202. 18183993

[ref22] Kimura M.; Kuwano S.; Sawaki Y.; Fujikawa H.; Noda K.; Taga Y.; Takagi K. New 9-fluorene-type trispirocyclic compounds for thermally stable hole transport materials in OLEDs. J. Mater. Chem. 2005, 15 (24), 2393–2398. 10.1039/B502268A.

[ref23] Wan Z.; Yang J.; Xia J.; Shu H.; Yao X.; Luo J.; Jia C. A new strategy for constructing a dispiro-based dopant-free hole-transporting material: spatial configuration of spiro-bifluorene changes from a perpendicular to parallel arrangement. Chem. Sci. 2021, 12, 8548–8555. 10.1039/D1SC01416A. 34221336 PMC8221193

[ref24] Ishigaki Y.; Hayashi Y.; Sugawara K.; Shimajiri T.; Nojo W.; Katoono R.; Suzuki T. 9,10-Dihydrophenanthrene with Two Spiro(dibenzocycloheptatriene) Units: A Highly Strained Caged Hydrocarbon Exhibiting Reversible Electrochromic Behavior. Molecules 2017, 22, 1900 10.3390/molecules22111900. 29113057 PMC6150351

[ref25] Suzuki T.; Yamamoto R.; Higuchi H.; Hirota E.; Ohkita M.; Tsuji T. Electrochiroptical response of a hexaarylethane derivative with a helical π-skeleton: drastic UV–Vis and CD spectral changes upon electrolysis of 4′,5′-dibromodispiro[xanthene-9,9′(9′H,10′H)-phenanthrene-10′,9″-xanthene]. J. Chem. Soc., Perkin Trans. 2 2002, 2, 1937–1942. 10.1039/B204515J.

[ref26] Nishida J.-i.; Miyagawa T.; Yamashita Y. Novel Thiophene Oligomers Containing a Redox Active Hexaarylethane Unit. Org. Lett. 2004, 6, 2523–2526. 10.1021/ol049216m. 15255681

[ref27] Getmanenko Y. A.; Risko C.; Tongwa P.; Kim E.-G.; Li H.; Sandhu B.; Timofeeva T.; Brédas J.-L.; Marder S. R. Mono- and Dicarbonyl-Bridged Tricyclic Heterocyclic Acceptors: Synthesis and Electronic Properties. J. Org. Chem. 2011, 76, 2660–2671. 10.1021/jo102502u. 21391630

[ref28] Swick S. M.; Alzola J. M.; Sangwan V. K.; Amsterdam S. H.; Zhu W.; Jones L. O.; Powers-Riggs N.; Facchetti A.; Kohlstedt K. L.; Schatz G. C.; et al. Fluorinating π-Extended Molecular Acceptors Yields Highly Connected Crystal Structures and Low Reorganization Energies for Efficient Solar Cells. Adv. Energy Mater. 2020, 10 (23), 2000635 10.1002/aenm.202000635.

[ref29] Li G.; Zhang X.; Jones L. O.; Alzola J. M.; Mukherjee S.; Feng L. W.; Zhu W.; Stern C. L.; Huang W.; Yu J.; et al. Systematic Merging of Nonfullerene Acceptor π-Extension and Tetrafluorination Strategies Affords Polymer Solar Cells with > 16% Efficiency. J. Am. Chem. Soc. 2021, 143 (16), 6123–6139. 10.1021/jacs.1c00211. 33848146

[ref30] Wan X.; Li C.; Zhang M.; Chen Y. Acceptor-donor-acceptor type molecules for high performance organic photovoltaics - chemistry and mechanism. Chem. Soc. Rev. 2020, 49 (9), 2828–2842. 10.1039/D0CS00084A. 32239058

[ref31] Lin Y.; Wang J.; Zhang Z. G.; Bai H.; Li Y.; Zhu D.; Zhan X. An electron acceptor challenging fullerenes for efficient polymer solar cells. Adv. Mater. 2015, 27 (7), 1170–1174. 10.1002/adma.201404317. 25580826

[ref32] Zhao W.; Li S.; Yao H.; Zhang S.; Zhang Y.; Yang B.; Hou J. Molecular Optimization Enables over 13% Efficiency in Organic Solar Cells. J. Am. Chem. Soc. 2017, 139 (21), 7148–7151. 10.1021/jacs.7b02677. 28513158

[ref33] Yao H.; Ye L.; Hou J.; Jang B.; Han G.; Cui Y.; Su G. M.; Wang C.; Gao B.; Yu R.; et al. Achieving Highly Efficient Nonfullerene Organic Solar Cells with Improved Intermolecular Interaction and Open-Circuit Voltage. Adv. Mater. 2017, 29 (21), 1700254 10.1002/adma.201700254. 28370383

[ref34] Yuan J.; Zhang Y.; Zhou L.; Zhang G.; Yip H.-L.; Lau T.-K.; Lu X.; Zhu C.; Peng H.; Johnson P. A.; et al. Single-Junction Organic Solar Cell with over 15% Efficiency Using Fused-Ring Acceptor with Electron-Deficient Core. Joule 2019, 3 (4), 1140–1151. 10.1016/j.joule.2019.01.004.

[ref35] Li C.; Zhou J.; Song J.; Xu J.; Zhang H.; Zhang X.; Guo J.; Zhu L.; Wei D.; Han G.; et al. Non-fullerene acceptors with branched side chains and improved molecular packing to exceed 18% efficiency in organic solar cells. Nat. Energy 2021, 6 (6), 605–613. 10.1038/s41560-021-00820-x.

[ref36] Cui Y.; Yao H.; Zhang J.; Xian K.; Zhang T.; Hong L.; Wang Y.; Xu Y.; Ma K.; An C.; et al. Single-Junction Organic Photovoltaic Cells with Approaching 18% Efficiency. Adv. Mater. 2020, 32 (19), 1908205 10.1002/adma.201908205. 32227399

[ref37] He D.; Zhao F.; Wang C.; Lin Y. Non-Radiative Recombination Energy Losses in Non-Fullerene Organic Solar Cells. Adv. Funct. Mater. 2022, 32 (19), 2111855 10.1002/adfm.202111855.

[ref38] Cowan S. R.; Roy A.; Heeger A. J. Recombination in polymer-fullerene bulk heterojunction solar cells. Phys. Rev. B 2010, 82 (24), 245207 10.1103/PhysRevB.82.245207.

[ref39] Linderl T.; Zechel T.; Brendel M.; Moseguí González D.; Müller-Buschbaum P.; Pflaum J.; Brütting W. Energy Losses in Small-Molecule Organic Photovoltaics. Adv. Energy Mater. 2017, 7 (16), 1700237 10.1002/aenm.201700237.

[ref40] Schweda B.; Reinfelds M.; Hofstadler P.; Trimmel G.; Rath T. Recent Progress in the Design of Fused-Ring Non-Fullerene Acceptors-Relations between Molecular Structure and Optical, Electronic, and Photovoltaic Properties. ACS Appl. Energy Mater. 2021, 4 (11), 11899–11981. 10.1021/acsaem.1c01737. 35856015 PMC9286321

[ref41] Zhang X.; Li C.; Qin L.; Chen H.; Yu J.; Wei Y.; Liu X.; Zhang J.; Wei Z.; Gao F.; et al. Side-Chain Engineering for Enhancing the Molecular Rigidity and Photovoltaic Performance of Noncovalently Fused-Ring Electron Acceptors. Angew. Chem., Int. Ed. 2021, 60 (32), 17720–17725. 10.1002/anie.202106753. 34060196

[ref42] Zhang X.; Qin L.; Yu J.; Li Y.; Wei Y.; Liu X.; Lu X.; Gao F.; Huang H. High-Performance Noncovalently Fused-Ring Electron Acceptors for Organic Solar Cells Enabled by Noncovalent Intramolecular Interactions and End-Group Engineering. Angew. Chem., Int. Ed. 2021, 60 (22), 12475–12481. 10.1002/anie.202100390. 33749088

[ref43] Luo D.; Brabec C. J.; Kyaw A. K. K. Non-fused ring electron acceptors for high-performance and low-cost organic solar cells: Structure-function, stability and synthesis complexity analysis. Nano Energy 2023, 114, 108661 10.1016/j.nanoen.2023.108661.

