# Supplementary material for: Synthesis of 2,7-Dihydrooxepine-spirodithiophene Derivatives via an Intramolecular Ring Closure Reaction
Source: ACS Omega. 2024 Dec 17;9(52):51285–94. doi: 10.1021/acsomega.4c07409 (PMC11696398; doi:10.1021/acsomega.4c07409)
Supplement: Supplementary file 1 — ao4c07409_si_001.pdf [file ao4c07409_si_001.pdf]

# Synthesis of 2,7-dihydrooxepine-spirodithiophen derivatives via an intramolecular ring closure reaction

Xiaochen Liu[a,b], Tian Gao[a], Ruiyao Wang[d], Jianan Liu[b], Hua-Jun Shawn Fan[b], Jin Fang[c], Changqi Ma*[c],and Yi Lin*[a]

[a]*Department of Chemistry, Xi'an Jiaotong-Liverpool University,Suzhou, Jiangsu, 215000, PR China*

[b]*Department of Chemical Engineering, Sichuan University of Science & Technology, Zigong, Sichuan, 643000, PR China*

[c] *i-Lab & Printable Electronics Research Center, Suzhou Institute of Nano-Tech and Nano-Bionics, Chinese Academy of Sciences, Suzhou 215123, PR China*

[d] *Wisdom Lake Academy of Pharmacy, Xi'an Jiaotong-Liverpool University, Suzhou, Jiangsu, 215000, PR China*

## Table of Contents

| | Page |
|---|---|
| Experimental sections | S1 |
| Single Crystal X-Ray Diffraction spectra | S4 |
| NMR and Mass Spectra | S5 |
| UV-vis spectra and table of photophysical/electrochemical parameters | S15 |
| Film and Simulated absorption spectra based on TD-DFT calculation | S16 |
| HOMO and LUMO orbital sets based on TD-DFT calculation | S17 |

**Experimental sections**

**Computational Method.**

The density functional method B3LYP[1] used in this paper was embedded in G09 and the **DSOCT-(TFIC)$_6$** and **DSOCT-(FIC)$_6$** geometry optimization was first optimized with 3-21G basis set[2], followed by 6-31G(d,p)[3] with PCM solvation model(solvent = chloroform). The optimized geometries in this paper were confirmed to be the lowest energy conformations by separate frequency calculations using the same method and basis set.

**Characterization**

The UV-VIS absorption spectrum was recorded on a PerkinElmer Lambda750 spectrometer from dilute solutions in CHCl3. Three actual different concentration of solutions (approximate at $10^{-3}$ mol/L) were prepared for each tested sample. For each solution, a series of further dilutions was prepared to a range from $10^{-7}$-$10^{-5}$mol/L. The absorption of was recorded under irradiation of UV light, and the absorbance data point for the concentration was plotted at maximum absorption wavelength ($\lambda_{max}$). The molar absorptivity (ε) was calculated from the linear relationship between S2/S17 concentration and maximum absorption base on Beer-Lambert model, $A = \varepsilon \cdot c \cdot l$. The film on quartz were prepared by spin-coating with chloroform solution. The fluorescence solution spectrum was recorded on FluroMax-4 fluorescence spectrometer, the concentration of solution ranges from $10^{-4}$-$10^{-5}$mol/L as

prepared above. The film on quartz were prepared by spin-coating with chloroform solution, using Cary-300 spectrometer. The electrotechnical properties were analyzed via cyclic voltammetry (CV) technology using RST3000 electrochemical workstation (Reistech, Suzhou). A solution of tetrabutylammonium hexafluorophosphate (TBAPF6, 0.1 mol/L) in dried DCM was obtained. The cyclic voltammogram was obtained at a scan rate of 100 mV/s. Pt wire (Φ = 1.0 nm) embedded in a Teflon column was used as a working electrode, and a Pt sheet and Ag/AgCl electrodes were served as the counter and reference electrodes, respectively.

**Device fabrication**

Organic solar cells were fabricated on glass with the conventional structure of ITO/PEDOT:PSS/Active film/PFN-Br/Al. The substrates were cleaned using detergent, deionized water, acetone and isopropanol consecutively for every 30 min under ultrasonic. The glass substrates were treated by ultraviolet ozone. A thin layer of PEDOT: PSS (Heraeus Clevios P VPA 4083) was spin coated onto precleaned ITO-coated glass substrates at 4000 rpm for 40 s and then annealed at 150 $^{o}$C for15 min in air. Then the substrates were transferred to a glovebox, the active layer (D : A = 1:1) was spin coated at 10 mg/mL in chloroform solution at 2500 rpm for 60 s, and was baked at 100 $^{o}$C for 5 min. A thin layer of PFN-Br (~10 nm)was spin coated onto processed active layer by 3000 rpm for 30s, And finally, a layer of Al (100 nm) electrode were deposited via thermal evaporation at a pressure about 1  10 Pa to complete the device with an effective area of 0.09 $cm^2$. The devices were measured in glove box using a Keithely 2400 source meter under illumination with simulated AM 1.5G sunlight, filtered by Schott GG385 UV filter and a Hoya LB120 daylight filter.

**Single crystal X-ray diffraction analysis of DSOCT-Br$_6$ (9)**

A crystal of a size 0.30*0.25*0.06 mm was mounted on a glass fiber with expoxy glue. Data collection was performed on a Bruker D8 VENTURE Photon II diffractometer with graphite monochromated Mo Kα radiation (λ = 0.71073 Å) at room temperature, operating at 50 kV and 30 mA. Data were processed on a PC using the Bruker AXS Crystal Structure Analysis Package[4]. Data collection: Bruker APEX2; cell refinement: Bruker SAINT; Data reduction: Bruker SAINT.

Structure solution: SHELXS-97[5]; Structure refinement: SHELXL-2016/6[6]; Molecular graphics: Bruker SHELXTL[5]; Publication materials: Bruker SHELXTL[5]. All non-hydrogen atoms were located from the difference-Fourier maps and refined anisotropically. The hydrogen atoms on C3 and C16 were located from difference Fourier maps, while all other H-atoms were placed geometrically and refined using a riding model with common isotropic displacement factors Uiso(H) = 1.2 Ueq (parent C-atom) or 1.5 Ueq (methyl C-atom). The two hexyl groups were disordered at their two carbon atoms at the ends. SHELX commands, EADP, SADI and PART were applied to resolve the disorder.

**CCDC Deposition**

Summary of Data - Deposition Number 2350287

Compound Name:

Data Block Name: data_yl2401a

Unit Cell Parameters: a 10.4162(5) b 15.0277(7) c 15.2242(7) P-1

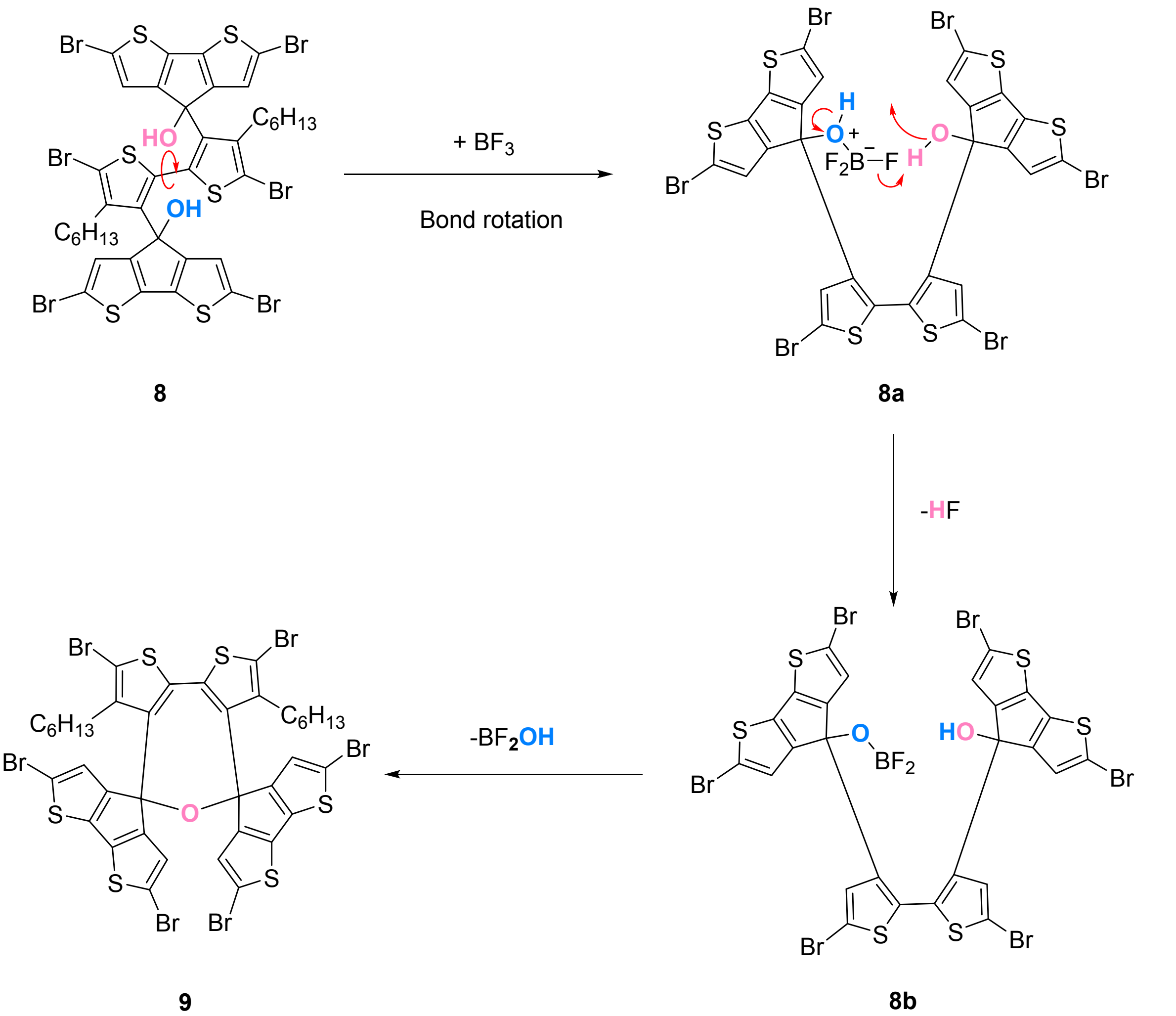


**Scheme S 1. Proposed mechanism of intramolecular dehydration towards 2,7-dihydrooxpine conformation**

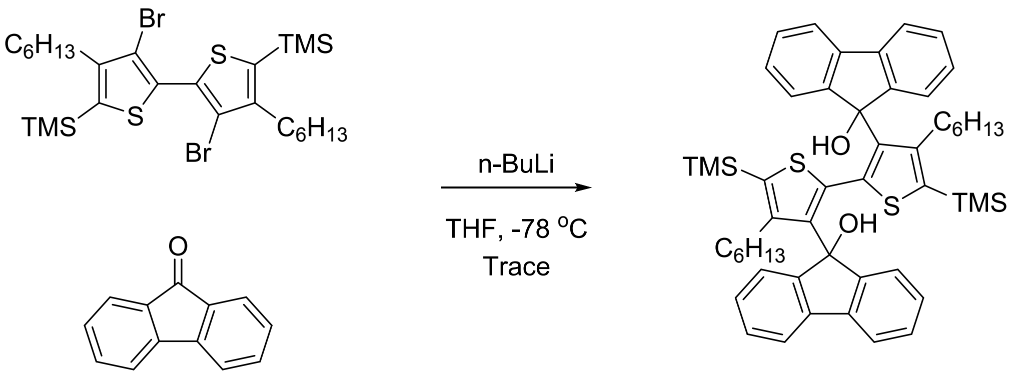


**Scheme S 2. Try to react with 9*H*-fluoren-9-one.**

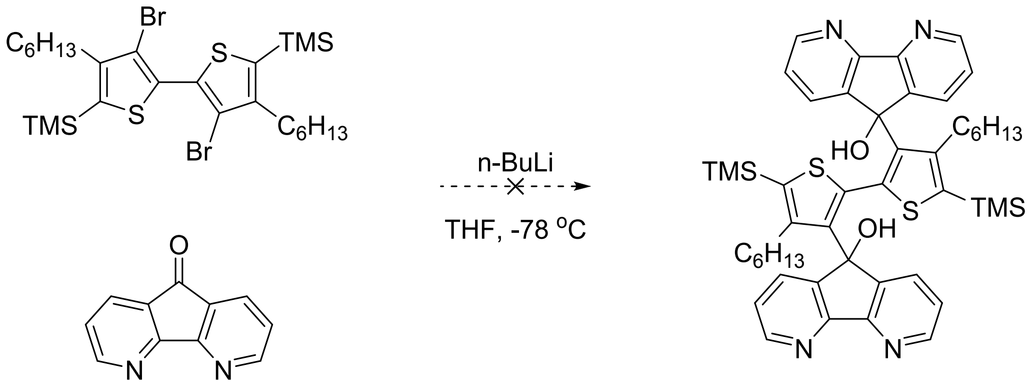


**Scheme S 3. Try to react with 5*H*-cyclopenta[2,1-*b*:3,4-*b'*]dipyridin-5-one.**

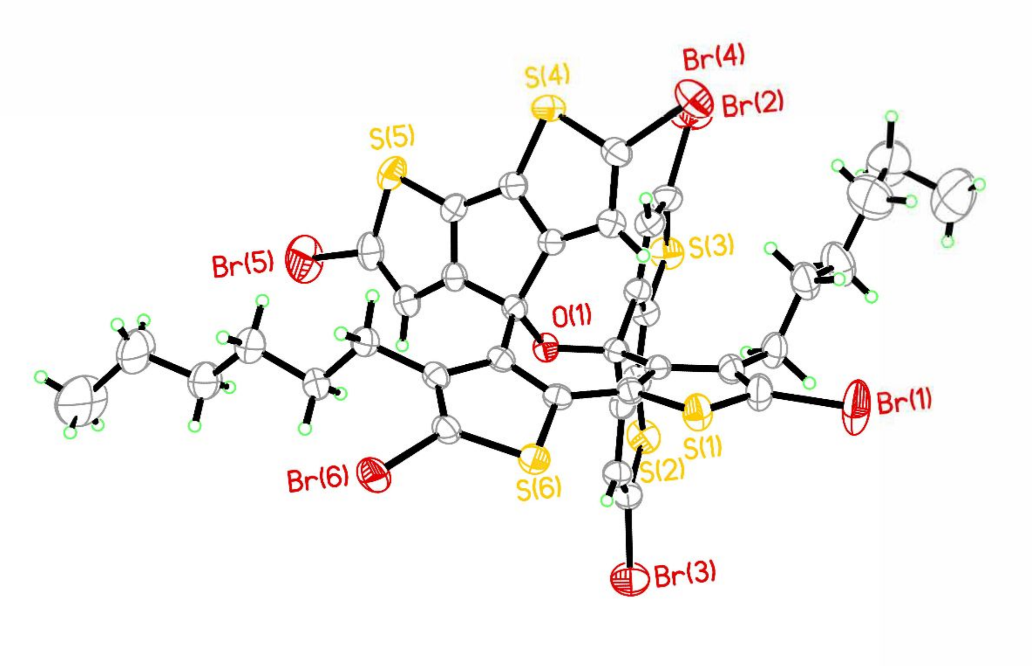


**Figure S 1. The molecular structure of DSOCT-$Br_6$(9)**

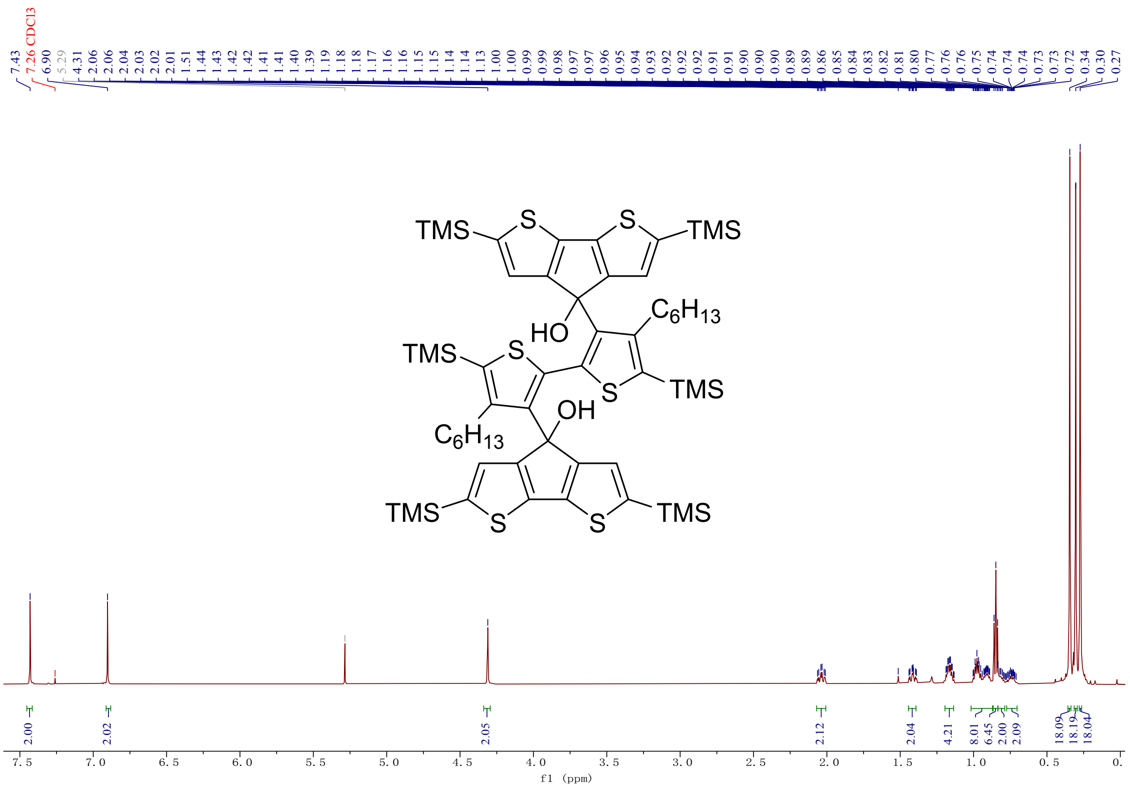


**Figure S 2. ¹H NMR of 6b**

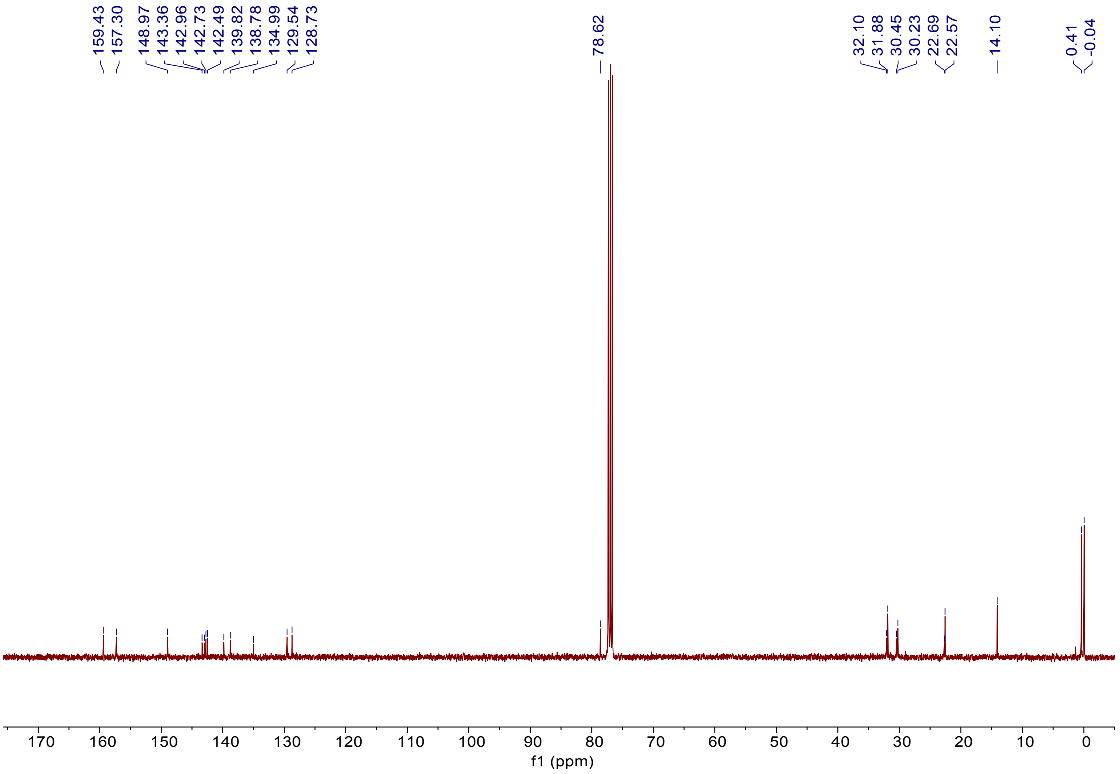


**Figure S 3. ¹³C NMR of 6b**

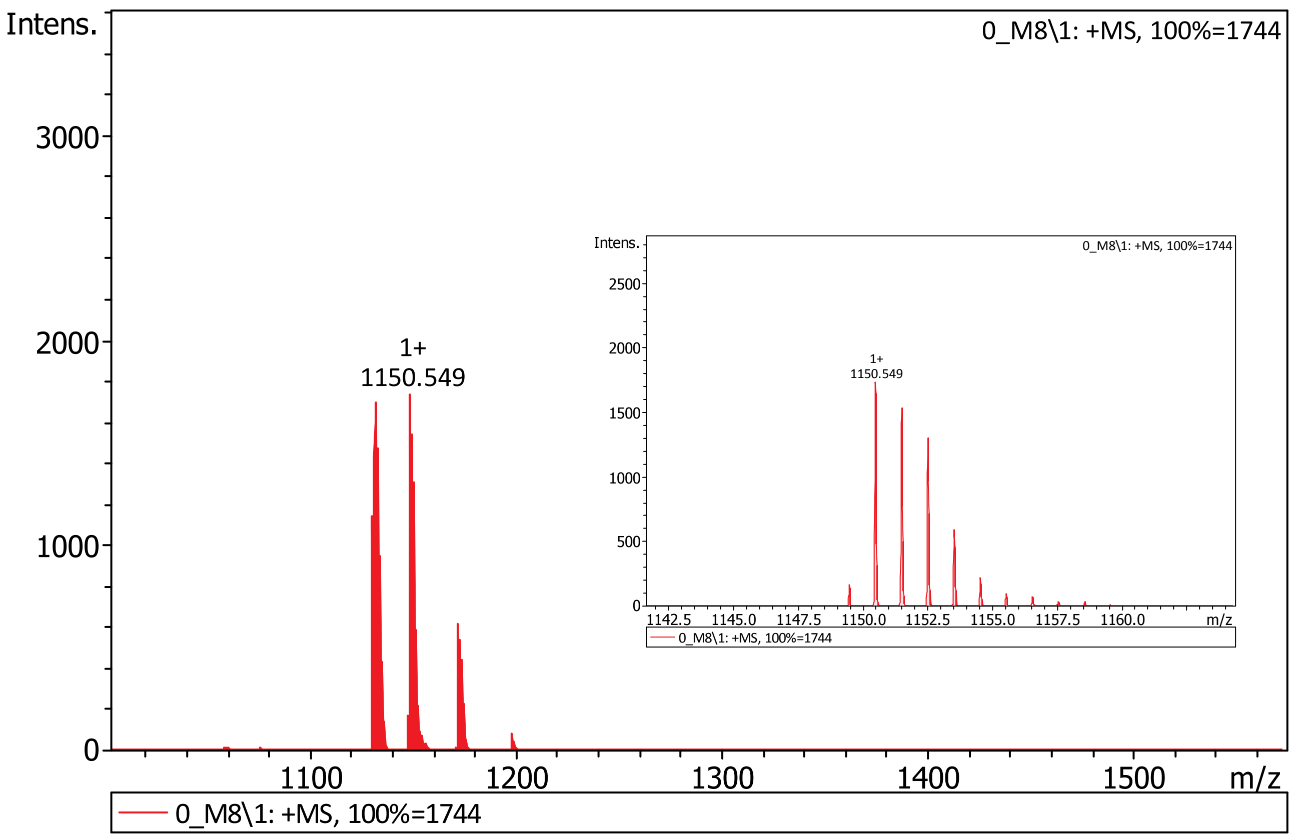


**Figure S 4. MALDI-TOF-MS of 6b**

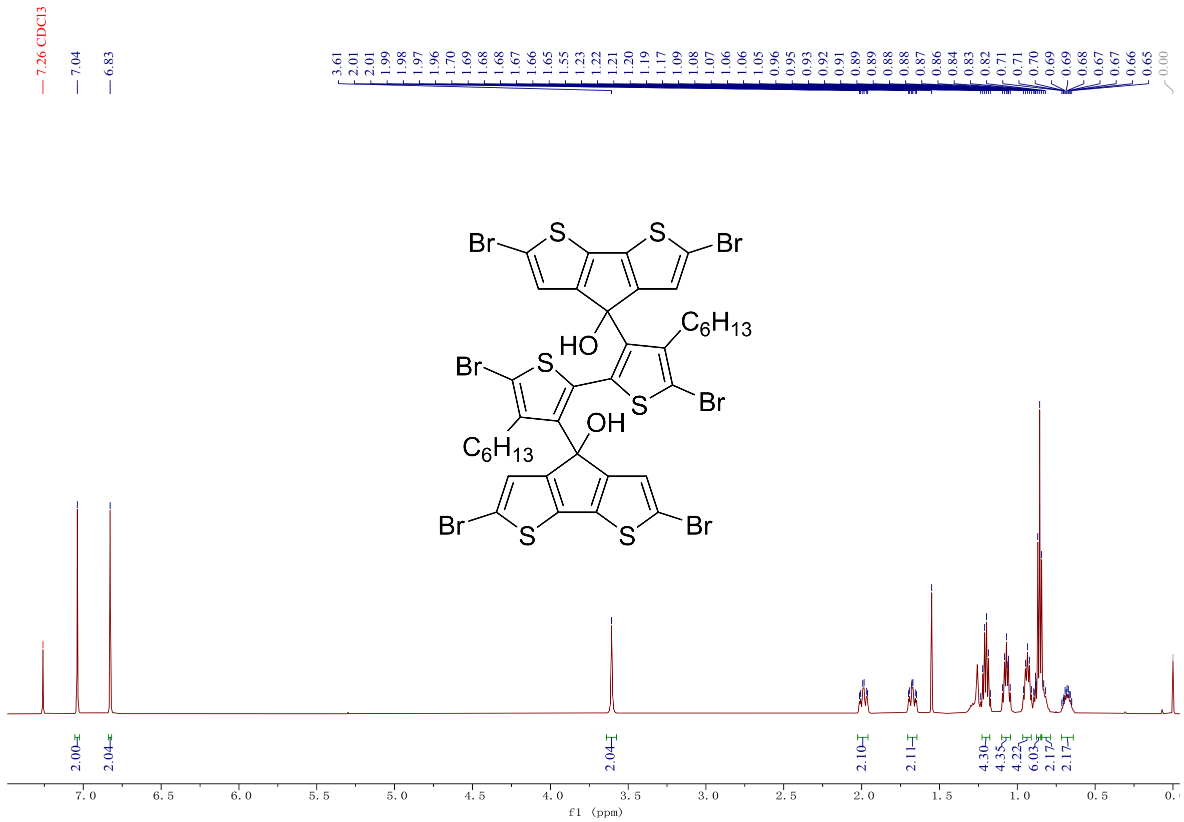


**Figure S 5. $^1$H NMR of 8**

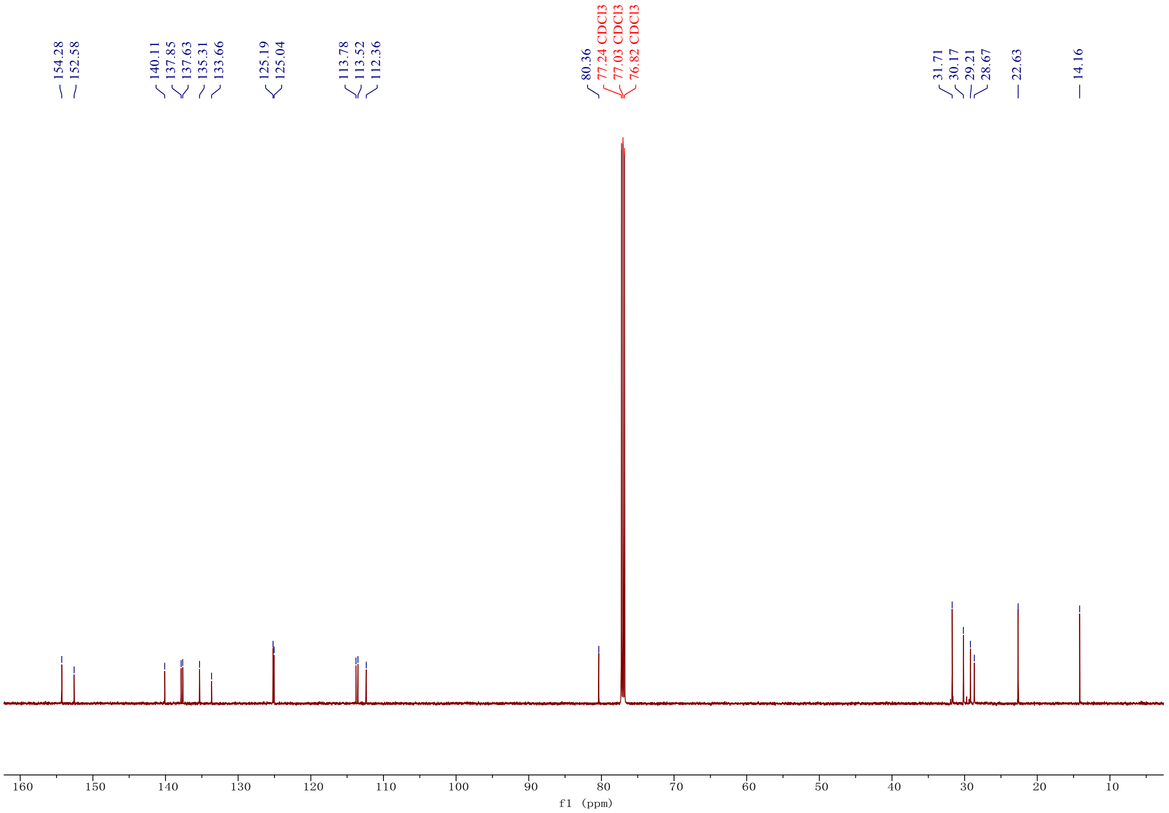


**Figure S 6 $^{13}C$ NMR of 8**

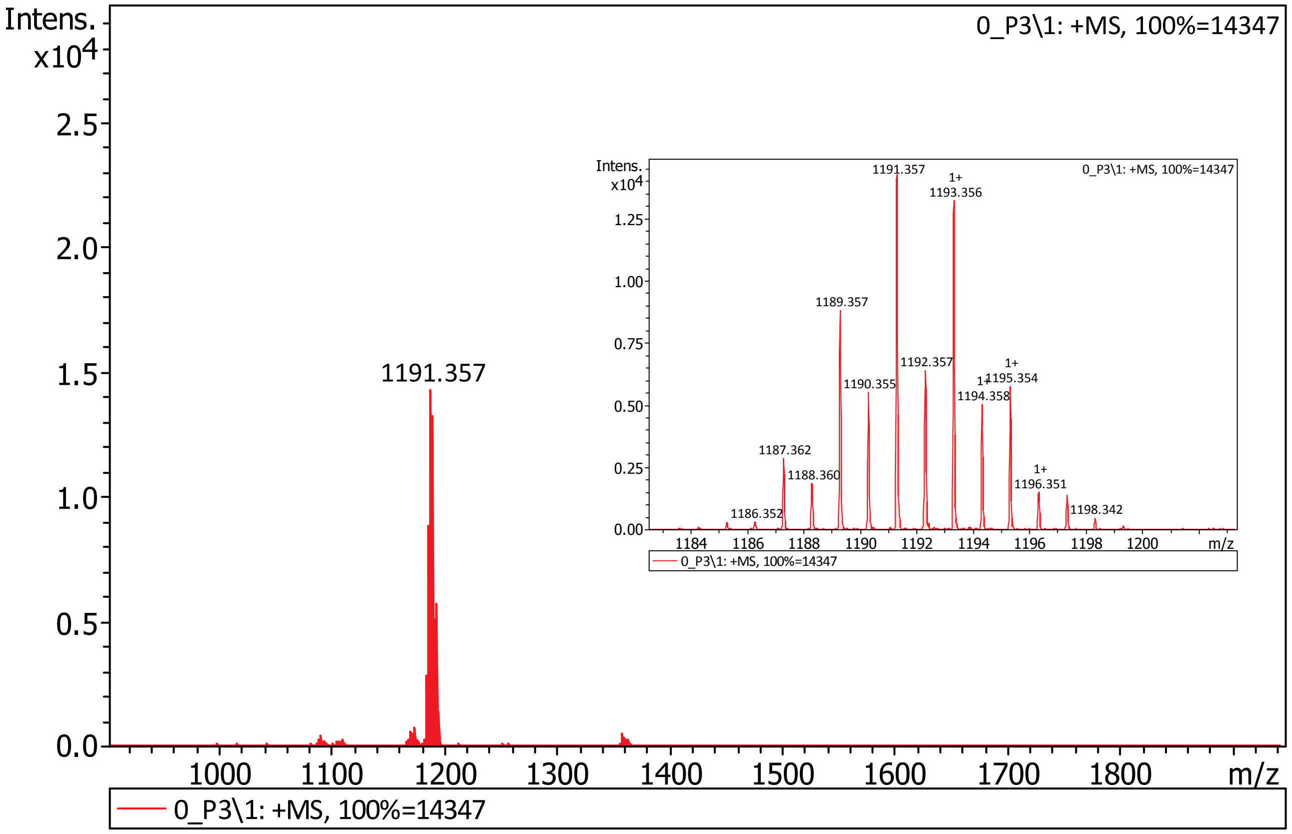

**Figure S 7. MALDI-TOF-MS of 8**

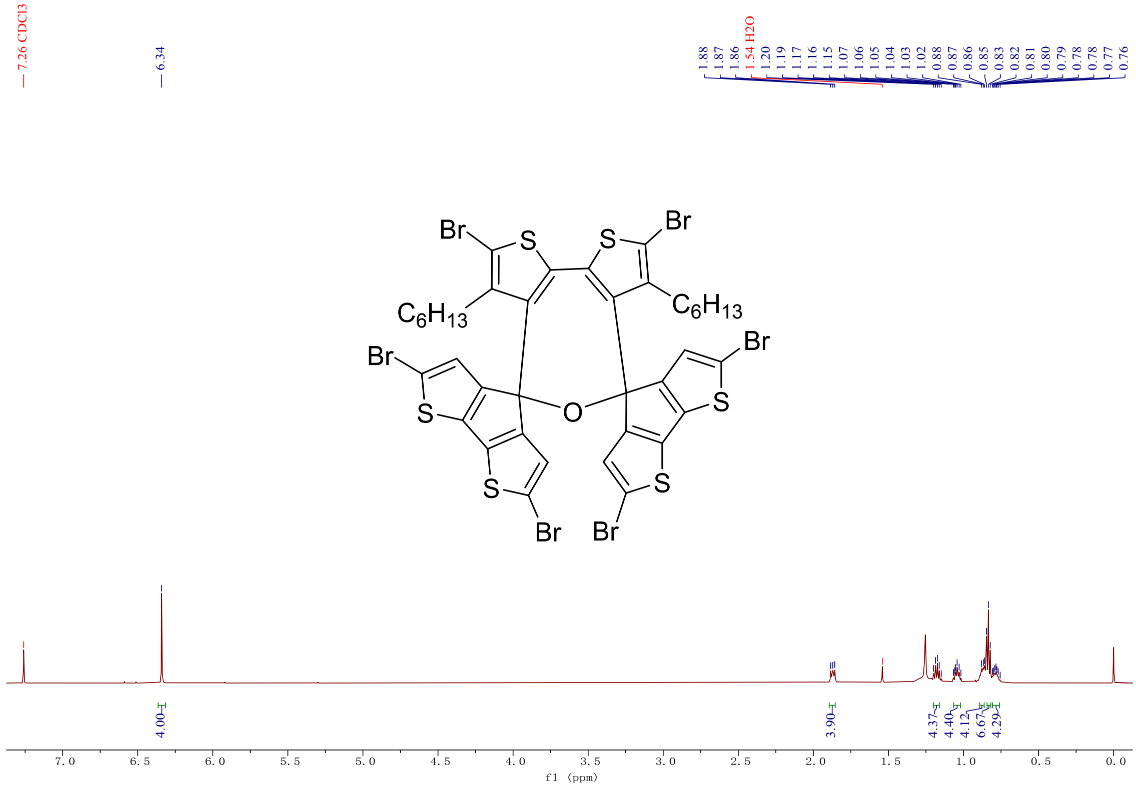


**Figure S 8 $^{1}$H NMR of DSOCT-Br$_6$ (9)**

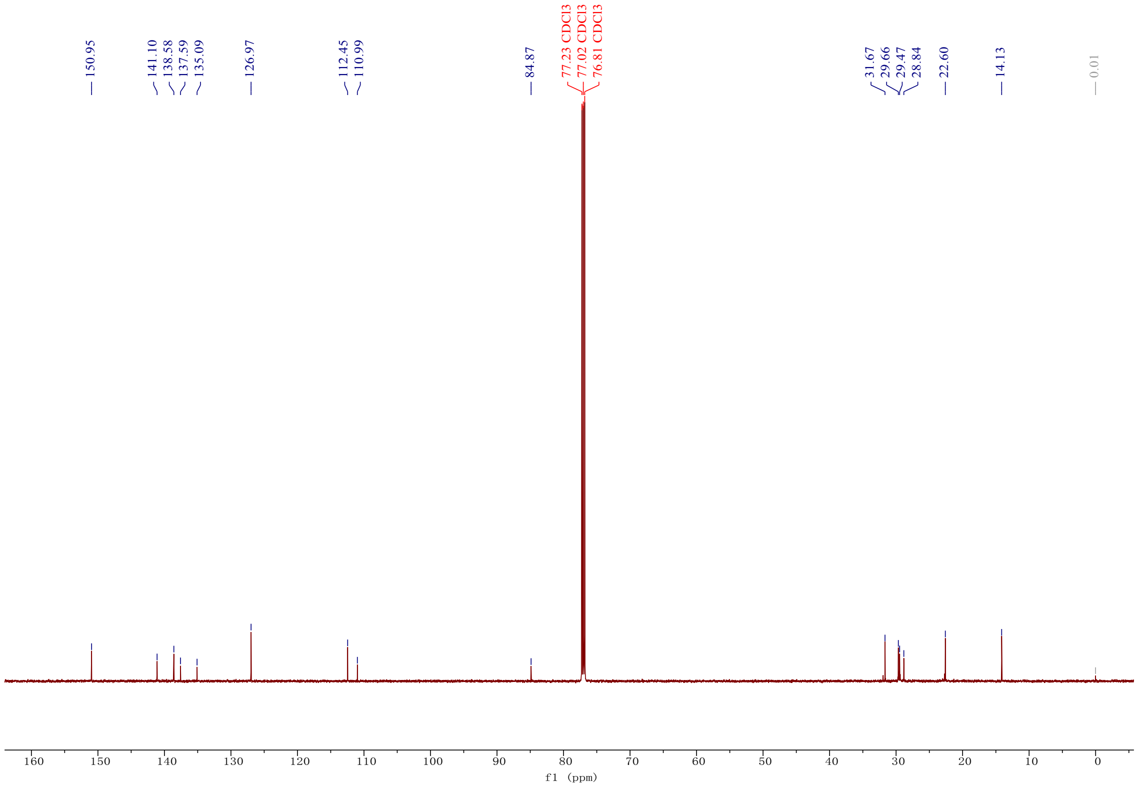


**Figure S 9. $^{13}$C NMR of DSOCT-Br$_6$ (9)**

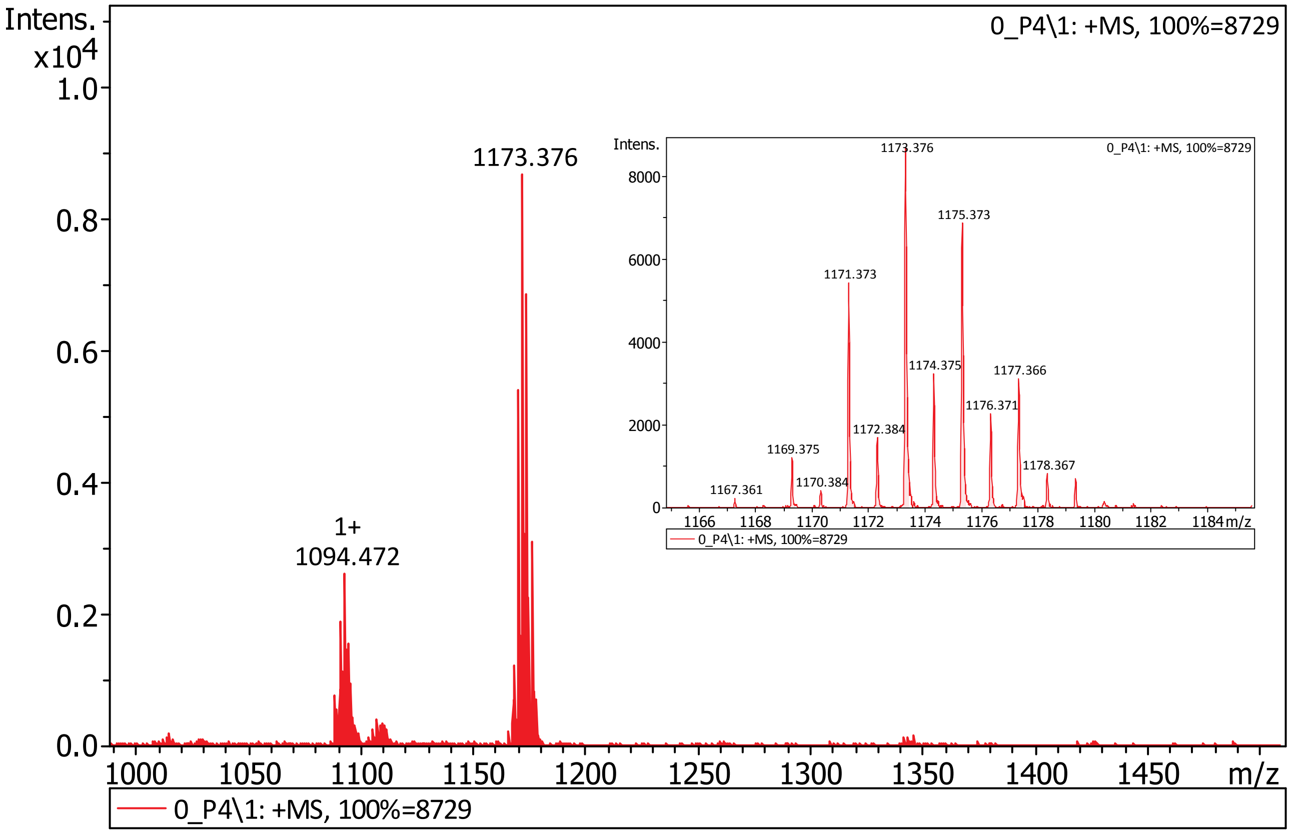


**Figure S 10. MALDI-TOF-MS of DSOCT-$Br_6$ (9)**

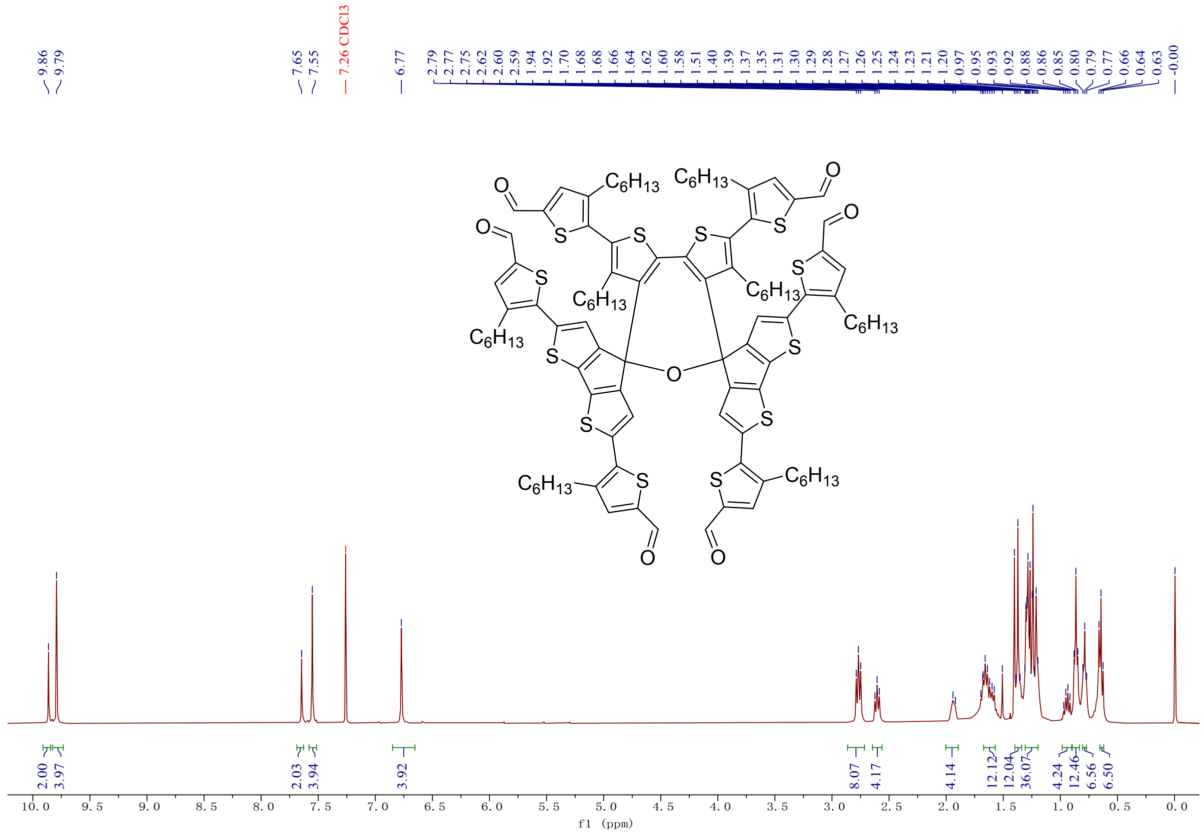


**Figure S 11.** $^1$**H NMR of DSOCT-$(TCHO)_6$**

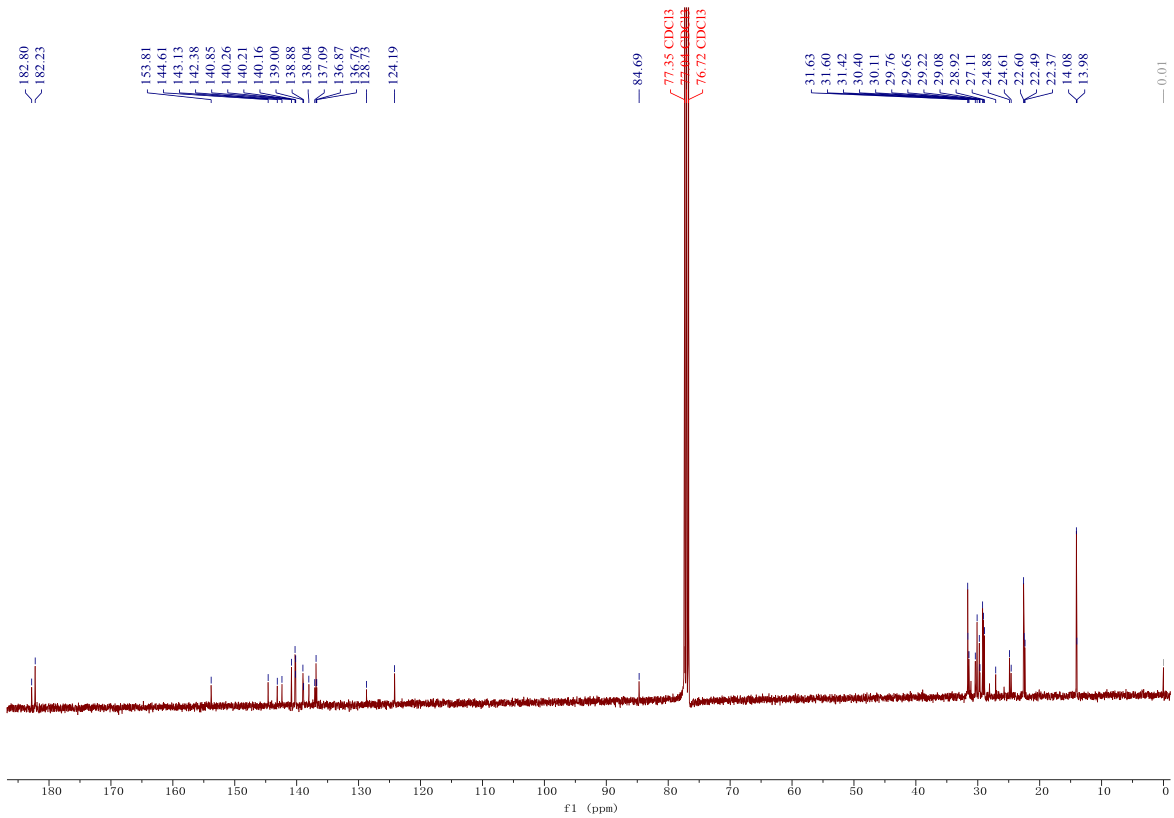

**Figure S 12. $^{13}$C NMR of DSOCT-$(TCHO)_6$**

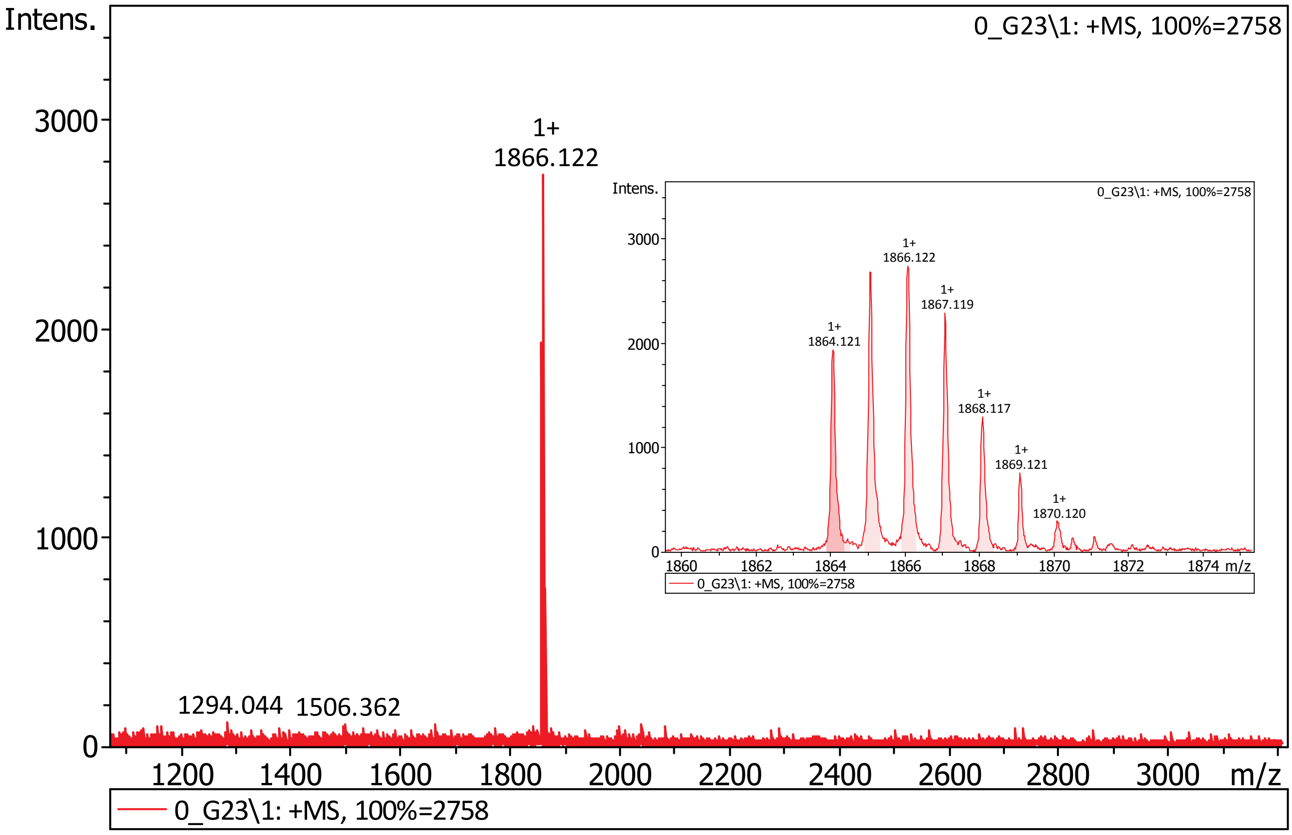

**Figure S 13. MALDI-TOF-MS of DSOCT-(CHO)$_6$**

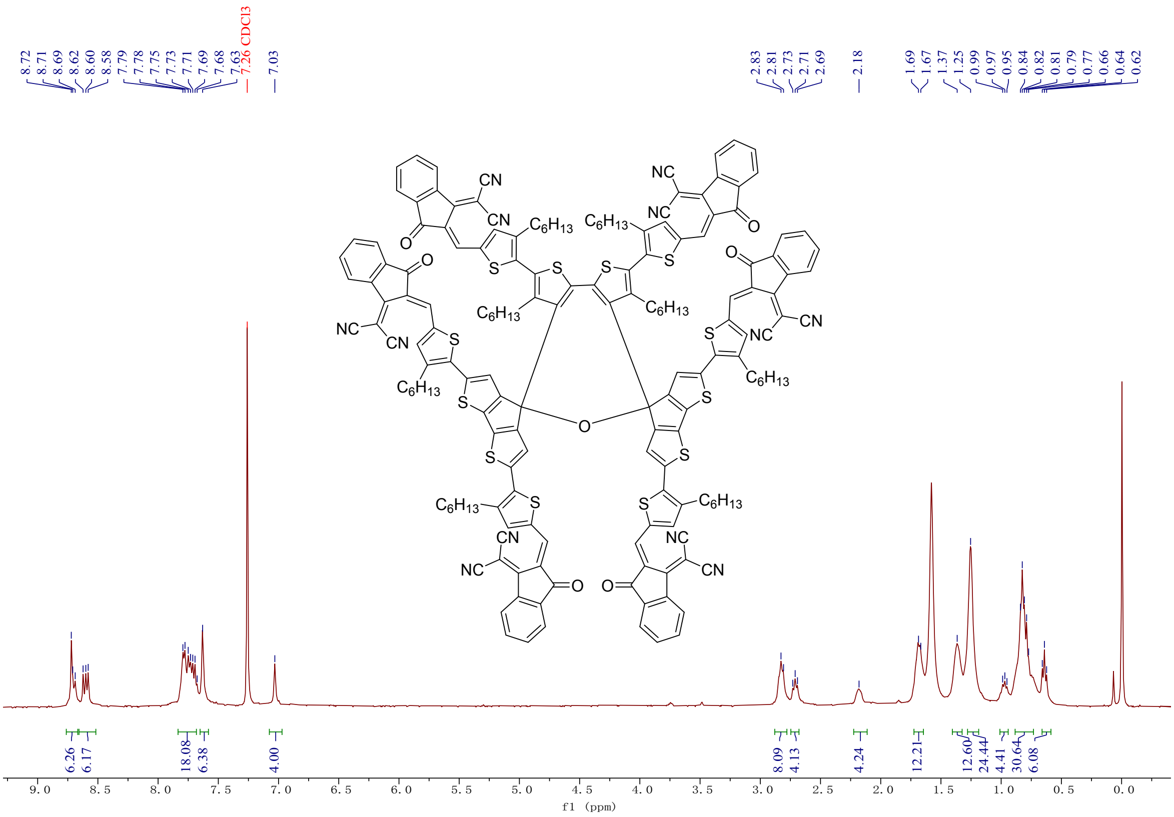


**Figure S 14. $^1$H NMR of DSOCT-(TIC)$_6$**

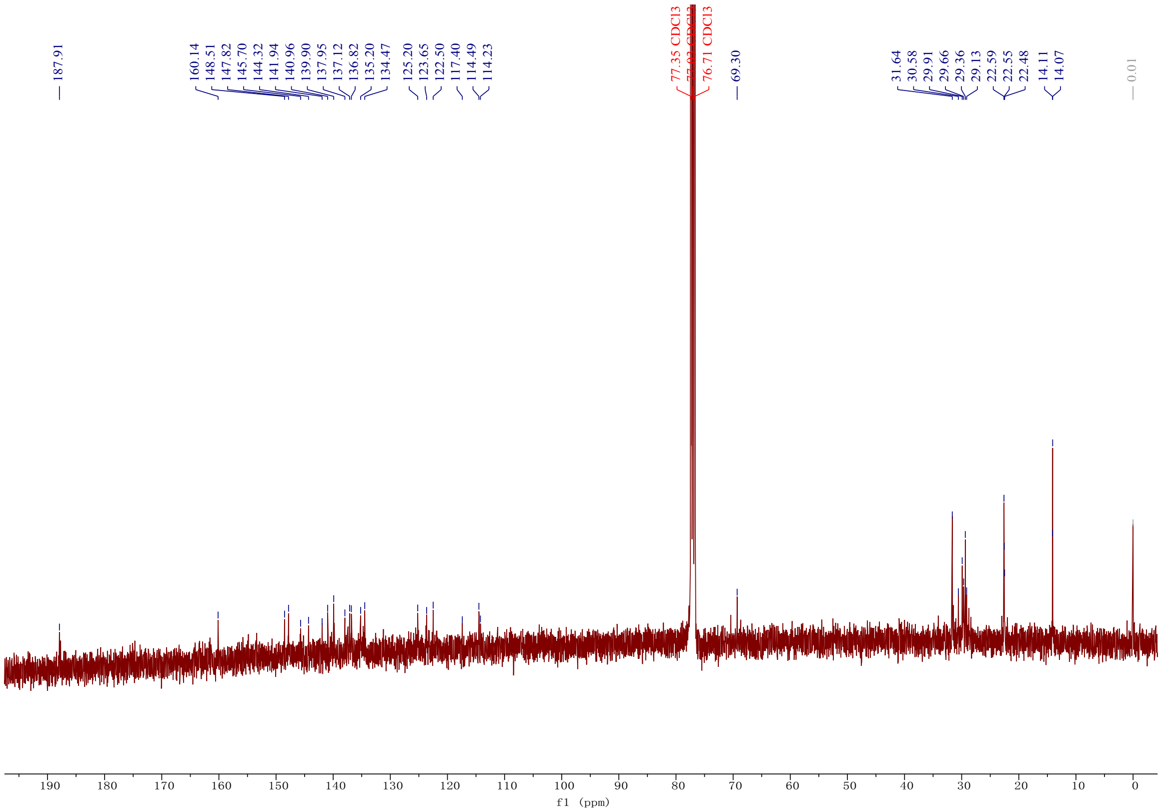

**Figure S 15.** $^{13}$**C NMR of DSOCT-(TIC)**$_6$

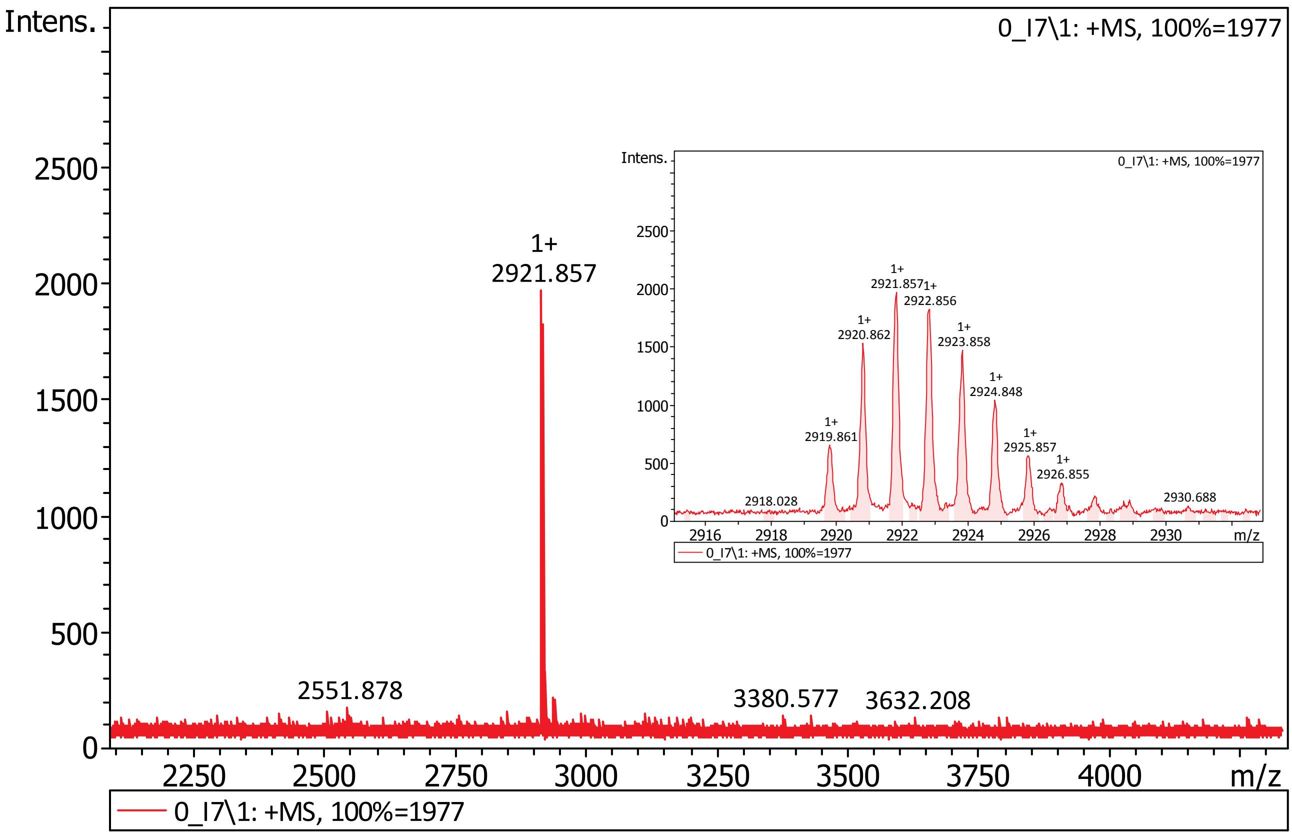


**Figure S 16. MALDI-TOF-MS of DSOCT-(TIC)**$_6$

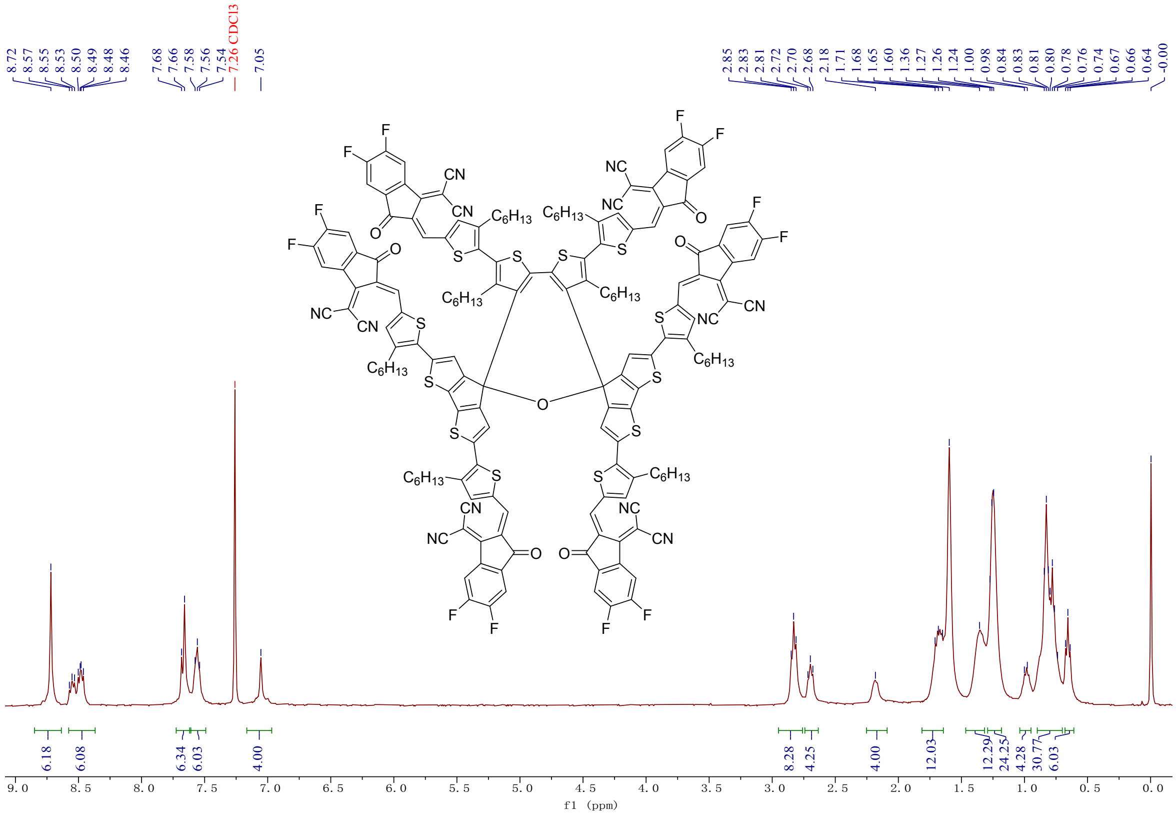

Figure S 17. $^{1}$H NMR of DSOCT-(TFIC)$_6$ \

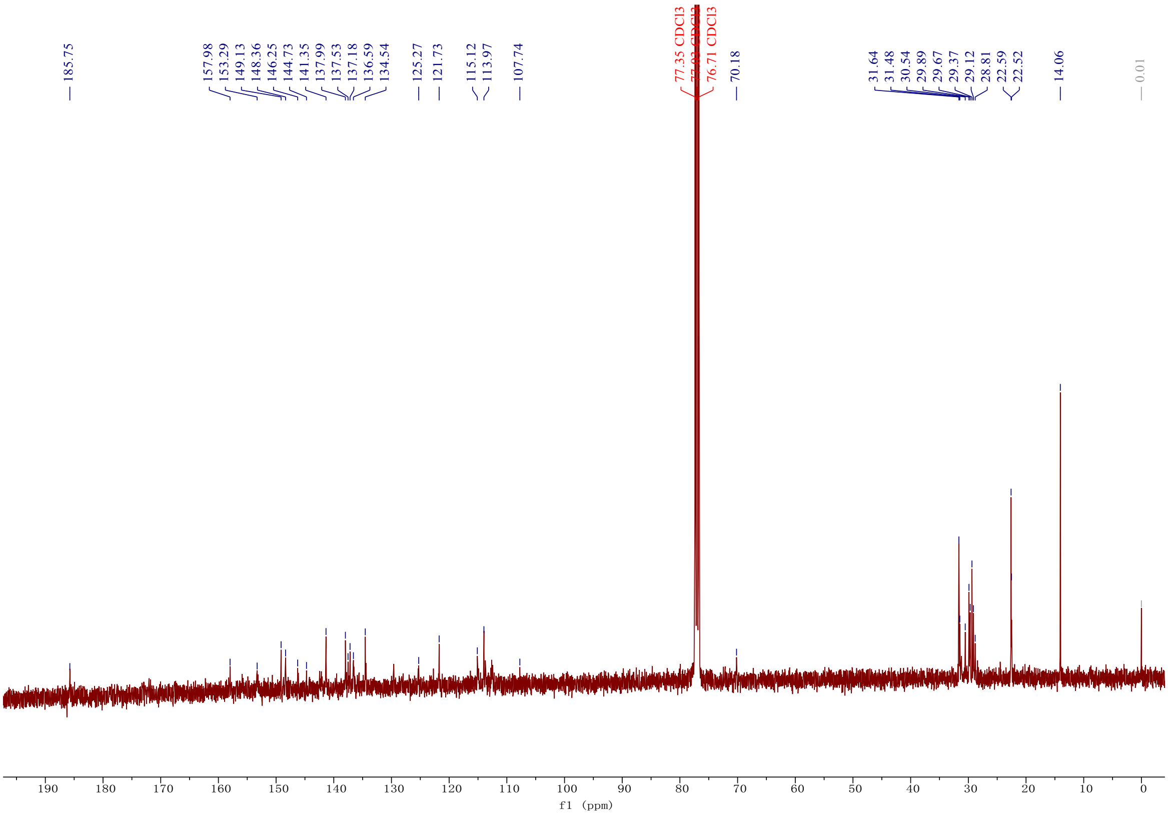


Figure S 18. $^{13}$C NMR of DSOCT-(TFIC)$_6$

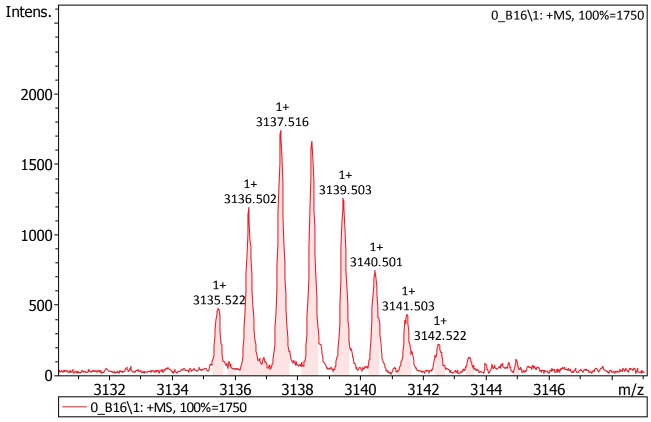

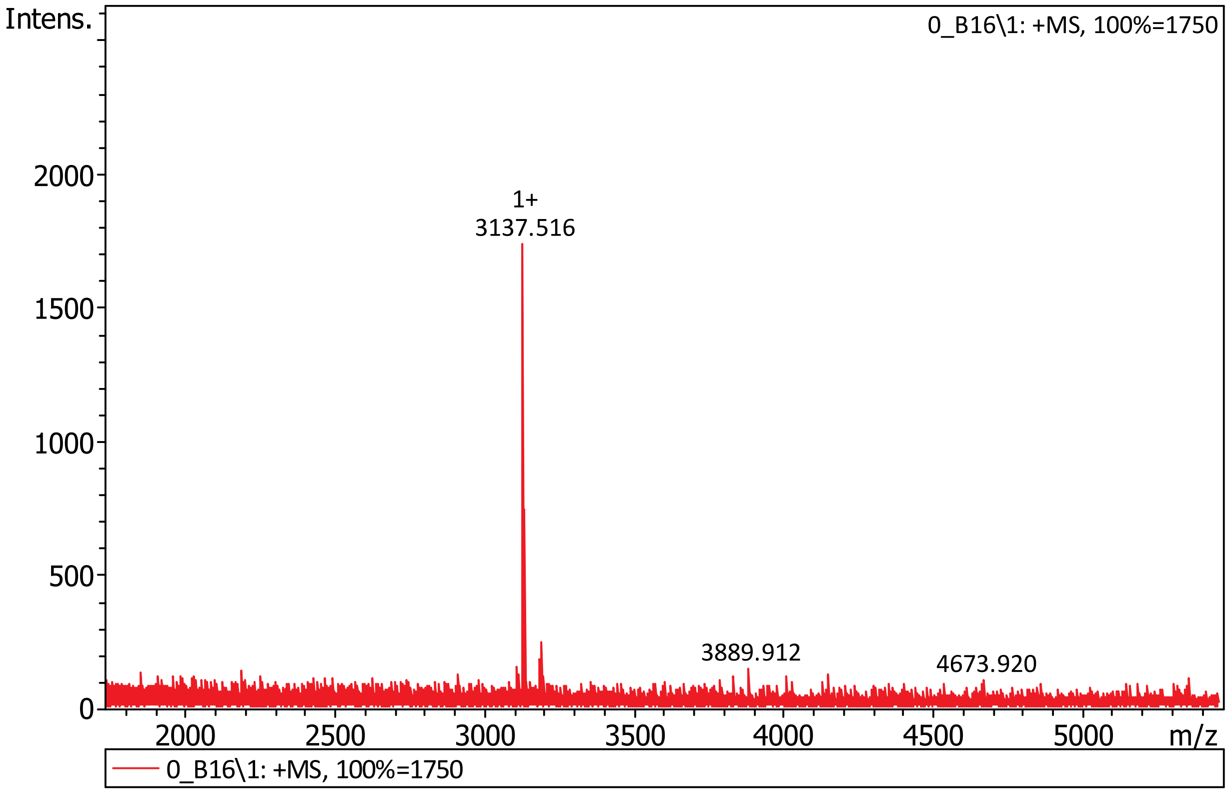


Figure S 19. MALDI-TOF-MS of DSOCT-(TFIC)$_6$

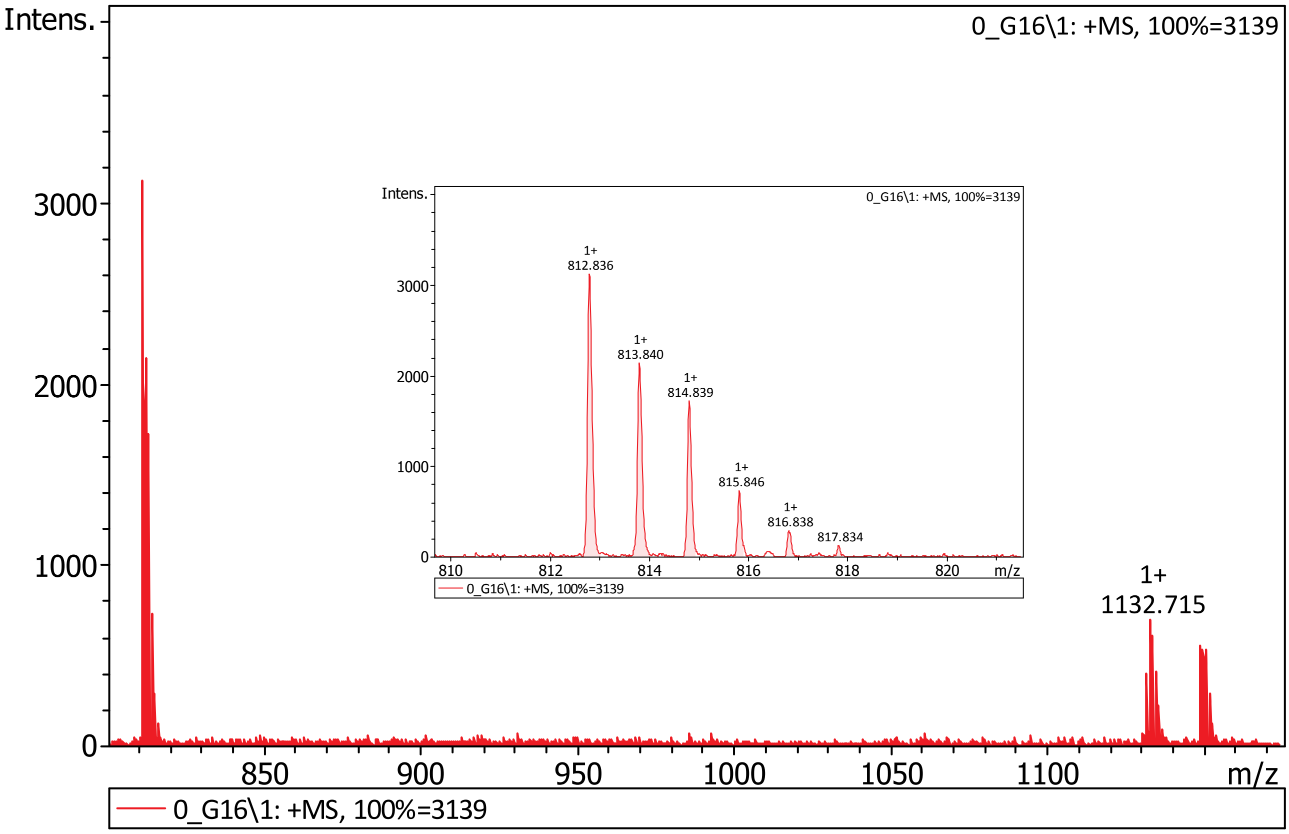

**Figure S 20. MALDI-TOF-MS of 7a**

**Table S 1. Reaction screening for synthesis of 6a and 6b**

| Entry | **2**(mg) | **3** (mg) | Yield | |
|---|---|---|---|---|
| | | | **6a** | **6b** |
| 1 | 200 | 1.0 eq | n.d. | 47 % |
| 2 | 200 | 1.2 eq. | n.d. | 59 % |
| 3 | 200 | 1.5 eq. | n.d. | 58 % |
| 4 | 200 | 2.0 eq. | n.d | 85 % |
| 5 | 1000 | 2.0 eq | n.d. | 55 % |
| 6 | 7000 | 2.0 eq | n.d. | 61 % |

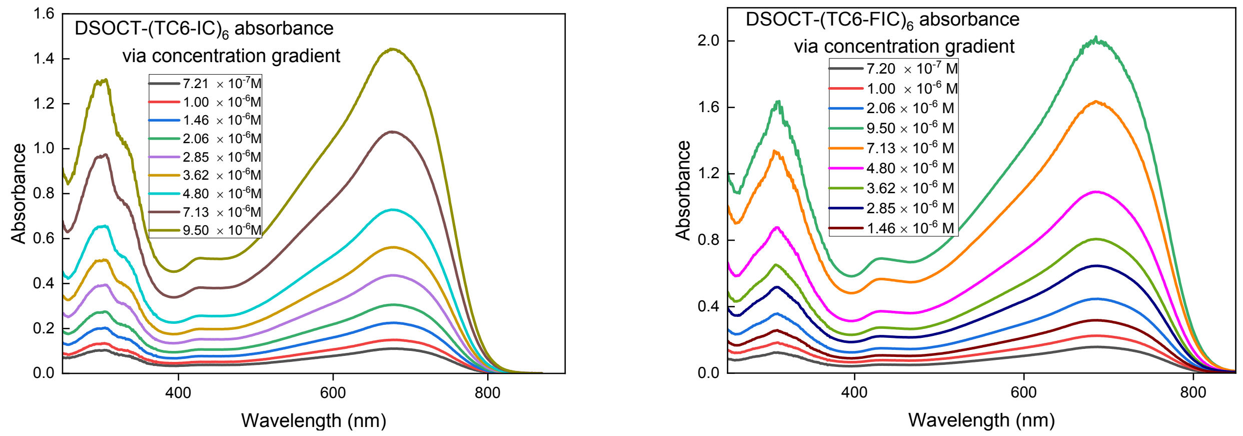


**Figure S 21. Determination of molar absorptivity of DSOCT series by series of concentration**

**Table S 2. Photophysical and Electrochemical Data of DSOCT Derivatives**

| Compound | Optical data in solution[a] | | | | Optical data in film[b] | | | Electrochemical data[c] | | | Energy Level[d] | | |
|---|---|---|---|---|---|---|---|---|---|---|---|---|---|
| | $\lambda_{max}^{sol.}$ [nm] | $\varepsilon_{max}^{sol.}$ [L mol$^{-1}$ cm$^{-1}$][e] | $\lambda_{onset}^{sol.}$ [nm] | $\lambda_{fl.}^{max.}$ [nm] | $\lambda_{max}^{film.}$ [nm] | $\lambda_{onset}^{film.}$ [nm] | $E_g^{opt.}$ [eV][f] | $E_{ox}^{01}$ [V] | $E_{ox}^{02}$ [V] | $E_{red}^{01}$ [V] | $E_{HOMO}$ [eV] | $E_{LUMO}$ [eV] | $E_g^{cv}$ [V] |
| DSOCT-(TIC)$_6$ | 677 | 151,152 | 793 | 793 | 686 | 855 | 1.56 | 0.48 | 0.66 | -0.98 | -5.58 | -4.12 | 1.46 |
| DSOCT-(TFIC)$_6$ | 686 | 215,467 | 808 | 808 | 717 | 876 | 1.53 | 0.57 | 0.73 | -0.83 | -5.67 | -4.27 | 1.40 |

[a]: in $CHCl_3$ solution at a concentration of 1.0 × $10^{-6}$ mol $L^{-1}$ for UV-vis, in $CHCl_3$ solution at a concentration of 4.8 × $10^{-6}$ mol $L^{-1}$ for FL; [b]: in $CH_2Cl_2$ at a concentration of 1.0×$10^{-3}$ mol $L^{-1}$, with $TBAPF_6$ (0.1 M) as the support electrolyte; [c]: calculated from cyclic voltammetry results, $E_{HOMO} = -(E_{ox}^{onset} + 5.10)$ (eV), $E_{LOMO} = -(E_{red}^{onset} + 5.10)$ (eV), $E_g^{CV} = (E_{ox}^{onset} - E_{red}^{onset})$ (eV); [d]: Extinction coefficient in solution was obtained by linear fitting absorbance vs. concentration; [e]: absorption onset wavelength; [f]: optical band gap $E_g^{opt}$ (eV) = 1240/$\lambda_{abs}^{onset}$

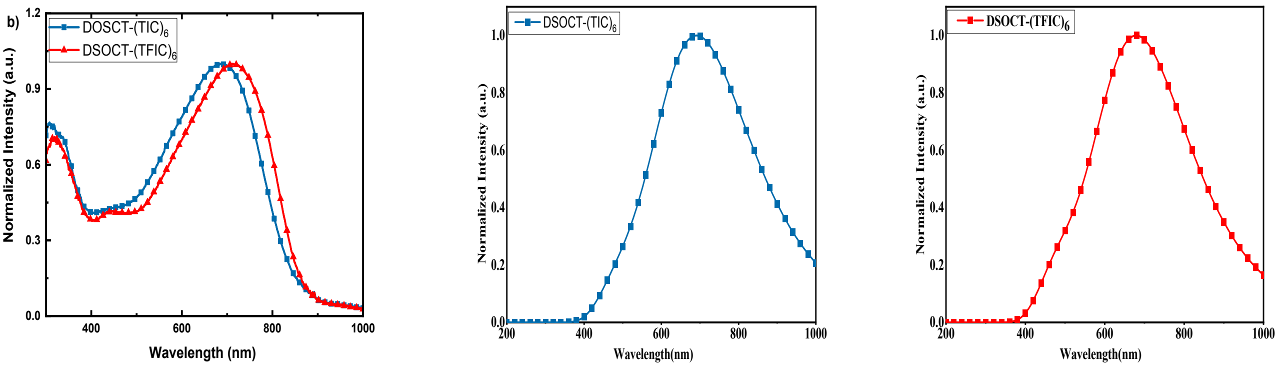


**Figure S 22. Film and Simulated absorption spectra of DSOCT-based molecules based on TD-DFT calculation.**

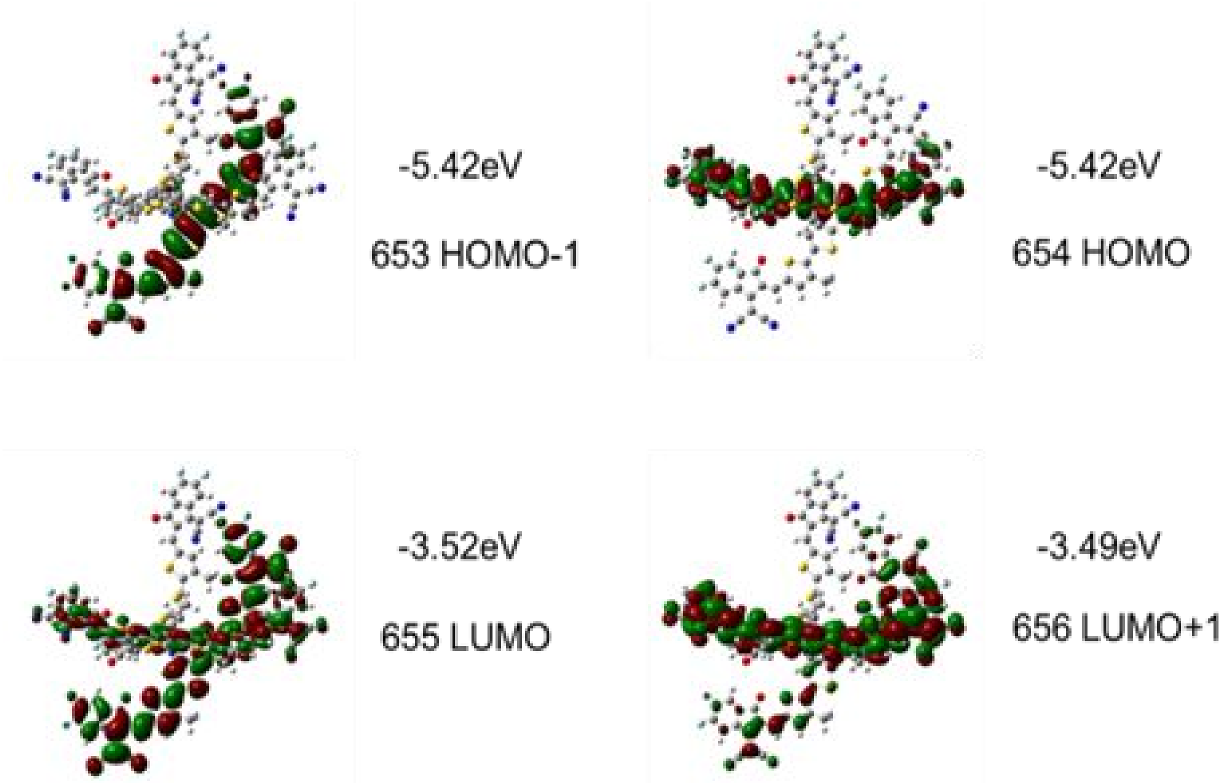


**Figure S 23. HOMO and LUMO orbital sets of DSOCT-$(TIC)_6$ and DSOCT-$(TFIC)_6$**

**Table S 3. Device performance of organic solar cells based on PM6 with DSOCT-(TIC)$_6$ and DSOCT-(TFIC)$_6$ with optimized D:A ratio of 1:1**

| Donor/ Acceptor | rpm (r/min) | $V_{oc}$ (V) | $J_{sc}$ (mA/cm$^2$) | FF | PCE (%) |
|---|---|---|---|---|---|
| PM6:DSOCT-(TIC)$_6$ | 2500 | 0.82 | 1.53 | 0.31 | 0.40 |
| | 3000 | 0.84 | 1.64 | 0.31 | 0.43 |
| | 3500 | 0.84 | 1.82 | 0.31 | 0.47 |
| PM6:DSOCT-(TFIC)$_6$ | 3000 | 0.77 | 4.34 | 0.37 | 1.25 |
| | 3500 | 0.77 | 4.49 | 0.38 | 1.30 |
| | 4000 | 0.78 | 4.75 | 0.38 | 1.39 |

**Table S 4. Optimization of DSOCT-(TIC)$_6$ and DSOCT-(TFIC)$_6$ from different views**

| | Top view | Side view |
|---|---|---|
| DSOCT-(TIC)$_6$ | 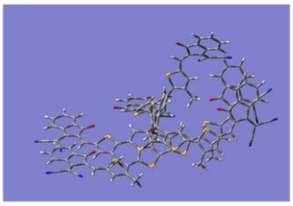 | 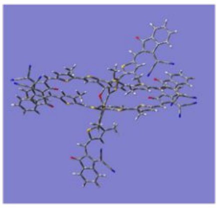 |
| DSOCT-(FTIC)$_6$ | 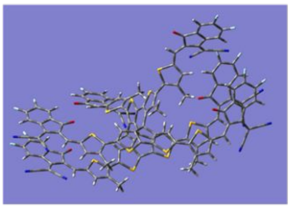 | 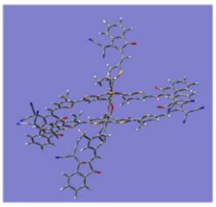 |

**Table S 5. Optimized Cartesian coordinates of DSOCT-(TIC)$_6$ (B3LYP/6-31G**/EM=GD3BJ)**

**0 1**

| | | | |
|---|---|---|---|
| C | 10.35192600 | -0.82608000 | -1.86713900 |
| C | 9.43584400 | -1.29340200 | -0.90202700 |
| C | 9.79646900 | -1.51072200 | 0.42015500 |
| C | 11.11857800 | -1.26257300 | 0.79173100 |
| C | 12.03798000 | -0.79975100 | -0.15868000 |
| C | 11.67363500 | -0.57214700 | -1.48886700 |
| C | 9.63663900 | -0.65353500 | -3.15186600 |
| C | 8.27765200 | -1.17101400 | -2.97054700 |
| C | 8.11265100 | -1.48529300 | -1.53159300 |
| H | 9.05777600 | -1.84796900 | 1.13958800 |
| H | 11.42230600 | -1.41175400 | 1.82223600 |
| H | 13.06079100 | -0.59584400 | 0.14251000 |
| H | 12.41070700 | -0.20288200 | -2.18835000 |
| O | 7.09290000 | -1.86726400 | -0.96742600 |
| C | 10.12854300 | -0.00309300 | -4.26499600 |
| C | 11.47374500 | 0.46095100 | -4.36272600 |
| N | 12.57197000 | 0.83447400 | -4.45920000 |
| C | 9.30305000 | 0.33402500 | -5.37999300 |
| N | 8.60592800 | 0.62598700 | -6.26659100 |
| C | 3.59292200 | -2.35562300 | -3.63303200 |
| C | 3.95407500 | -2.39571800 | -4.98375600 |
| C | 5.31761700 | -2.10048800 | -5.14428900 |
| C | 6.02108200 | -1.86415200 | -3.95738000 |
| S | 4.94820100 | -2.04394600 | -2.58322700 |
| H | 5.79804200 | -2.01368700 | -6.11390900 |
| C | 3.00269600 | -2.64923400 | -6.12060600 |
| H | 2.06313100 | -2.10353400 | -5.98216900 |
| H | 3.44484100 | -2.33485200 | -7.07056600 |
| H | 2.75181800 | -3.71309100 | -6.21182400 |
| C | 2.29731600 | -2.47913000 | -3.01609400 |
| C | 0.53337300 | -2.06032300 | -1.56201000 |
| C | -0.00603800 | -3.09866500 | -2.30952800 |
| S | 1.05980900 | -3.62608600 | -3.56417700 |
| C | -10.70177300 | -2.00438500 | 3.07375300 |
| C | -9.50714200 | -1.26012000 | 3.07398100 |
| C | -9.38518100 | -0.04196900 | 3.72462700 |
| C | -10.49849500 | 0.44920600 | 4.40983700 |
| C | -11.68794600 | -0.28785400 | 4.43559600 |
| C | -11.80709400 | -1.51439200 | 3.77483000 |
| C | -10.49268400 | -3.24289300 | 2.28292500 |
| C | -9.12155300 | -3.20616500 | 1.77127900 |
| C | -8.47202400 | -1.98610200 | 2.30431000 |

| | | | |
|---|---|---|---|
| H | -8.44270700 | 0.49536200 | 3.69164900 |
| H | -10.44199900 | 1.40012400 | 4.93125500 |
| H | -12.54479100 | 0.09934900 | 4.97906000 |
| H | -12.74487000 | -2.04964600 | 3.81164400 |
| O | -7.31241800 | -1.62558200 | 2.14542000 |
| C | -11.43760200 | -4.23421900 | 2.10180700 |
| C | -12.78732300 | -4.10134400 | 2.54299400 |
| N | -13.89784300 | -3.98973000 | 2.87526300 |
| C | -11.16660800 | -5.47458300 | 1.45149000 |
| N | -10.96149500 | -6.49547200 | 0.92926800 |
| C | -4.93700300 | -4.13390600 | -0.62050800 |
| C | -5.74447200 | -5.06979300 | -1.28825500 |
| C | -7.04708700 | -5.07459600 | -0.77432100 |
| C | -7.29273100 | -4.14660400 | 0.24380700 |
| S | -5.83636700 | -3.22229100 | 0.57442900 |
| H | -7.82642800 | -5.73398700 | -1.14186600 |
| C | -5.32127800 | -5.96691400 | -2.41683000 |
| H | -6.14383500 | -6.63103100 | -2.69574100 |
| H | -5.04711800 | -5.38694100 | -3.30670500 |
| H | -4.45951000 | -6.58979900 | -2.15153300 |
| C | -3.55006300 | -3.81080900 | -0.81405700 |
| C | -1.46092000 | -2.80560700 | -0.53422400 |
| C | -1.23583800 | -3.52726500 | -1.70286900 |
| S | -2.61359900 | -4.44063700 | -2.18902300 |
| C | 7.37708100 | -1.44401700 | -3.97013300 |
| H | 7.73574600 | -1.31803400 | -4.98558900 |
| C | -8.56958800 | -4.06861200 | 0.85188900 |
| H | -9.21930600 | -4.84890700 | 0.47392100 |
| C | -0.44109900 | -1.63152800 | -0.45526200 |
| C | -11.71194300 | -1.75011000 | -0.43402900 |
| C | -10.85657700 | -0.66452400 | -0.16266800 |
| C | -11.24719100 | 0.41582600 | 0.61470500 |
| C | -12.54282700 | 0.41464700 | 1.13208600 |
| C | -13.40533200 | -0.65757300 | 0.87135900 |
| C | -13.00707300 | -1.74830000 | 0.09403000 |
| C | -10.97281800 | -2.74252300 | -1.25040500 |
| C | -9.67107200 | -2.15558900 | -1.57870300 |
| C | -9.55110900 | -0.88564200 | -0.82353100 |
| H | -10.54811300 | 1.22190000 | 0.81048900 |
| H | -12.88139700 | 1.23911200 | 1.75124600 |
| H | -14.40682400 | -0.65466100 | 1.29001100 |
| H | -13.69993200 | -2.56024400 | -0.07578500 |
| O | -8.57800800 | -0.14462100 | -0.75916600 |
| C | -11.41991800 | -4.01407500 | -1.55050700 |

| | | | |
|---|---|---|---|
| C | -12.72507500 | -4.48031800 | -1.21254400 |
| N | -13.78900200 | -4.87468200 | -0.95233500 |
| C | -10.59419500 | -5.00014800 | -2.16837700 |
| N | -9.90550100 | -5.80839000 | -2.64793400 |
| C | -5.27010500 | -1.20864800 | -3.52018500 |
| C | -5.57681300 | -2.32187300 | -4.31650700 |
| C | -6.82675900 | -2.85456500 | -3.97136800 |
| C | -7.50474400 | -2.18323400 | -2.94730600 |
| S | -6.55051000 | -0.81893500 | -2.39673400 |
| H | -7.24582000 | -3.73936400 | -4.44037600 |
| C | -4.67711500 | -2.92891700 | -5.35683700 |
| H | -3.68039700 | -3.13493800 | -4.95062900 |
| H | -5.09757700 | -3.87158000 | -5.71917800 |
| H | -4.54909300 | -2.27131900 | -6.22465200 |
| C | -4.03556400 | -0.47803800 | -3.41063700 |
| C | -2.27010600 | 0.64913500 | -2.39974500 |
| C | -1.72717100 | 0.45351300 | -3.66447000 |
| S | -2.83563200 | -0.36096000 | -4.71816500 |
| C | 9.48013200 | 1.91646900 | 0.51142100 |
| C | 8.24403900 | 1.54032200 | 1.07321400 |
| C | 8.10040300 | 1.20847900 | 2.41203000 |
| C | 9.23863700 | 1.24955300 | 3.21975400 |
| C | 10.47378800 | 1.62292000 | 2.67860700 |
| C | 10.61177500 | 1.96497000 | 1.32993700 |
| C | 9.27891600 | 2.21546600 | -0.92652200 |
| C | 7.87046200 | 1.95326500 | -1.23281600 |
| C | 7.19692100 | 1.58189800 | 0.03265200 |
| H | 7.13125700 | 0.93225800 | 2.81293000 |
| H | 9.16256700 | 0.99731500 | 4.27161000 |
| H | 11.35066200 | 1.64375500 | 3.31827600 |
| H | 11.58269200 | 2.24537700 | 0.94785000 |
| O | 6.00554500 | 1.33759400 | 0.19846900 |
| C | 10.25268100 | 2.69078600 | -1.78289500 |
| C | 11.62319900 | 2.80434100 | -1.40474600 |
| N | 12.74923000 | 2.87471000 | -1.11646300 |
| C | 9.99144400 | 3.10755800 | -3.12178100 |
| N | 9.79939200 | 3.45814500 | -4.21599900 |
| C | 3.54522600 | 1.34477100 | -3.45874500 |
| C | 4.34039400 | 1.36925600 | -4.61506300 |
| C | 5.69199000 | 1.56624500 | -4.29905800 |
| C | 5.97877100 | 1.66946800 | -2.93384300 |
| S | 4.50019300 | 1.51106800 | -1.99907900 |
| H | 6.47392600 | 1.62896300 | -5.04954200 |
| C | 3.84993000 | 1.21144800 | -6.02570700 |

| | | | |
|---|---|---|---|
| H | 3.08262400 | 1.95233100 | -6.27894700 |
| H | 4.67858400 | 1.33305900 | -6.72850800 |
| H | 3.41730000 | 0.21804500 | -6.19048700 |
| C | 2.12741800 | 1.17921000 | -3.31173200 |
| C | 0.07222700 | 0.85511200 | -2.27135400 |
| C | -0.29685600 | 0.62971700 | -3.59536800 |
| S | 1.04969700 | 0.75585600 | -4.66444400 |
| C | -8.77355300 | -2.63591000 | -2.49983000 |
| H | -9.06799900 | -3.53348900 | -3.03118700 |
| C | 7.30176500 | 1.90847700 | -2.48492000 |
| H | 7.96847900 | 2.06909400 | -3.32406600 |
| C | -1.13279600 | 0.69919700 | -1.35971900 |
| O | -1.37547700 | -0.62467200 | -0.82722300 |
| C | -3.65769400 | 11.54109800 | 1.79679800 |
| C | -4.93063700 | 11.48700200 | 2.40309500 |
| C | -5.53771800 | 12.60919200 | 2.94936800 |
| C | -4.84502100 | 13.81998900 | 2.90182800 |
| C | -3.57374900 | 13.88358800 | 2.31664900 |
| C | -2.96805200 | 12.75648500 | 1.75477000 |
| C | -3.32346500 | 10.19860500 | 1.26362600 |
| C | -4.32631300 | 9.27131900 | 1.78650900 |
| C | -5.44128300 | 10.09575500 | 2.36441600 |
| H | -6.52261700 | 12.52628700 | 3.39792100 |
| H | -5.28856600 | 14.71761400 | 3.32187100 |
| H | -3.04249200 | 14.83032500 | 2.29620000 |
| H | -1.98501000 | 12.83977800 | 1.31109400 |
| O | -6.51714200 | 9.68781900 | 2.76630800 |
| C | -2.39549300 | 9.94446800 | 0.27580600 |
| C | -1.49361600 | 10.94059400 | -0.20659300 |
| N | -0.73692800 | 11.73382500 | -0.59904500 |
| C | -2.32032100 | 8.70508500 | -0.42824000 |
| N | -2.24966500 | 7.72457900 | -1.05163900 |
| C | -1.95857900 | 4.89562000 | 1.44889500 |
| C | -1.18913100 | 5.96336700 | 1.89408900 |
| C | -1.97046500 | 7.12586300 | 2.09256100 |
| C | -3.31960400 | 6.96093400 | 1.82563700 |
| S | -3.64758000 | 5.31326600 | 1.34738200 |
| H | -1.55481900 | 8.07147700 | 2.42180300 |
| C | 0.30045700 | 5.91303800 | 2.09092900 |
| H | 0.76103900 | 5.17643800 | 1.42743300 |
| H | 0.74992000 | 6.88927200 | 1.88524700 |
| H | 0.56143900 | 5.63738700 | 3.11995400 |
| C | -1.51474700 | 3.57043700 | 1.04622500 |
| C | -1.68008900 | 2.95272600 | -0.17875400 |

| | | | |
|---|---|---|---|
| C | -1.13961100 | 1.63066100 | -0.18598000 |
| C | -0.53571700 | 1.24864300 | 1.00422800 |
| S | -0.69214400 | 2.53161000 | 2.18882500 |
| C | -2.28721500 | 3.61482900 | -1.38637300 |
| H | -3.30723900 | 3.26049100 | -1.57366000 |
| H | -2.32058800 | 4.70031400 | -1.26638000 |
| H | -1.69987200 | 3.39059000 | -2.28270300 |
| C | -4.38059300 | 7.91641900 | 1.92166300 |
| H | -5.36183200 | 7.54140600 | 2.21605300 |
| C | 8.86997400 | -4.60268600 | 6.07702400 |
| C | 8.24412100 | -5.43658000 | 7.02617800 |
| C | 8.91079600 | -6.48655800 | 7.64015100 |
| C | 10.24058600 | -6.71954000 | 7.28212900 |
| C | 10.86872400 | -5.90734400 | 6.32983100 |
| C | 10.20020300 | -4.84048800 | 5.72198300 |
| C | 7.91172300 | -3.54903400 | 5.66488000 |
| C | 6.61117800 | -3.91052800 | 6.20416200 |
| C | 6.83369200 | -5.01033900 | 7.20397800 |
| H | 8.39643200 | -7.09913800 | 8.37378000 |
| H | 10.79207300 | -7.53493400 | 7.74016900 |
| H | 11.89988700 | -6.10707100 | 6.05466400 |
| H | 10.71382000 | -4.23248700 | 4.98900700 |
| O | 6.00701700 | -5.48666600 | 7.96166300 |
| C | 8.25863600 | -2.34569900 | 5.07824900 |
| C | 9.56034200 | -2.07330500 | 4.56439600 |
| N | 10.60484200 | -1.82305500 | 4.11351900 |
| C | 7.37354000 | -1.23194800 | 5.03921700 |
| N | 6.67895000 | -0.29739100 | 5.01878300 |
| C | 3.09364700 | -1.97616700 | 3.15212900 |
| C | 4.36417400 | -2.10970900 | 2.59897500 |
| C | 5.27851700 | -2.65734600 | 3.52338400 |
| C | 4.74024800 | -2.93682500 | 4.77438700 |
| S | 3.03312900 | -2.52127500 | 4.79977200 |
| H | 6.30662700 | -2.87712100 | 3.26505600 |
| C | 4.73208000 | -1.66948000 | 1.21239800 |
| H | 5.00176500 | -0.60643900 | 1.19403700 |
| H | 5.59340700 | -2.21915300 | 0.82864700 |
| H | 3.90071600 | -1.80509800 | 0.51612600 |
| C | 1.92552600 | -1.45741400 | 2.44667100 |
| C | 1.23555700 | -2.08994200 | 1.43747300 |
| C | 0.28131200 | -1.22819000 | 0.80102000 |
| C | 0.23196400 | 0.05225300 | 1.34531900 |
| S | 1.40714400 | 0.19567400 | 2.64679300 |
| C | 1.48028100 | -3.52065700 | 1.04266800 |

| | | | |
|---|---|---|---|
| H | 1.95415100 | -4.06870300 | 1.86073100 |
| H | 0.54150700 | -4.02191900 | 0.78648000 |
| H | 2.13680200 | -3.59113700 | 0.16796300 |
| C | 5.31377800 | -3.58507100 | 5.90390200 |
| H | 4.60592400 | -3.99882600 | 6.62374900 |
| C | 1.41391400 | 1.21575600 | -2.11379900 |
| H | 1.87352400 | 1.45175700 | -1.16075100 |
| C | -3.56787300 | 0.13530900 | -2.25696700 |
| H | -4.10792100 | 0.09952000 | -1.31889100 |
| C | 1.85653100 | -1.75543600 | -1.91665100 |
| H | 2.47760400 | -1.01609700 | -1.43172800 |
| C | -2.76823100 | -2.95318400 | -0.04209200 |
| H | -3.15323200 | -2.43355500 | 0.82739200 |

**Table S 6. Optimized Cartesian coordinates of DSOCT-(FTIC)6 (B3LYP/6-31G**/EM=GD3BJ)**

**0 1**

| | | | |
|---|---|---|---|
| C | 10.31321400 | -0.48063300 | -2.39287600 |
| C | 9.45259000 | -1.03856900 | -1.42704500 |
| C | 9.84855800 | -1.27040900 | -0.11565400 |
| C | 11.14727400 | -0.92485400 | 0.22362900 |
| C | 12.01344000 | -0.36265700 | -0.72724300 |
| C | 11.62235600 | -0.13695800 | -2.03939300 |
| C | 9.55825300 | -0.30457600 | -3.65280200 |
| C | 8.23416600 | -0.89674600 | -3.45612900 |
| C | 8.12077300 | -1.27709800 | -2.02867300 |
| H | 9.16921700 | -1.67614400 | 0.62493400 |
| H | 12.33372500 | 0.31203500 | -2.71844700 |
| O | 7.13956000 | -1.72616400 | -1.44738900 |
| C | 9.99143600 | 0.41203900 | -4.74982300 |
| C | 11.31041600 | 0.94387500 | -4.85562000 |
| N | 12.38849500 | 1.37252000 | -4.95002900 |
| C | 9.12571800 | 0.75329600 | -5.83227000 |
| N | 8.39681500 | 1.05008800 | -6.69116600 |
| C | 3.57745500 | -2.20829100 | -4.08566200 |
| C | 3.91729300 | -2.18033200 | -5.44341600 |
| C | 5.26754600 | -1.83911000 | -5.61203500 |
| C | 5.98388100 | -1.63271000 | -4.42664700 |

| | | | |
|---|---|---|---|
| S | 4.94120700 | -1.90443800 | -3.04361800 |
| H | 5.72883000 | -1.69530300 | -6.58411000 |
| C | 2.95583200 | -2.40857200 | -6.57707600 |
| H | 2.00802700 | -1.88700600 | -6.40632100 |
| H | 3.38012000 | -2.04900000 | -7.51903300 |
| H | 2.72448800 | -3.47268700 | -6.70686200 |
| C | 2.29202700 | -2.39083400 | -3.46376300 |
| C | 0.51846800 | -2.06623100 | -1.99780000 |
| C | 0.00184100 | -3.08406400 | -2.78857000 |
| S | 1.07817500 | -3.53822200 | -4.06146500 |
| C | -10.65870400 | -2.91521100 | 2.88453800 |
| C | -9.50594900 | -2.11254300 | 2.94505100 |
| C | -9.43543300 | -0.95065900 | 3.70197000 |
| C | -10.56528500 | -0.59096800 | 4.42081000 |
| C | -11.72006300 | -1.38606100 | 4.38191700 |
| C | -11.78786500 | -2.55006200 | 3.62756400 |
| C | -10.40006200 | -4.05359900 | 1.96998200 |
| C | -9.04742900 | -3.88243300 | 1.43562800 |
| C | -8.45384500 | -2.68794900 | 2.07709700 |
| H | -8.53660700 | -0.34439300 | 3.72650200 |
| H | -12.71537700 | -3.10530500 | 3.62717300 |
| O | -7.32828700 | -2.23172500 | 1.92020900 |
| C | -11.29401200 | -5.07246200 | 1.70775800 |
| C | -12.63002500 | -5.06695800 | 2.20657300 |
| N | -13.72862100 | -5.05156500 | 2.59206500 |
| C | -10.98003000 | -6.21501600 | 0.91334300 |
| N | -10.73952100 | -7.15520700 | 0.26926000 |
| C | -4.88174400 | -4.35868100 | -1.12643600 |
| C | -5.65951700 | -5.27389100 | -1.85659800 |
| C | -6.94791700 | -5.38628600 | -1.32077300 |
| C | -7.21334200 | -4.56423200 | -0.22029200 |
| S | -5.79629500 | -3.59894400 | 0.16082400 |
| H | -7.70279100 | -6.05060300 | -1.72843300 |
| C | -5.22338000 | -6.05461500 | -3.06404700 |
| H | -4.34712500 | -6.68039800 | -2.85988100 |
| H | -6.03284300 | -6.70965700 | -3.39737500 |
| H | -4.96786400 | -5.39121200 | -3.89914800 |
| C | -3.51395000 | -3.96198100 | -1.31709600 |
| C | -1.45637600 | -2.90265900 | -1.00041500 |
| C | -1.21476400 | -3.56771700 | -2.19926300 |
| S | -2.56458600 | -4.50023400 | -2.72166700 |
| C | 7.32174900 | -1.16553000 | -4.44706900 |
| H | 7.65484900 | -0.98529700 | -5.46298600 |
| C | -8.47451800 | -4.61491700 | 0.42167100 |

| | | | |
|---|---|---|---|
| H | -9.09086100 | -5.39114700 | -0.01538500 |
| C | -0.46466100 | -1.70726100 | -0.87342400 |
| C | -11.57757800 | -2.23514700 | -0.55564800 |
| C | -10.73164500 | -1.15458900 | -0.24604000 |
| C | -11.06757000 | -0.17363600 | 0.67696500 |
| C | -12.29739900 | -0.29126900 | 1.30226400 |
| C | -13.15399800 | -1.36186500 | 1.00645900 |
| C | -12.82056000 | -2.34053800 | 0.07954200 |
| C | -10.87470200 | -3.12501300 | -1.51107600 |
| C | -9.60697400 | -2.47920300 | -1.85888000 |
| C | -9.47300400 | -1.27086600 | -1.01440000 |
| H | -10.38907300 | 0.63739800 | 0.91538000 |
| H | -13.52072700 | -3.14697700 | -0.08870300 |
| O | -8.52594300 | -0.49766400 | -0.95060600 |
| C | -11.32118800 | -4.37222200 | -1.90076900 |
| C | -12.60116700 | -4.88485300 | -1.53636700 |
| N | -13.64681800 | -5.30833300 | -1.24910300 |
| C | -10.51710100 | -5.28397900 | -2.64751100 |
| N | -9.84066800 | -6.03289300 | -3.22958700 |
| C | -5.30737500 | -1.29160500 | -3.88734000 |
| C | -5.61326000 | -2.36057900 | -4.74411700 |
| C | -6.83919100 | -2.94434800 | -4.39788300 |
| C | -7.50031700 | -2.35720600 | -3.31144600 |
| S | -6.56104300 | -1.00615300 | -2.70434700 |
| H | -7.25251400 | -3.80708100 | -4.91100300 |
| C | -4.73094500 | -2.87655500 | -5.84627700 |
| H | -3.72413400 | -3.10331000 | -5.47695200 |
| H | -5.15044700 | -3.79247300 | -6.27232400 |
| H | -4.62638300 | -2.15223800 | -6.66251600 |
| C | -4.09130600 | -0.53446600 | -3.76399300 |
| C | -2.35182800 | 0.60178100 | -2.71910800 |
| C | -1.80596300 | 0.46030600 | -3.99015900 |
| S | -2.89623600 | -0.34736100 | -5.06839100 |
| C | 9.44352500 | 2.09689800 | 0.13408100 |
| C | 8.24901500 | 1.62569000 | 0.71139300 |
| C | 8.16570600 | 1.20247100 | 2.03204100 |
| C | 9.32921900 | 1.25650400 | 2.78652700 |
| C | 10.53010400 | 1.71468600 | 2.22583600 |
| C | 10.60720000 | 2.15068200 | 0.90907100 |
| C | 9.18094400 | 2.46219300 | -1.27710200 |
| C | 7.78067900 | 2.13893500 | -1.55543900 |
| C | 7.16673100 | 1.65596500 | -0.29731100 |
| H | 7.23634400 | 0.85464600 | 2.46973800 |
| H | 11.56423200 | 2.48960800 | 0.53789200 |

| | | | |
|---|---|---|---|
| O | 6.00255600 | 1.31533900 | -0.11602300 |
| C | 10.10499500 | 3.02836100 | -2.13313700 |
| C | 11.47705500 | 3.19199900 | -1.78085800 |
| N | 12.60433100 | 3.29742400 | -1.50855100 |
| C | 9.79008100 | 3.48665500 | -3.44662600 |
| N | 9.55470400 | 3.86706800 | -4.52217100 |
| C | 3.44123800 | 1.47697200 | -3.75350000 |
| C | 4.22200200 | 1.58766500 | -4.91512000 |
| C | 5.56812400 | 1.81748200 | -4.60294900 |
| C | 5.86754100 | 1.85895000 | -3.23705600 |
| S | 4.40871400 | 1.59583500 | -2.29569400 |
| H | 6.33689200 | 1.95139300 | -5.35739000 |
| C | 3.72385700 | 1.48652200 | -6.32828900 |
| H | 2.92669900 | 2.20963800 | -6.53604200 |
| H | 4.54096600 | 1.67716500 | -7.02918600 |
| H | 3.32845700 | 0.48713300 | -6.54320400 |
| C | 2.02991900 | 1.26614600 | -3.60904600 |
| C | -0.01618000 | 0.85772100 | -2.58280000 |
| C | -0.38049500 | 0.67006800 | -3.91445500 |
| S | 0.96207400 | 0.86618700 | -4.97716200 |
| C | -8.73971600 | -2.86970900 | -2.85139900 |
| H | -9.03636900 | -3.73475300 | -3.43283700 |
| C | 7.18468400 | 2.13275300 | -2.79569500 |
| H | 7.82568800 | 2.37090600 | -3.63606200 |
| C | -1.21590500 | 0.64030100 | -1.67830900 |
| O | -1.42550800 | -0.70984100 | -1.19996800 |
| C | -3.96183500 | 11.28583900 | 1.88578800 |
| C | -5.23546600 | 11.18786900 | 2.48212300 |
| C | -5.87468800 | 12.27536800 | 3.06390700 |
| C | -5.20290200 | 13.48783600 | 3.05341400 |
| C | -3.92909700 | 13.59736400 | 2.47300400 |
| C | -3.29410800 | 12.51539600 | 1.87841700 |
| C | -3.59848500 | 9.96890200 | 1.31207900 |
| C | -4.59067400 | 9.00806800 | 1.79279200 |
| C | -5.72214600 | 9.79053000 | 2.39255200 |
| H | -6.85896200 | 12.18317300 | 3.50975200 |
| H | -2.31448000 | 12.66362200 | 1.44447700 |
| O | -6.79383300 | 9.35550200 | 2.77462400 |
| C | -2.65182200 | 9.76620900 | 0.33020900 |
| C | -1.75866600 | 10.79474500 | -0.09619700 |
| N | -1.01104500 | 11.61965400 | -0.43734900 |
| C | -2.54593200 | 8.55588800 | -0.41869900 |
| N | -2.44999800 | 7.60208700 | -1.07895100 |
| C | -2.14069300 | 4.69564500 | 1.30147900 |

| | | | |
|---|---|---|---|
| C | -1.39756000 | 5.75584500 | 1.80709500 |
| C | -2.20333900 | 6.89331100 | 2.04548100 |
| C | -3.54559400 | 6.71593700 | 1.75122500 |
| S | -3.83574800 | 5.08575200 | 1.19558100 |
| H | -1.81030900 | 7.83019200 | 2.42379100 |
| C | 0.08970400 | 5.72449500 | 2.02460300 |
| H | 0.57700800 | 5.04524900 | 1.32013100 |
| H | 0.51971000 | 6.72199500 | 1.89339300 |
| H | 0.34164700 | 5.38473000 | 3.03648400 |
| C | -1.66603900 | 3.39983800 | 0.84346200 |
| C | -1.81890400 | 2.82938800 | -0.40631700 |
| C | -1.24452100 | 1.52353700 | -0.46884400 |
| C | -0.62734300 | 1.10802900 | 0.70318800 |
| S | -0.81389700 | 2.33577200 | 1.94035600 |
| C | -2.44446000 | 3.52489500 | -1.58562000 |
| H | -3.46082800 | 3.16301800 | -1.77863500 |
| H | -2.49269100 | 4.60486400 | -1.42713500 |
| H | -1.86044500 | 3.34165900 | -2.49331200 |
| C | -4.62414300 | 7.64760900 | 1.87401600 |
| H | -5.60172800 | 7.24474600 | 2.14242300 |
| C | 8.70737500 | -4.51697600 | 6.13199400 |
| C | 8.03921300 | -5.28328500 | 7.10733800 |
| C | 8.67787600 | -6.26204200 | 7.85729700 |
| C | 10.02367200 | -6.47981400 | 7.60389700 |
| C | 10.69950900 | -5.73135200 | 6.62781500 |
| C | 10.06605200 | -4.74207000 | 5.88700000 |
| C | 7.75686800 | -3.52781300 | 5.56837600 |
| C | 6.43196400 | -3.87586700 | 6.06206100 |
| C | 6.61306400 | -4.88276400 | 7.16154500 |
| H | 8.14905300 | -6.83517300 | 8.61090200 |
| H | 10.64246300 | -4.19665700 | 5.15213300 |
| O | 5.75309100 | -5.31996000 | 7.90538900 |
| C | 8.11948400 | -2.38123100 | 4.88869800 |
| C | 9.44839200 | -2.15401100 | 4.42304700 |
| N | 10.52280500 | -1.98640200 | 4.00727500 |
| C | 7.22112700 | -1.29903300 | 4.67242400 |
| N | 6.50547200 | -0.39894900 | 4.49104400 |
| C | 3.05310900 | -2.12124000 | 2.75405900 |
| C | 4.33224800 | -2.32275800 | 2.24464300 |
| C | 5.20271900 | -2.84986800 | 3.22456200 |
| C | 4.62323300 | -3.03707200 | 4.47324900 |
| S | 2.93109800 | -2.57530500 | 4.42636200 |
| H | 6.23236300 | -3.10979400 | 3.01298800 |
| C | 4.76542400 | -1.93927100 | 0.85748300 |

| | | | |
|---|---|---|---|
| H | 5.11128400 | -0.89822400 | 0.83017500 |
| H | 5.59448400 | -2.55839700 | 0.50627400 |
| H | 3.94649600 | -2.02663600 | 0.13960500 |
| C | 1.91124000 | -1.58860500 | 2.02114400 |
| C | 1.23443800 | -2.19764900 | 0.98874600 |
| C | 0.25425700 | -1.33594400 | 0.39363400 |
| C | 0.17129600 | -0.08138800 | 0.99200900 |
| S | 1.34719100 | 0.03905600 | 2.29422200 |
| C | 1.49938100 | -3.61076600 | 0.54679000 |
| H | 2.00134300 | -4.17165400 | 1.33897200 |
| H | 0.56518700 | -4.12339100 | 0.29590100 |
| H | 2.13792300 | -3.64463300 | -0.34316500 |
| C | 5.14931000 | -3.60187000 | 5.67014700 |
| H | 4.41085100 | -3.97323800 | 6.38240300 |
| C | 1.31667700 | 1.24238500 | -2.41077400 |
| H | 1.77020700 | 1.45631400 | -1.44959000 |
| C | -3.63541400 | 0.05349600 | -2.59177600 |
| H | -4.17039000 | -0.02507900 | -1.65351300 |
| C | 1.83653300 | -1.72224800 | -2.33530200 |
| H | 2.44249100 | -0.99229800 | -1.81779400 |
| C | -2.75679700 | -3.11151100 | -0.51176900 |
| H | -3.15421200 | -2.64285100 | 0.38086600 |
| F | 10.70785900 | -7.40901900 | 8.27803600 |
| F | 11.99327200 | -5.99413800 | 6.41898200 |
| F | -3.32552200 | 14.78924200 | 2.50504800 |
| F | -5.74727000 | 14.57972300 | 3.59835200 |
| F | 11.61857300 | 1.73631000 | 2.99689100 |
| F | 9.33804900 | 0.87561500 | 4.06831000 |
| F | 13.24006100 | -0.02112500 | -0.32900500 |
| F | 11.59684600 | -1.07550600 | 1.47026600 |
| F | -10.58167900 | 0.52004200 | 5.16344800 |
| F | -12.78002500 | -0.99174600 | 5.09260300 |
| F | -12.67797500 | 0.58663600 | 2.23892300 |
| F | -14.30844900 | -1.44063900 | 1.67356900 |

**Table S 7. Crystal data and structure refinement for DSOCT-$Br_6$**

| | | |
|---|---|---|
| Identification code | yl2401a (DSOCT-$Br_6$) | |
| Empirical formula | $C_{38} H_{30} Br_6 O S_6$ | |
| Formula weight | 1174.44 | |
| Temperature | 296(2) K | |
| Wavelength | 0.71073 Å | |
| Crystal system | Triclinic | |
| Space group | P-1 | |
| Unit cell dimensions | a = 10.4162(5) Å | □= 62.453(2)°. |
| | b = 15.0277(7) Å | □= 86.778(2)°. |
| | c = 15.2242(7) Å | □ = 86.042(2)°. |
| Volume | 2107.07(17) $Å^3$ | |
| Z | 2 | |
| Density (calculated) | 1.851 $Mg/m^3$ | |
| Absorption coefficient | 6.044 $mm^{-1}$ | |
| F(000) | 1144 | |
| Crystal size | 0.300 x 0.250 x 0.060 $mm^3$ | |
| Theta range for data collection | 2.507 to 26.000°. | |
| Index ranges | -12<=h<=12, -18<=k<=18, -18<=l<=18 | |
| Reflections collected | 84277 | |
| Independent reflections | 8279 [R(int) = 0.0499] | |
| Completeness to theta = 25.242° | 99.8 % | |
| Absorption correction | Multi-scan (Bruker SADABS) | |
| Max. and min. transmission | 0.7461 and 0.4591 | |
| Refinement method | Full-matrix least-squares on $F^2$ | |
| Data / restraints / parameters | 8279 / 32 / 506 | |
| Goodness-of-fit on $F^2$ | 1.032 | |
| Final R indices [I>2sigma(I)] | R1 = 0.0299, wR2 = 0.0720 | |
| R indices (all data) | R1 = 0.0385, wR2 = 0.0763 | |
| Extinction coefficient | n/a | |
| Largest diff. peak and hole | 0.532 and -0.960 e.$Å^{-3}$ | |

**Table S 8. Atomic coordinates ( x $10^4$) and equivalent isotropic displacement parameters ($Å^2$x $10^3$) ForDSOCT-$Br_6$. U(eq) is defined as one third of the trace of the orthogonalized $U^{ij}$ tensor.**

| | x | y | z | U(eq) |
|---|---|---|---|---|
| Br(1) | 1637(1) | -39(1) | 9380(1) | 73(1) |
| Br(2) | 8567(1) | 2841(1) | 7423(1) | 65(1) |
| Br(3) | 437(1) | 4157(1) | 3853(1) | 66(1) |
| Br(4) | 5812(1) | 1928(1) | 10978(1) | 63(1) |
| Br(5) | 4781(1) | 8746(1) | 5357(1) | 80(1) |
| Br(6) | -1581(1) | 6043(1) | 9038(1) | 41(1) |
| S(1) | 861(1) | 2034(1) | 9292(1) | 39(1) |
| S(2) | 3332(1) | 3987(1) | 4332(1) | 46(1) |
| S(3) | 6596(1) | 3472(1) | 5761(1) | 44(1) |
| S(4) | 6056(1) | 4188(1) | 9578(1) | 39(1) |
| S(5) | 5663(1) | 6939(1) | 7323(1) | 42(1) |
| S(6) | -489(1) | 4166(1) | 8859(1) | 33(1) |
| O(1) | 3012(2) | 4728(1) | 6962(1) | 28(1) |
| C(1A) | 7810(40) | -1110(30) | 7900(40) | 126(6) |
| C(2A) | 8260(20) | -130(30) | 7810(40) | 111(4) |
| C(1B) | 7700(80) | -410(60) | 6790(50) | 126(6) |
| C(2B) | 7560(60) | -680(40) | 7890(50) | 111(4) |
| C(1C) | 7462(14) | -1035(12) | 7365(18) | 126(6) |
| C(2C) | 7940(10) | -278(13) | 7657(12) | 111(4) |
| C(3) | 6983(5) | 40(5) | 8270(6) | 102(2) |
| C(4) | 5786(4) | 562(3) | 7704(3) | 68(1) |
| C(5) | 4799(3) | 912(3) | 8269(3) | 50(1) |
| C(6) | 3621(3) | 1424(2) | 7656(2) | 40(1) |
| C(7) | 2680(3) | 1894(2) | 8128(2) | 33(1) |
| C(8) | 2526(2) | 2942(2) | 7886(2) | 26(1) |
| C(9) | 1547(2) | 3133(2) | 8434(2) | 27(1) |
| C(10) | 1072(2) | 4063(2) | 8450(2) | 27(1) |
| C(11) | 1720(2) | 4913(2) | 8210(2) | 26(1) |
| C(12) | 974(3) | 5661(2) | 8377(2) | 28(1) |
| C(13) | 1391(3) | 6654(2) | 8252(2) | 33(1) |
| C(14) | 940(3) | 7565(2) | 7303(2) | 38(1) |
| C(15) | 1472(4) | 8530(2) | 7186(3) | 51(1) |
| C(16) | 1145(5) | 9449(3) | 6239(4) | 65(1) |

| | | | | |
|---|---|---|---|---|
| C(17A) | 1530(110) | 10480(30) | 6060(100) | 87(3) |
| C(18A) | 650(80) | 11380(40) | 5410(70) | 139(6) |
| C(17B) | 1670(20) | 10375(5) | 6237(15) | 87(3) |
| C(18B) | 1383(18) | 11327(7) | 5280(10) | 139(6) |
| C(17C) | 1860(60) | 10420(30) | 5790(50) | 87(3) |
| C(18C) | 1000(50) | 11030(30) | 6180(50) | 139(6) |
| C(19) | 1837(3) | 1345(2) | 8861(3) | 42(1) |
| C(20) | 3258(2) | 3750(2) | 7028(2) | 27(1) |
| C(21) | 2794(3) | 3871(2) | 6037(2) | 30(1) |
| C(22) | 1585(3) | 3988(2) | 5616(2) | 35(1) |
| C(23) | 1741(3) | 4057(2) | 4698(2) | 41(1) |
| C(24) | 3813(3) | 3855(2) | 5440(2) | 35(1) |
| C(25) | 4994(3) | 3669(2) | 5967(2) | 34(1) |
| C(26) | 6896(3) | 3222(2) | 6958(2) | 41(1) |
| C(27) | 5832(3) | 3302(2) | 7482(2) | 34(1) |
| C(28) | 4721(3) | 3552(2) | 6908(2) | 29(1) |
| C(29) | 3096(2) | 4934(2) | 7801(2) | 26(1) |
| C(30) | 3761(3) | 5927(2) | 7351(2) | 29(1) |
| C(31) | 4829(3) | 5860(2) | 7862(2) | 31(1) |
| C(32) | 5003(3) | 4846(2) | 8646(2) | 30(1) |
| C(33) | 5163(3) | 3141(2) | 9969(2) | 35(1) |
| C(34) | 4106(3) | 3287(2) | 9429(2) | 31(1) |
| C(35) | 4030(2) | 4272(2) | 8638(2) | 26(1) |
| C(36) | 4570(3) | 7453(2) | 6382(2) | 43(1) |
| C(37) | 3621(3) | 6838(2) | 6472(2) | 35(1) |
| C(38) | -229(3) | 5335(2) | 8723(2) | 31(1) |

**Table S 9. Bond lengths [Å] and angles [°] for DSOCT-$Br_6$**

| | |
|---|---|
| Br(1)-C(19) | 1.875(3) |
| Br(2)-C(26) | 1.867(3) |
| Br(3)-C(23) | 1.874(3) |
| Br(4)-C(33) | 1.870(3) |
| Br(5)-C(36) | 1.865(3) |
| Br(6)-C(38) | 1.881(3) |
| S(1)-C(19) | 1.716(3) |
| S(1)-C(9) | 1.735(3) |
| S(2)-C(24) | 1.706(3) |
| S(2)-C(23) | 1.730(4) |
| S(3)-C(25) | 1.712(3) |
| S(3)-C(26) | 1.726(3) |
| S(4)-C(32) | 1.708(3) |
| S(4)-C(33) | 1.722(3) |
| S(5)-C(31) | 1.714(3) |
| S(5)-C(36) | 1.727(4) |
| S(6)-C(38) | 1.711(3) |
| S(6)-C(10) | 1.730(3) |
| O(1)-C(20) | 1.432(3) |
| O(1)-C(29) | 1.455(3) |
| C(1A)-C(2A) | 1.523(11) |
| C(1A)-H(1AA) | 0.9600 |
| C(1A)-H(1AB) | 0.9600 |
| C(1A)-H(1AC) | 0.9600 |
| C(2A)-C(3) | 1.526(10) |
| C(2A)-H(2AA) | 0.9700 |
| C(2A)-H(2AB) | 0.9700 |
| C(1B)-C(2B) | 1.524(11) |
| C(1B)-H(1BA) | 0.9600 |
| C(1B)-H(1BB) | 0.9600 |
| C(1B)-H(1BC) | 0.9600 |
| C(2B)-C(3) | 1.526(11) |
| C(2B)-H(2BA) | 0.9700 |
| C(2B)-H(2BB) | 0.9700 |
| C(1C)-C(2C) | 1.520(9) |
| C(1C)-H(1CA) | 0.9600 |

| | |
|---|---|
| C(1C)-H(1CB) | 0.9600 |
| C(1C)-H(1CC) | 0.9600 |
| C(2C)-C(3) | 1.528(8) |
| C(2C)-H(2CA) | 0.9700 |
| C(2C)-H(2CB) | 0.9700 |
| C(3)-C(4) | 1.508(7) |
| C(3)-H(3A) | 1.13(6) |
| C(3)-H(3B) | 0.99(5) |
| C(4)-C(5) | 1.522(5) |
| C(4)-H(4A) | 0.9700 |
| C(4)-H(4B) | 0.9700 |
| C(5)-C(6) | 1.518(5) |
| C(5)-H(5A) | 0.9700 |
| C(5)-H(5B) | 0.9700 |
| C(6)-C(7) | 1.506(4) |
| C(6)-H(6A) | 0.9700 |
| C(6)-H(6B) | 0.9700 |
| C(7)-C(19) | 1.355(4) |
| C(7)-C(8) | 1.441(4) |
| C(8)-C(9) | 1.381(4) |
| C(8)-C(20) | 1.520(4) |
| C(9)-C(10) | 1.460(4) |
| C(10)-C(11) | 1.371(4) |
| C(11)-C(12) | 1.433(4) |
| C(11)-C(29) | 1.527(3) |
| C(12)-C(38) | 1.363(4) |
| C(12)-C(13) | 1.507(4) |
| C(13)-C(14) | 1.530(4) |
| C(13)-H(13A) | 0.9700 |
| C(13)-H(13B) | 0.9700 |
| C(14)-C(15) | 1.516(4) |
| C(14)-H(14A) | 0.9700 |
| C(14)-H(14B) | 0.9700 |
| C(15)-C(16) | 1.500(5) |
| C(15)-H(15A) | 0.9700 |
| C(15)-H(15B) | 0.9700 |
| C(16)-C(17C) | 1.525(10) |
| C(16)-C(17A) | 1.527(10) |

| | |
|---|---|
| C(16)-C(17B) | 1.529(6) |
| C(16)-H(16B) | 0.94(5) |
| C(16)-H(16A) | 0.95(4) |
| C(17A)-C(18A) | 1.527(10) |
| C(17A)-H(17A) | 0.9700 |
| C(17A)-H(17B) | 0.9700 |
| C(18A)-H(18A) | 0.9600 |
| C(18A)-H(18B) | 0.9600 |
| C(18A)-H(18C) | 0.9600 |
| C(17B)-C(18B) | 1.524(9) |
| C(17B)-H(17C) | 0.9700 |
| C(17B)-H(17D) | 0.9700 |
| C(18B)-H(18D) | 0.9600 |
| C(18B)-H(18E) | 0.9600 |
| C(18B)-H(18F) | 0.9600 |
| C(17C)-C(18C) | 1.525(11) |
| C(17C)-H(17E) | 0.9700 |
| C(17C)-H(17F) | 0.9700 |
| C(18C)-H(18G) | 0.9600 |
| C(18C)-H(18H) | 0.9600 |
| C(18C)-H(18I) | 0.9600 |
| C(20)-C(21) | 1.535(4) |
| C(20)-C(28) | 1.549(4) |
| C(21)-C(24) | 1.363(4) |
| C(21)-C(22) | 1.408(4) |
| C(22)-C(23) | 1.354(4) |
| C(22)-H(22A) | 0.9300 |
| C(24)-C(25) | 1.446(4) |
| C(25)-C(28) | 1.375(4) |
| C(26)-C(27) | 1.361(4) |
| C(27)-C(28) | 1.412(4) |
| C(27)-H(27A) | 0.9300 |
| C(29)-C(30) | 1.523(4) |
| C(29)-C(35) | 1.547(4) |
| C(30)-C(31) | 1.365(4) |
| C(30)-C(37) | 1.409(4) |
| C(31)-C(32) | 1.444(4) |
| C(32)-C(35) | 1.379(4) |

| | |
|---|---|
| C(33)-C(34) | 1.356(4) |
| C(34)-C(35) | 1.413(4) |
| C(34)-H(34A) | 0.9300 |
| C(36)-C(37) | 1.357(4) |
| C(37)-H(37A) | 0.9300 |
| | |
| C(19)-S(1)-C(9) | 90.77(14) |
| C(24)-S(2)-C(23) | 89.94(14) |
| C(25)-S(3)-C(26) | 89.62(14) |
| C(32)-S(4)-C(33) | 89.70(14) |
| C(31)-S(5)-C(36) | 89.50(15) |
| C(38)-S(6)-C(10) | 90.53(13) |
| C(20)-O(1)-C(29) | 123.28(19) |
| C(2A)-C(1A)-H(1AA) | 109.5 |
| C(2A)-C(1A)-H(1AB) | 109.5 |
| H(1AA)-C(1A)-H(1AB) | 109.5 |
| C(2A)-C(1A)-H(1AC) | 109.5 |
| H(1AA)-C(1A)-H(1AC) | 109.5 |
| H(1AB)-C(1A)-H(1AC) | 109.5 |
| C(1A)-C(2A)-C(3) | 90.7(19) |
| C(1A)-C(2A)-H(2AA) | 113.5 |
| C(3)-C(2A)-H(2AA) | 113.5 |
| C(1A)-C(2A)-H(2AB) | 113.5 |
| C(3)-C(2A)-H(2AB) | 113.5 |
| H(2AA)-C(2A)-H(2AB) | 110.8 |
| C(2B)-C(1B)-H(1BA) | 109.5 |
| C(2B)-C(1B)-H(1BB) | 109.5 |
| H(1BA)-C(1B)-H(1BB) | 109.5 |
| C(2B)-C(1B)-H(1BC) | 109.5 |
| H(1BA)-C(1B)-H(1BC) | 109.5 |
| H(1BB)-C(1B)-H(1BC) | 109.5 |
| C(1B)-C(2B)-C(3) | 124(5) |
| C(1B)-C(2B)-H(2BA) | 106.4 |
| C(3)-C(2B)-H(2BA) | 106.4 |
| C(1B)-C(2B)-H(2BB) | 106.4 |
| C(3)-C(2B)-H(2BB) | 106.4 |
| H(2BA)-C(2B)-H(2BB) | 106.5 |
| C(2C)-C(1C)-H(1CA) | 109.5 |

| | |
|---|---|
| C(2C)-C(1C)-H(1CB) | 109.5 |
| H(1CA)-C(1C)-H(1CB) | 109.5 |
| C(2C)-C(1C)-H(1CC) | 109.5 |
| H(1CA)-C(1C)-H(1CC) | 109.5 |
| H(1CB)-C(1C)-H(1CC) | 109.5 |
| C(1C)-C(2C)-C(3) | 115.3(11) |
| C(1C)-C(2C)-H(2CA) | 108.5 |
| C(3)-C(2C)-H(2CA) | 108.5 |
| C(1C)-C(2C)-H(2CB) | 108.5 |
| C(3)-C(2C)-H(2CB) | 108.5 |
| H(2CA)-C(2C)-H(2CB) | 107.5 |
| C(4)-C(3)-C(2A) | 125(2) |
| C(4)-C(3)-C(2B) | 109(3) |
| C(4)-C(3)-C(2C) | 111.6(8) |
| C(4)-C(3)-H(3A) | 101(3) |
| C(2A)-C(3)-H(3A) | 117(3) |
| C(2B)-C(3)-H(3A) | 87(4) |
| C(2C)-C(3)-H(3A) | 111(3) |
| C(4)-C(3)-H(3B) | 107(3) |
| C(2A)-C(3)-H(3B) | 98(3) |
| C(2B)-C(3)-H(3B) | 138(4) |
| C(2C)-C(3)-H(3B) | 117(3) |
| H(3A)-C(3)-H(3B) | 107(4) |
| C(3)-C(4)-C(5) | 114.3(4) |
| C(3)-C(4)-H(4A) | 108.7 |
| C(5)-C(4)-H(4A) | 108.7 |
| C(3)-C(4)-H(4B) | 108.7 |
| C(5)-C(4)-H(4B) | 108.7 |
| H(4A)-C(4)-H(4B) | 107.6 |
| C(6)-C(5)-C(4) | 111.3(3) |
| C(6)-C(5)-H(5A) | 109.4 |
| C(4)-C(5)-H(5A) | 109.4 |
| C(6)-C(5)-H(5B) | 109.4 |
| C(4)-C(5)-H(5B) | 109.4 |
| H(5A)-C(5)-H(5B) | 108.0 |
| C(7)-C(6)-C(5) | 113.7(3) |
| C(7)-C(6)-H(6A) | 108.8 |
| C(5)-C(6)-H(6A) | 108.8 |

| | |
|---|---|
| C(7)-C(6)-H(6B) | 108.8 |
| C(5)-C(6)-H(6B) | 108.8 |
| H(6A)-C(6)-H(6B) | 107.7 |
| C(19)-C(7)-C(8) | 110.9(3) |
| C(19)-C(7)-C(6) | 122.1(3) |
| C(8)-C(7)-C(6) | 127.0(3) |
| C(9)-C(8)-C(7) | 112.7(2) |
| C(9)-C(8)-C(20) | 124.3(2) |
| C(7)-C(8)-C(20) | 122.6(2) |
| C(8)-C(9)-C(10) | 131.6(2) |
| C(8)-C(9)-S(1) | 111.4(2) |
| C(10)-C(9)-S(1) | 116.94(19) |
| C(11)-C(10)-C(9) | 128.7(2) |
| C(11)-C(10)-S(6) | 111.4(2) |
| C(9)-C(10)-S(6) | 119.9(2) |
| C(10)-C(11)-C(12) | 113.6(2) |
| C(10)-C(11)-C(29) | 116.7(2) |
| C(12)-C(11)-C(29) | 129.7(2) |
| C(38)-C(12)-C(11) | 109.8(2) |
| C(38)-C(12)-C(13) | 121.9(2) |
| C(11)-C(12)-C(13) | 128.3(2) |
| C(12)-C(13)-C(14) | 114.6(2) |
| C(12)-C(13)-H(13A) | 108.6 |
| C(14)-C(13)-H(13A) | 108.6 |
| C(12)-C(13)-H(13B) | 108.6 |
| C(14)-C(13)-H(13B) | 108.6 |
| H(13A)-C(13)-H(13B) | 107.6 |
| C(15)-C(14)-C(13) | 111.9(3) |
| C(15)-C(14)-H(14A) | 109.2 |
| C(13)-C(14)-H(14A) | 109.2 |
| C(15)-C(14)-H(14B) | 109.2 |
| C(13)-C(14)-H(14B) | 109.2 |
| H(14A)-C(14)-H(14B) | 107.9 |
| C(16)-C(15)-C(14) | 115.2(3) |
| C(16)-C(15)-H(15A) | 108.5 |
| C(14)-C(15)-H(15A) | 108.5 |
| C(16)-C(15)-H(15B) | 108.5 |
| C(14)-C(15)-H(15B) | 108.5 |

| | |
|---|---|
| H(15A)-C(15)-H(15B) | 107.5 |
| C(15)-C(16)-C(17C) | 126(3) |
| C(15)-C(16)-C(17A) | 121(4) |
| C(15)-C(16)-C(17B) | 110.0(6) |
| C(15)-C(16)-H(16B) | 110(3) |
| C(17C)-C(16)-H(16B) | 115(4) |
| C(17A)-C(16)-H(16B) | 104(4) |
| C(17B)-C(16)-H(16B) | 111(3) |
| C(15)-C(16)-H(16A) | 110(3) |
| C(17C)-C(16)-H(16A) | 87(3) |
| C(17A)-C(16)-H(16A) | 107(7) |
| C(17B)-C(16)-H(16A) | 112(3) |
| H(16B)-C(16)-H(16A) | 103(4) |
| C(18A)-C(17A)-C(16) | 117(4) |
| C(18A)-C(17A)-H(17A) | 108.0 |
| C(16)-C(17A)-H(17A) | 108.0 |
| C(18A)-C(17A)-H(17B) | 108.0 |
| C(16)-C(17A)-H(17B) | 108.0 |
| H(17A)-C(17A)-H(17B) | 107.3 |
| C(17A)-C(18A)-H(18A) | 109.5 |
| C(17A)-C(18A)-H(18B) | 109.5 |
| H(18A)-C(18A)-H(18B) | 109.5 |
| C(17A)-C(18A)-H(18C) | 109.5 |
| H(18A)-C(18A)-H(18C) | 109.5 |
| H(18B)-C(18A)-H(18C) | 109.5 |
| C(18B)-C(17B)-C(16) | 112.0(9) |
| C(18B)-C(17B)-H(17C) | 109.2 |
| C(16)-C(17B)-H(17C) | 109.2 |
| C(18B)-C(17B)-H(17D) | 109.2 |
| C(16)-C(17B)-H(17D) | 109.2 |
| H(17C)-C(17B)-H(17D) | 107.9 |
| C(17B)-C(18B)-H(18D) | 109.5 |
| C(17B)-C(18B)-H(18E) | 109.5 |
| H(18D)-C(18B)-H(18E) | 109.5 |
| C(17B)-C(18B)-H(18F) | 109.5 |
| H(18D)-C(18B)-H(18F) | 109.5 |
| H(18E)-C(18B)-H(18F) | 109.5 |
| C(16)-C(17C)-C(18C) | 100(3) |

| | |
|---|---|
| C(16)-C(17C)-H(17E) | 111.9 |
| C(18C)-C(17C)-H(17E) | 111.9 |
| C(16)-C(17C)-H(17F) | 111.9 |
| C(18C)-C(17C)-H(17F) | 111.9 |
| H(17E)-C(17C)-H(17F) | 109.6 |
| C(17C)-C(18C)-H(18G) | 109.5 |
| C(17C)-C(18C)-H(18H) | 109.5 |
| H(18G)-C(18C)-H(18H) | 109.5 |
| C(17C)-C(18C)-H(18I) | 109.5 |
| H(18G)-C(18C)-H(18I) | 109.5 |
| H(18H)-C(18C)-H(18I) | 109.5 |
| C(7)-C(19)-S(1) | 114.2(2) |
| C(7)-C(19)-Br(1) | 126.0(2) |
| S(1)-C(19)-Br(1) | 119.78(18) |
| O(1)-C(20)-C(8) | 112.9(2) |
| O(1)-C(20)-C(21) | 103.6(2) |
| C(8)-C(20)-C(21) | 110.2(2) |
| O(1)-C(20)-C(28) | 110.2(2) |
| C(8)-C(20)-C(28) | 118.1(2) |
| C(21)-C(20)-C(28) | 100.1(2) |
| C(24)-C(21)-C(22) | 114.1(3) |
| C(24)-C(21)-C(20) | 110.7(2) |
| C(22)-C(21)-C(20) | 135.2(2) |
| C(23)-C(22)-C(21) | 110.0(3) |
| C(23)-C(22)-H(22A) | 125.0 |
| C(21)-C(22)-H(22A) | 125.0 |
| C(22)-C(23)-S(2) | 114.0(2) |
| C(22)-C(23)-Br(3) | 126.9(3) |
| S(2)-C(23)-Br(3) | 119.11(17) |
| C(21)-C(24)-C(25) | 109.4(2) |
| C(21)-C(24)-S(2) | 112.0(2) |
| C(25)-C(24)-S(2) | 138.5(2) |
| C(28)-C(25)-C(24) | 109.8(2) |
| C(28)-C(25)-S(3) | 112.9(2) |
| C(24)-C(25)-S(3) | 137.0(2) |
| C(27)-C(26)-S(3) | 114.1(2) |
| C(27)-C(26)-Br(2) | 126.6(2) |
| S(3)-C(26)-Br(2) | 119.28(17) |

| | |
|---|---|
| C(26)-C(27)-C(28) | 110.8(3) |
| C(26)-C(27)-H(27A) | 124.6 |
| C(28)-C(27)-H(27A) | 124.6 |
| C(25)-C(28)-C(27) | 112.5(2) |
| C(25)-C(28)-C(20) | 109.5(2) |
| C(27)-C(28)-C(20) | 138.0(2) |
| O(1)-C(29)-C(30) | 103.1(2) |
| O(1)-C(29)-C(11) | 105.6(2) |
| C(30)-C(29)-C(11) | 118.2(2) |
| O(1)-C(29)-C(35) | 119.0(2) |
| C(30)-C(29)-C(35) | 100.4(2) |
| C(11)-C(29)-C(35) | 111.0(2) |
| C(31)-C(30)-C(37) | 113.6(3) |
| C(31)-C(30)-C(29) | 111.2(2) |
| C(37)-C(30)-C(29) | 134.6(3) |
| C(30)-C(31)-C(32) | 109.1(2) |
| C(30)-C(31)-S(5) | 112.4(2) |
| C(32)-C(31)-S(5) | 138.1(2) |
| C(35)-C(32)-C(31) | 109.9(2) |
| C(35)-C(32)-S(4) | 112.8(2) |
| C(31)-C(32)-S(4) | 137.3(2) |
| C(34)-C(33)-S(4) | 114.2(2) |
| C(34)-C(33)-Br(4) | 127.3(2) |
| S(4)-C(33)-Br(4) | 118.18(16) |
| C(33)-C(34)-C(35) | 110.8(3) |
| C(33)-C(34)-H(34A) | 124.6 |
| C(35)-C(34)-H(34A) | 124.6 |
| C(32)-C(35)-C(34) | 112.3(2) |
| C(32)-C(35)-C(29) | 109.4(2) |
| C(34)-C(35)-C(29) | 138.0(2) |
| C(37)-C(36)-S(5) | 114.4(2) |
| C(37)-C(36)-Br(5) | 126.1(3) |
| S(5)-C(36)-Br(5) | 119.46(19) |
| C(36)-C(37)-C(30) | 110.0(3) |
| C(36)-C(37)-H(37A) | 125.0 |
| C(30)-C(37)-H(37A) | 125.0 |
| C(12)-C(38)-S(6) | 114.6(2) |
| C(12)-C(38)-Br(6) | 126.6(2) |

| | |
|---|---|
| S(6)-C(38)-Br(6) | 118.79(16) |

Symmetry transformations used to generate equivalent atoms:

**Table S 10. Hydrogen bonds for DSOCT-$Br_6$. [Å and °].**

| D-H...A | d(D-H) | d(H...A) | d(D...A) | <(DHA) |
|---|---|---|---|---|
| C(22)-H(22A)…Br(3)#1 | 0.93 | 3.11 | 3.735(3) | 126.4 |

Symmetry transformations used to generate equivalent atoms:
#1 -x,-y+1,-z+1

**Table S 11. Anisotropic displacement parameters ($Å^2$x $10^3$) for yl2401a. The anisotropic displacement factor exponent takes the form: $-2\pi^2[ h^2 a^{*2}U^{11} + ... + 2 h k a^* b^* U^{12} ]$**

| | $U^{11}$ | $U^{22}$ | $U^{33}$ | $U^{23}$ | $U^{13}$ | $U^{12}$ |
|---|---|---|---|---|---|---|
| Br(1) | 75(1) | 30(1) | 105(1) | -25(1) | 34(1) | -13(1) |
| Br(2) | 28(1) | 89(1) | 92(1) | -54(1) | -8(1) | 4(1) |
| Br(3) | 79(1) | 70(1) | 54(1) | -31(1) | -34(1) | 6(1) |
| Br(4) | 68(1) | 47(1) | 58(1) | -11(1) | -25(1) | 11(1) |
| Br(5) | 98(1) | 37(1) | 75(1) | 1(1) | 7(1) | -21(1) |
| Br(6) | 32(1) | 46(1) | 51(1) | -29(1) | 0(1) | 8(1) |
| S(1) | 34(1) | 30(1) | 46(1) | -13(1) | 12(1) | -5(1) |
| S(2) | 58(1) | 55(1) | 30(1) | -25(1) | -3(1) | 6(1) |
| S(3) | 34(1) | 57(1) | 45(1) | -27(1) | 11(1) | -2(1) |
| S(4) | 32(1) | 44(1) | 43(1) | -22(1) | -10(1) | 0(1) |
| S(5) | 45(1) | 35(1) | 51(1) | -22(1) | 6(1) | -14(1) |
| S(6) | 25(1) | 36(1) | 38(1) | -19(1) | 5(1) | -2(1) |
| O(1) | 35(1) | 27(1) | 23(1) | -12(1) | 0(1) | 0(1) |
| C(1A) | 81(8) | 109(8) | 230(20) | -120(12) | 17(9) | 5(6) |
| C(2A) | 28(6) | 95(9) | 213(11) | -76(6) | 14(7) | -7(6) |
| C(1B) | 81(8) | 109(8) | 230(20) | -120(12) | 17(9) | 5(6) |
| C(2B) | 28(6) | 95(9) | 213(11) | -76(6) | 14(7) | -7(6) |
| C(1C) | 81(8) | 109(8) | 230(20) | -120(12) | 17(9) | 5(6) |
| C(2C) | 28(6) | 95(9) | 213(11) | -76(6) | 14(7) | -7(6) |
| C(3) | 54(3) | 97(4) | 154(6) | -58(5) | -9(3) | 18(3) |
| C(4) | 54(2) | 52(2) | 85(3) | -25(2) | 13(2) | 12(2) |
| C(5) | 46(2) | 36(2) | 62(2) | -19(2) | 3(2) | 3(1) |
| C(6) | 44(2) | 32(2) | 50(2) | -24(1) | 7(1) | -2(1) |
| C(7) | 32(1) | 29(1) | 38(2) | -16(1) | 0(1) | 1(1) |
| C(8) | 24(1) | 27(1) | 26(1) | -12(1) | -3(1) | -1(1) |
| C(9) | 25(1) | 27(1) | 28(1) | -12(1) | 1(1) | -2(1) |
| C(10) | 22(1) | 32(1) | 26(1) | -13(1) | 1(1) | 0(1) |
| C(11) | 25(1) | 28(1) | 22(1) | -11(1) | -2(1) | 1(1) |
| C(12) | 29(1) | 28(1) | 24(1) | -11(1) | -4(1) | 3(1) |
| C(13) | 34(2) | 33(2) | 35(2) | -19(1) | -3(1) | 1(1) |
| C(14) | 36(2) | 31(2) | 47(2) | -18(1) | -4(1) | 2(1) |
| C(15) | 56(2) | 37(2) | 61(2) | -23(2) | -10(2) | -1(2) |

| | | | | | | |
|---|---|---|---|---|---|---|
| C(16) | 70(3) | 32(2) | 80(3) | -13(2) | -20(2) | -6(2) |
| C(17A) | 113(7) | 36(3) | 98(10) | -14(4) | -32(5) | -13(3) |
| C(18A) | 158(13) | 37(3) | 169(10) | 9(4) | -64(10) | -32(6) |
| C(17B) | 113(7) | 36(3) | 98(10) | -14(4) | -32(5) | -13(3) |
| C(18B) | 158(13) | 37(3) | 169(10) | 9(4) | -64(10) | -32(6) |
| C(17C) | 113(7) | 36(3) | 98(10) | -14(4) | -32(5) | -13(3) |
| C(18C) | 158(13) | 37(3) | 169(10) | 9(4) | -64(10) | -32(6) |
| C(19) | 41(2) | 27(2) | 54(2) | -16(1) | 7(1) | -3(1) |
| C(20) | 27(1) | 29(1) | 27(1) | -15(1) | -1(1) | 1(1) |
| C(21) | 33(1) | 30(1) | 27(1) | -14(1) | 0(1) | -2(1) |
| C(22) | 35(2) | 35(2) | 34(2) | -16(1) | -4(1) | 0(1) |
| C(23) | 51(2) | 38(2) | 36(2) | -19(1) | -12(1) | 2(1) |
| C(24) | 40(2) | 37(2) | 28(1) | -16(1) | 1(1) | 0(1) |
| C(25) | 32(1) | 38(2) | 33(2) | -17(1) | 6(1) | -1(1) |
| C(26) | 28(2) | 47(2) | 52(2) | -27(2) | 0(1) | -2(1) |
| C(27) | 31(1) | 38(2) | 36(2) | -19(1) | 2(1) | -4(1) |
| C(28) | 26(1) | 31(1) | 32(1) | -15(1) | 3(1) | -4(1) |
| C(29) | 26(1) | 27(1) | 26(1) | -13(1) | 0(1) | 0(1) |
| C(30) | 30(1) | 27(1) | 29(1) | -14(1) | 2(1) | -1(1) |
| C(31) | 31(1) | 31(2) | 34(1) | -18(1) | 4(1) | -5(1) |
| C(32) | 28(1) | 35(2) | 31(1) | -19(1) | 0(1) | 0(1) |
| C(33) | 35(2) | 35(2) | 33(2) | -13(1) | -6(1) | 3(1) |
| C(34) | 31(1) | 31(1) | 30(1) | -14(1) | -1(1) | -2(1) |
| C(35) | 25(1) | 30(1) | 26(1) | -16(1) | 0(1) | 0(1) |
| C(36) | 53(2) | 28(2) | 42(2) | -11(1) | 10(2) | -4(1) |
| C(37) | 39(2) | 30(2) | 32(2) | -12(1) | 2(1) | 1(1) |
| C(38) | 28(1) | 32(2) | 34(2) | -18(1) | -3(1) | 5(1) |

**Table S 12. Hydrogen coordinates ( x $10^4$) and isotropic displacement parameters ($Å^2$x $10^3$) for DSOCT-$Br_6$.**

| | x | y | z | U(eq) |
|---|---|---|---|---|
| H(1AA) | 8472 | -1412 | 7642 | 189 |
| H(1AB) | 7626 | -1563 | 8581 | 189 |
| H(1AC) | 7043 | -980 | 7524 | 189 |
| H(2AA) | 8402 | 376 | 7131 | 133 |
| H(2AB) | 8995 | -218 | 8208 | 133 |
| H(1BA) | 8080 | -974 | 6720 | 189 |
| H(1BB) | 6864 | -236 | 6501 | 189 |
| H(1BC) | 8235 | 153 | 6460 | 189 |
| H(2BA) | 8412 | -889 | 8154 | 133 |
| H(2BB) | 7052 | -1274 | 8195 | 133 |
| H(1CA) | 8122 | -1189 | 6984 | 189 |
| H(1CB) | 7254 | -1637 | 7951 | 189 |
| H(1CC) | 6708 | -755 | 6975 | 189 |
| H(2CA) | 8171 | 318 | 7058 | 133 |
| H(2CB) | 8716 | -563 | 8032 | 133 |
| H(3A) | 6570(50) | -630(40) | 8920(40) | 112(19) |
| H(3B) | 7290(50) | 480(40) | 8530(40) | 93(18) |
| H(4A) | 5384 | 106 | 7526 | 81 |
| H(4B) | 6034 | 1140 | 7095 | 81 |
| H(5A) | 4542 | 339 | 8879 | 60 |
| H(5B) | 5187 | 1378 | 8441 | 60 |
| H(6A) | 3897 | 1941 | 7013 | 49 |
| H(6B) | 3185 | 933 | 7550 | 49 |
| H(13A) | 2324 | 6631 | 8258 | 39 |
| H(13B) | 1065 | 6744 | 8816 | 39 |
| H(14A) | 1216 | 7462 | 6736 | 45 |
| H(14B) | 7 | 7627 | 7316 | 45 |
| H(15A) | 1151 | 8645 | 7737 | 61 |
| H(15B) | 2402 | 8440 | 7227 | 61 |
| H(16B) | 250(50) | 9530(30) | 6150(30) | 70(13) |
| H(16A) | 1470(40) | 9360(30) | 5690(30) | 62(13) |
| H(17A) | 1574 | 10472 | 6701 | 105 |

| | | | | |
|---|---|---|---|---|
| H(17B) | 2384 | 10592 | 5761 | 105 |
| H(18A) | 983 | 11988 | 5357 | 209 |
| H(18B) | 612 | 11423 | 4769 | 209 |
| H(18C) | -201 | 11303 | 5712 | 209 |
| H(17C) | 1290 | 10457 | 6793 | 105 |
| H(17D) | 2594 | 10275 | 6321 | 105 |
| H(18D) | 1727 | 11890 | 5307 | 209 |
| H(18E) | 1773 | 11255 | 4728 | 209 |
| H(18F) | 469 | 11437 | 5201 | 209 |
| H(17E) | 1884 | 10735 | 5068 | 105 |
| H(17F) | 2728 | 10317 | 6026 | 105 |
| H(18G) | 1331 | 11682 | 5950 | 209 |
| H(18H) | 141 | 11101 | 5944 | 209 |
| H(18I) | 976 | 10688 | 6890 | 209 |
| H(22A) | 796 | 4014 | 5923 | 42 |
| H(27A) | 5835 | 3206 | 8132 | 41 |
| H(34A) | 3512 | 2803 | 9562 | 37 |
| H(37A) | 2974 | 6996 | 6019 | 42 |

**Table S 13. Torsion angles [°] for DSOCT-$Br_6$.**

| | |
|---|---|
| C(1A)-C(2A)-C(3)-C(4) | -88(3) |
| C(1B)-C(2B)-C(3)-C(4) | 44(8) |
| C(1C)-C(2C)-C(3)-C(4) | -62.5(16) |
| C(2A)-C(3)-C(4)-C(5) | -162.4(16) |
| C(2B)-C(3)-C(4)-C(5) | 153(3) |
| C(2C)-C(3)-C(4)-C(5) | -179.1(8) |
| C(3)-C(4)-C(5)-C(6) | -179.7(4) |
| C(4)-C(5)-C(6)-C(7) | -173.2(3) |
| C(5)-C(6)-C(7)-C(19) | -79.5(4) |
| C(5)-C(6)-C(7)-C(8) | 101.0(4) |
| C(19)-C(7)-C(8)-C(9) | -1.5(4) |
| C(6)-C(7)-C(8)-C(9) | 178.0(3) |
| C(19)-C(7)-C(8)-C(20) | -174.3(3) |
| C(6)-C(7)-C(8)-C(20) | 5.2(4) |
| C(7)-C(8)-C(9)-C(10) | 178.8(3) |
| C(20)-C(8)-C(9)-C(10) | -8.5(5) |
| C(7)-C(8)-C(9)-S(1) | 3.0(3) |
| C(20)-C(8)-C(9)-S(1) | 175.7(2) |
| C(19)-S(1)-C(9)-C(8) | -2.9(2) |
| C(19)-S(1)-C(9)-C(10) | -179.4(2) |
| C(8)-C(9)-C(10)-C(11) | -26.4(5) |
| S(1)-C(9)-C(10)-C(11) | 149.3(2) |
| C(8)-C(9)-C(10)-S(6) | 156.3(2) |
| S(1)-C(9)-C(10)-S(6) | -28.1(3) |
| C(38)-S(6)-C(10)-C(11) | -1.2(2) |
| C(38)-S(6)-C(10)-C(9) | 176.6(2) |
| C(9)-C(10)-C(11)-C(12) | -175.9(2) |
| S(6)-C(10)-C(11)-C(12) | 1.6(3) |
| C(9)-C(10)-C(11)-C(29) | 4.7(4) |
| S(6)-C(10)-C(11)-C(29) | -177.80(18) |
| C(10)-C(11)-C(12)-C(38) | -1.3(3) |
| C(29)-C(11)-C(12)-C(38) | 178.0(3) |
| C(10)-C(11)-C(12)-C(13) | 176.3(3) |
| C(29)-C(11)-C(12)-C(13) | -4.4(4) |
| C(38)-C(12)-C(13)-C(14) | -80.6(3) |
| C(11)-C(12)-C(13)-C(14) | 102.1(3) |

| | |
|---|---|
| C(12)-C(13)-C(14)-C(15) | -175.8(3) |
| C(13)-C(14)-C(15)-C(16) | 176.1(3) |
| C(14)-C(15)-C(16)-C(17C) | -160(2) |
| C(14)-C(15)-C(16)-C(17A) | 175(7) |
| C(14)-C(15)-C(16)-C(17B) | 176.8(12) |
| C(15)-C(16)-C(17A)-C(18A) | -149(8) |
| C(15)-C(16)-C(17B)-C(18B) | 178.9(14) |
| C(15)-C(16)-C(17C)-C(18C) | -97(4) |
| C(8)-C(7)-C(19)-S(1) | -0.7(4) |
| C(6)-C(7)-C(19)-S(1) | 179.7(2) |
| C(8)-C(7)-C(19)-Br(1) | 177.3(2) |
| C(6)-C(7)-C(19)-Br(1) | -2.3(5) |
| C(9)-S(1)-C(19)-C(7) | 2.1(3) |
| C(9)-S(1)-C(19)-Br(1) | -176.1(2) |
| C(29)-O(1)-C(20)-C(8) | 50.0(3) |
| C(29)-O(1)-C(20)-C(21) | 169.2(2) |
| C(29)-O(1)-C(20)-C(28) | -84.4(3) |
| C(9)-C(8)-C(20)-O(1) | 11.6(4) |
| C(7)-C(8)-C(20)-O(1) | -176.5(2) |
| C(9)-C(8)-C(20)-C(21) | -103.8(3) |
| C(7)-C(8)-C(20)-C(21) | 68.2(3) |
| C(9)-C(8)-C(20)-C(28) | 142.1(3) |
| C(7)-C(8)-C(20)-C(28) | -45.9(4) |
| O(1)-C(20)-C(21)-C(24) | 108.2(3) |
| C(8)-C(20)-C(21)-C(24) | -130.8(3) |
| C(28)-C(20)-C(21)-C(24) | -5.7(3) |
| O(1)-C(20)-C(21)-C(22) | -72.1(4) |
| C(8)-C(20)-C(21)-C(22) | 48.9(4) |
| C(28)-C(20)-C(21)-C(22) | 174.0(3) |
| C(24)-C(21)-C(22)-C(23) | 0.1(4) |
| C(20)-C(21)-C(22)-C(23) | -179.6(3) |
| C(21)-C(22)-C(23)-S(2) | -0.3(3) |
| C(21)-C(22)-C(23)-Br(3) | 176.9(2) |
| C(24)-S(2)-C(23)-C(22) | 0.3(3) |
| C(24)-S(2)-C(23)-Br(3) | -177.1(2) |
| C(22)-C(21)-C(24)-C(25) | -176.4(3) |
| C(20)-C(21)-C(24)-C(25) | 3.4(3) |
| C(22)-C(21)-C(24)-S(2) | 0.2(3) |

| | |
|---|---|
| C(20)-C(21)-C(24)-S(2) | 179.93(19) |
| C(23)-S(2)-C(24)-C(21) | -0.3(2) |
| C(23)-S(2)-C(24)-C(25) | 174.8(4) |
| C(21)-C(24)-C(25)-C(28) | 0.8(4) |
| S(2)-C(24)-C(25)-C(28) | -174.3(3) |
| C(21)-C(24)-C(25)-S(3) | 175.1(3) |
| S(2)-C(24)-C(25)-S(3) | 0.0(6) |
| C(26)-S(3)-C(25)-C(28) | 0.4(2) |
| C(26)-S(3)-C(25)-C(24) | -173.8(3) |
| C(25)-S(3)-C(26)-C(27) | -1.0(3) |
| C(25)-S(3)-C(26)-Br(2) | 177.0(2) |
| S(3)-C(26)-C(27)-C(28) | 1.4(3) |
| Br(2)-C(26)-C(27)-C(28) | -176.5(2) |
| C(24)-C(25)-C(28)-C(27) | 176.2(2) |
| S(3)-C(25)-C(28)-C(27) | 0.3(3) |
| C(24)-C(25)-C(28)-C(20) | -4.6(3) |
| S(3)-C(25)-C(28)-C(20) | 179.59(19) |
| C(26)-C(27)-C(28)-C(25) | -1.1(4) |
| C(26)-C(27)-C(28)-C(20) | 180.0(3) |
| O(1)-C(20)-C(28)-C(25) | -102.6(3) |
| C(8)-C(20)-C(28)-C(25) | 125.6(3) |
| C(21)-C(20)-C(28)-C(25) | 6.1(3) |
| O(1)-C(20)-C(28)-C(27) | 76.3(4) |
| C(8)-C(20)-C(28)-C(27) | -55.4(5) |
| C(21)-C(20)-C(28)-C(27) | -175.0(3) |
| C(20)-O(1)-C(29)-C(30) | 138.4(2) |
| C(20)-O(1)-C(29)-C(11) | -96.9(3) |
| C(20)-O(1)-C(29)-C(35) | 28.6(3) |
| C(10)-C(11)-C(29)-O(1) | 54.4(3) |
| C(12)-C(11)-C(29)-O(1) | -124.9(3) |
| C(10)-C(11)-C(29)-C(30) | 169.0(2) |
| C(12)-C(11)-C(29)-C(30) | -10.3(4) |
| C(10)-C(11)-C(29)-C(35) | -75.8(3) |
| C(12)-C(11)-C(29)-C(35) | 104.9(3) |
| O(1)-C(29)-C(30)-C(31) | -125.9(2) |
| C(11)-C(29)-C(30)-C(31) | 118.2(3) |
| C(35)-C(29)-C(30)-C(31) | -2.7(3) |
| O(1)-C(29)-C(30)-C(37) | 44.3(4) |

| | |
|---|---|
| C(11)-C(29)-C(30)-C(37) | -71.6(4) |
| C(35)-C(29)-C(30)-C(37) | 167.5(3) |
| C(37)-C(30)-C(31)-C(32) | -170.1(2) |
| C(29)-C(30)-C(31)-C(32) | 2.3(3) |
| C(37)-C(30)-C(31)-S(5) | 3.3(3) |
| C(29)-C(30)-C(31)-S(5) | 175.73(18) |
| C(36)-S(5)-C(31)-C(30) | -1.9(2) |
| C(36)-S(5)-C(31)-C(32) | 168.8(3) |
| C(30)-C(31)-C(32)-C(35) | -0.8(3) |
| S(5)-C(31)-C(32)-C(35) | -171.7(2) |
| C(30)-C(31)-C(32)-S(4) | -179.1(2) |
| S(5)-C(31)-C(32)-S(4) | 10.0(5) |
| C(33)-S(4)-C(32)-C(35) | -0.9(2) |
| C(33)-S(4)-C(32)-C(31) | 177.3(3) |
| C(32)-S(4)-C(33)-C(34) | -0.9(2) |
| C(32)-S(4)-C(33)-Br(4) | 173.25(18) |
| S(4)-C(33)-C(34)-C(35) | 2.4(3) |
| Br(4)-C(33)-C(34)-C(35) | -171.1(2) |
| C(31)-C(32)-C(35)-C(34) | -176.3(2) |
| S(4)-C(32)-C(35)-C(34) | 2.5(3) |
| C(31)-C(32)-C(35)-C(29) | -1.0(3) |
| S(4)-C(32)-C(35)-C(29) | 177.77(17) |
| C(33)-C(34)-C(35)-C(32) | -3.1(3) |
| C(33)-C(34)-C(35)-C(29) | -176.5(3) |
| O(1)-C(29)-C(35)-C(32) | 113.5(3) |
| C(30)-C(29)-C(35)-C(32) | 2.1(3) |
| C(11)-C(29)-C(35)-C(32) | -123.7(2) |
| O(1)-C(29)-C(35)-C(34) | -73.1(4) |
| C(30)-C(29)-C(35)-C(34) | 175.6(3) |
| C(11)-C(29)-C(35)-C(34) | 49.8(4) |
| C(31)-S(5)-C(36)-C(37) | 0.0(3) |
| C(31)-S(5)-C(36)-Br(5) | -179.3(2) |
| S(5)-C(36)-C(37)-C(30) | 1.8(3) |
| Br(5)-C(36)-C(37)-C(30) | -178.9(2) |
| C(31)-C(30)-C(37)-C(36) | -3.3(4) |
| C(29)-C(30)-C(37)-C(36) | -173.3(3) |
| C(11)-C(12)-C(38)-S(6) | 0.4(3) |
| C(13)-C(12)-C(38)-S(6) | -177.4(2) |

| | |
|---|---|
| C(11)-C(12)-C(38)-Br(6) | -178.97(19) |
| C(13)-C(12)-C(38)-Br(6) | 3.2(4) |
| C(10)-S(6)-C(38)-C(12) | 0.4(2) |
| C(10)-S(6)-C(38)-Br(6) | 179.83(17) |

Symmetry transformations used to generate equivalent atoms:

**Table S 14. Hydrogen bonds for DSOCT-$Br_6$a [Å and °].**

| D-H...A | d(D-H) | d(H...A) | d(D...A) | <(DHA) |
|---|---|---|---|---|
| C(22)-H(22A)...Br(3)#1 | 0.93 | 3.11 | 3.735(3) | 126.4 |

Symmetry transformations used to generate equivalent atoms:

#1 -x,-y+1,-z+1

## Reference


1. J. Tirado-Rives and W. L. Jorgensen, Performance of B3LYP density functional methods for a large set of organic molecules. *J. Chem. Theory Comput.*, **2008**, *4*, 297-306.
2. Noei M , Holoosadi M , Anaraki-Ardakani H .Design of methyldopa structure and calculation of -its properties by quantum mechanics. *Arab. J. Chem.* **2017**, *10*, S1923-S1937.
3. The 6-31G++ basis set: An economical basis set for correlated wavefunctions *J Comput. Chem.***1982,** *3*(4), 561-564
4. Bruker, S., APEX3 V2016. 9-0, SAINT V8. 37A. *Bruker AXS Inc.*, *Madison (WI)*, *USA* **2016**, 2013, 2014.
5. Sheldrick, G. M., Crystal structure refinement with SHELXL. *Acta. Crystallogr. C.*, **2015,** *71* (1), 3-8
6. Sheldrick, G. M., SHELXT–Integrated space-group and crystal-structure determination. *Acta. Crystallogr. A.*, **2015**, *71* (1), 3-8.
